# Plasticity leaves a phenotypic signature during local adaptation

**DOI:** 10.1002/evl3.185

**Published:** 2020-06-09

**Authors:** Reinder Radersma, Daniel W.A. Noble, Tobias Uller

**Affiliations:** ^1^ Department of Biology Lund University Lund Sweden; ^2^ Biometris Wageningen University & Research Wageningen The Netherlands; ^3^ Division of Ecology and Evolution, Research School of Biology The Australian National University Canberra ACT Australia

**Keywords:** adaptive plasticity, evolvability, genetic accommodation, phenotypic accommodation, phenotypic plasticity, plants, plasticity‐led evolution, P‐matrix, reciprocal transplant

## Abstract

Phenotypic responses to a novel or extreme environment are initially plastic, only later to be followed by genetic change. Whether or not environmentally induced phenotypes are sufficiently recurrent and fit to leave a signature in adaptive evolution is debated. Here, we analyze multivariate data from 34 plant reciprocal transplant studies to test: (1) if plasticity is an adaptive source of developmental bias that makes locally adapted populations resemble the environmentally induced phenotypes of ancestors; and (2) if plasticity, standing phenotypic variation and genetic divergence align during local adaptation. Phenotypic variation increased marginally in foreign environments but, as predicted, the direction of ancestral plasticity was generally well aligned with the phenotypic difference between locally adapted populations, making plasticity appear to "take the lead" in adaptive evolution. Plastic responses were sometimes more extreme than the phenotypes of locally adapted plants, which can give the impression that plasticity and evolutionary adaptation oppose each other; however, environmentally induced and locally adapted phenotypes were rarely misaligned. Adaptive fine‐tuning of phenotypes—genetic accommodation—did not fall along the main axis of standing phenotypic variation or the direction of plasticity, and local adaptation did not consistently modify the direction or magnitude of plasticity. These results suggest that plasticity is a persistent source of developmental bias that shapes how plant populations adapt to environmental change, even when plasticity does not constrain how populations respond to selection.

**Impact Summary**

Animal and plants are inherently responsive to their environments. Such “plasticity” can evolve, which explains why, for example, plants that grow in shade develop large leaves; modifying leaf size helps the plants to capture the right amount of light. While it is uncontroversial that responses to the environment can be adaptive, it is debated whether or not plasticity also directs genetic evolution. That is, do individual responses to a novel or extreme environment influence how populations adapt to this environment? Here, we analyze data from 34 studies of plasticity and local adaptation in plants to test if this is the case. Our results strongly suggest that locally adapted traits are modified versions of ancestrally plastic responses, making plasticity appear to “take the lead” in adaptive evolution. The immediate responses to the environment were sometimes more extreme than the phenotypes of locally adapted plants, which can give the false impression that plasticity and evolutionary adaptation oppose each other; in fact, truly maladaptive plasticity was rare. While the signature of plasticity persists during local adaptation, trait combinations could be modified independently of plasticity during genetic evolution. It thus appears that plasticity is an important source of adaptive developmental bias in plant evolution, which may facilitate rather than hinder adaptation.

Organisms are both responsive to their environment and locally adapted. Yet, the relationship between plasticity and evolution remains a matter of debate. To understand why, it can be helpful to view plasticity as a form of developmental bias that influences what phenotypes become available to selection (Uller et al. 2018; Parsons et al. 2020). If plastic responses to extreme or novel environments consistently shift phenotypes toward variants that are reasonably fit, local adaptation requires only limited genetic modification of those phenotypes. In such a situation, plasticity leaves a signature or may even appear to “take the lead” in adaptive evolution (Price et al. 2003; West‐Eberhard 2003; Frank 2011; Fig. 1A). However, environmentally induced phenotypes may be neither persistent nor fit. The phenotypic bias caused by plasticity may therefore quickly dissipate under natural selection, leaving no relationship between the environmentally induced phenotypes of ancestors and the locally adapted phenotypes of their descendants. Plasticity can even shift the phenotype distribution further away from a local optimum (Fig. 1A). For example, warm‐adapted organisms that colonize cooler climates will naturally slow down growth and development even if it often would be adaptive to have a faster life cycle (e.g., because of a shorter season, reviewed in Conover et al. 2009). The phenotypic differences between locally adapted populations and plastic responses may therefore not simply be unrelated, but actually opposite to each other.

**Figure 1:**
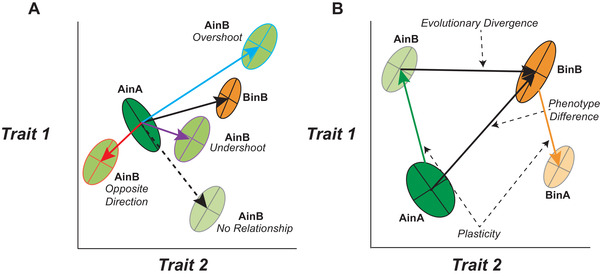
(**A)** Schematic of how the phenotype distribution (ellipses) in a population can shift in a foreign environment, and the relationship between plasticity and the phenotype of a population that is adapted to this environment (i.e., assumed to be at a local fitness maximum). The phenotypes produced by a plastic response (AinB; light green ellipses) of the ancestral population (AinA: dark green ellipse) can be unrelated, or more or less aligned or anti‐aligned with the phenotypes of the locally adapted population (BinB; orange ellipse). If the plastic response of the ancestral population (AinA) and the population difference (AinA vs BinB; see panel B) are aligned (i.e., angle < 90°), plasticity can either overshoot or undershoot the locally adapted phenotype. Note that evolutionary change can occur in opposition to the direction of plasticity in two scenarios; when the direction of plasticity is aligned with the locally adapted phenotype but plasticity ‘overshoots’ the local fitness maximum, and when the plastic response is opposite to the phenotypic difference between the locally adapted populations. (**B)** Graphical depiction of the studies and effect sizes estimated in this meta‐analysis. In all cases, two or more traits (in this case two; the *x*‐ and the *y*‐axes) were measured in plants from two populations, where seeds or seedlings were reciprocally transplanted and grown into each of the two environments. Ellipses indicate the multivariate trait means (centers) and covariances for each population‐by‐environment combination. Ancestral populations (“green”) and derived populations (“orange”) can be used to establish the relationship between plasticity and adaptation. Plasticity vectors can be generated by calculating the multivariate centroid differences between populations in their home environments (AinA or BinB – dark green and orange ellipses, respectively) compared to the same population grown in the foreign environment (AinB or BinA – light green and orange ellipses, respectively). The phenotypic difference between populations can be calculated by the multivariate centroid difference between each population in their home environment (AinA compared to BinB). The evolutionary divergence between population A and population B can be calculated by comparing the centroid differences between population A in environment B (AinB) and the locally adapted population B in environment B (BinB). The effect sizes calculated from these vectors and their alignment were used in the meta‐analysis (see Methods and Supplementary Methods for details).

While these scenarios focus on whether or not responses to the environment put the population close to a “fitness peak” (Price et al. 2003; Ghalambor et al. 2007; Frank 2011), plasticity can also exercise a more subtle effect on the phenotype distribution that is available to selection. Theory suggests that adaptive plasticity modifies the genotype–phenotype map such that the phenotypic effects of random genetic change resemble environmentally induced phenotypes (Draghi and Whitlock 2012; see also Lind et al. 2015; Noble et al. 2019). Thus, selection on correlated traits should be most effective when selection is aligned with the direction of plasticity, since those trait dimensions also will harbor the most additive genetic variation. Adaptive evolution should therefore be prone to follow “lines of least developmental resistance” where the phenotypic effects of environmental and genetic change are aligned.

There is evidence from both plants and animals that adaptation to a novel or extreme environment involves modification of plastic responses (a process sometimes referred to as “genetic accommodation”; West‐Eberhard 2003; for more recent reviews see Schlichting and Wund 2014; Ehrenreich and Pfennig 2016; Levis and Pfennig 2016; Schneider and Meyer 2017; Kelly 2019). For example, non‐native brownwort (*Prunella vulgaris*) adapted to understory express a phenotype that resembles the phenotype induced by low light conditions in native populations (Godoy et al. 2011). In spadefoot toads (*Spea* spp.), three out of four morphological traits that are exaggerated in carnivorous tadpoles were found to respond to a carnivorous diet in a closely related species that normally feeds on detritus (Levis et al. 2018). However, strong quantitative evidence that adaptive evolution involves fine‐tuning of plastic responses is still lacking (Levis and Pfennig 2020; Parsons et al. 2020). Furthermore, studies that attempt to test this hypothesis typically assess the relationship between plasticity and population or species divergence on a trait‐by‐trait basis. While this approach can be informative (e.g., to understand how populations track environmental change; Stoks et al. 2016), it provides limited insights on the role of developmental bias in evolution; the expression of a single trait can typically go both up and down, making it appear as if phenotypes can vary freely even when particular trait combinations are rare or impossible in development (Uller et al. 2018). Multivariate analyses therefore provide stronger tests of whether or not plastic responses are sufficiently recurrent and fit to leave a phenotypic signal in adaptive evolution.

Here, we make use of a vector‐based meta‐analysis (Adams and Collyer 2009; Fig. 1B) to capture and quantify the association between plasticity and the phenotypes of locally adapted populations. A similar approach has proven useful to conceptualize and quantify parallel evolution (Bolnick et al. 2018; Stuart et al. 2017). We relied on studies that used a reciprocal transplant design to establish phenotypic plasticity, phenotypic divergence between populations, and the fitness of individuals in local and foreign environments. An alignment between plasticity of ancestors and the phenotypes of locally adapted descendants would be consistent with a role for environmentally induced phenotypes in directing the course of adaptive evolution (West‐Eberhard 2003; Fig. 1A). An alignment between plasticity, standing phenotypic variation, and genetic divergence would go further, and point toward an intimate relationship between plasticity and genetic evolvability (Draghi and Whitlock 2012; Lind et al. 2015; Noble et al. 2019).

## Methods

### LITERATURE SEARCH AND STUDY INCLUSION

We did a literature search in Web of Science (search date: 25 November 2019) for studies containing (“local adaptation” AND reciprocal AND transplant^*^) in the title, abstract or keywords to find reciprocal transplant experiments. In addition, we selected primary studies collated by previous meta‐analyses investigating local adaptation: Leimu and Fischer (2008), Hereford (2009), Boshier et al. (2015), Palacio‐López et al. (2015), and Halbritter et al. (2018). We also searched in the Dryad Digital Repository (http://datadryad.org) for studies that deposited their data on this platform by using the search term “reciprocal transplant*” (search date: 11 March 2019).

We chose studies where seeds or plants of two (or more) populations (A and B) were transplanted to their own and the other population's environment, leading to four experimental units: population A in environment A (AinA), AinB, BinB, and BinA (Fig. 1B). To be included, studies had to demonstrate local adaptation, which we define as at least one fitness measure or fitness proxy being higher in at least one of the native environments compared to the transplant environment (Kawecki and Ebert 2004). A single study could contain several informative comparisons (e.g., if they included multiple populations or species). Additionally, studies had to measure at least two morphological or phenology traits that were considered important to the plants (e.g., leaf shape or timing of flowering), but not considered fitness proxies (e.g., survival or number of seeds). This criterion resulted in the exclusion of many studies, but also allowed for greater consistency in the studies included in our meta‐analysis (Appendix S1). As far as possible, we assigned population A to be the likely ancestral population or environment while B was the derived population or environment, which we identified by estimating the phylogenetic age for all populations within a study. This was based on reports in the primary studies or other publications, global patterns of colonization (e.g., south to north gradients on the Northern Hemisphere, lower to higher altitudes, center to edge of geographical distributions), benign versus more hostile environments or historical records of invasion or colonization. Because the history of the populations was ambiguous in some cases, we performed, for all main analyses, a test in which we randomized the ancestral order for all potential ambiguous studies. Results from our randomisation tests showed that this ambiguity did not affect our overall conclusions (see Appendix S1 and Figure S11 in Appendix S2 for more details). We estimated the strength of local adaptation of the “derived” population by calculating Hedges’ *g* for these fitness estimates between AinB and BinB (see Appendix S1 for more details).

### EFFECT SIZES COMPARISONS

We were primarily concerned with how the plastic response of population A to environment B (AinB) was related to the phenotypic differences between populations (AinA vs. BinB), and to the evolutionary divergence between populations A and B, which is revealed when both are grown in environment B (AinB vs. BinB; see Fig. 1B).

**Figure 2:**
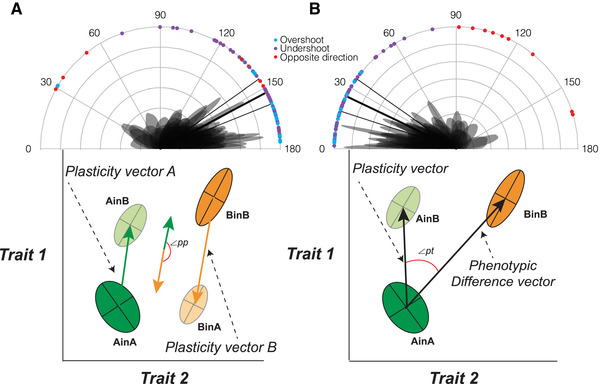
Plots of angles between (A) plastic response vector of population A (vector for AinA to AinB) and the plastic response vector of population B (vector for BinB to BinA) (denoted as ∠*pp*) and (B) the plastic response vector of population A (vector AinA to AinB) and the vector of phenotypic difference between locally adapted populations (vector AinA to BinB; “dark green” and “dark orange” ellipses) (denoted as ∠*pt*). For both (A) and (B), in grey are the density plots of the angles produced by the simulations for each comparison. Colored dots on the rim of the graphs are the means for each comparison. The color of the dots indicates for each comparison if the plasticity vectors pointed in the opposite direction (>90°) to the total phenotypic difference (in red), in the same direction but overshot (<90° and projection of plasticity vector on the total phenotypic difference was longer than total phenotypic difference; in blue), or in the same direction but undershot (<90° and projection of plasticity vector on the total phenotypic difference was shorter than total phenotypic difference; in purple). The thick and thin black lines are the meta‐analytic model mean estimates and their 95% confidence interval, respectively. Below each plot is a graphical depiction of the vectors being compared and the angles calculated between the vectors (∠*pp* or ∠*pt*). Note that the plasticity vectors in panel (A) are calculated such that the responses to environments are similar for the two populations when the vectors are close to anti‐parallel (i.e., angle = 180°).

**Figure 3:**
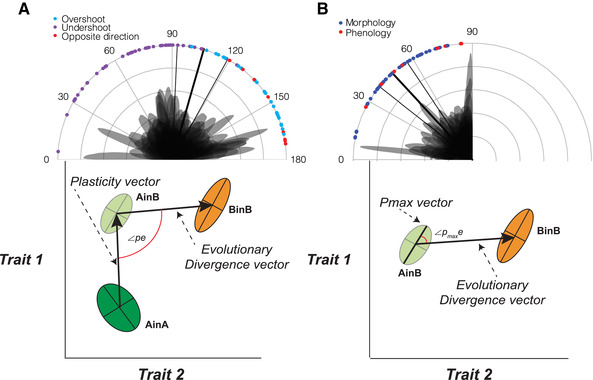
Plots of angles between (A) plasticity of population A (vector for AinA to AinB) and evolutionary divergence (vector for AinB and BinB) (∠*pe*), and (B) the first eigenvector of P matrix for AinB and evolutionary divergence (∠*p_max_e*). In grey are the density plots of the angles produced by the simulations for each comparison. Colored dots on the rim of the graphs are the means for each comparison. In (A), the color of the dots indicates for each comparison whether the plasticity vectors pointed in the opposite direction (>90°) to the total phenotypic difference (in red), in the same direction but overshot (<90° and projection of plasticity vector on the total phenotypic difference was longer than total phenotypic difference; in blue) or in the same direction but undershot (<90° and projection of plasticity vector on the total phenotypic difference was shorter than total phenotypic difference; in purple). In (B), the color of the dots indicates for each comparison whether the majority of traits are morphological (blue) or phenological (red) traits. The thick and thin black lines are the meta‐analytic model mean estimates and their 95% confidence interval, respectively. Below each plot is a graphical depiction of the vectors being compared and the angles calculated between the vectors (∠*pe* or ∠*p_max_e*).

In phenotype space, we constructed vectors between the multivariate mean trait values of the experimental units and used these vectors to compare angles and vector lengths as our effect size measures, accounting for sampling variation across studies (Fig. 1B). The vector between AinA and BinB describes the total phenotypic difference between the two populations in situ (Fig. 1B). While it can be difficult to rule out selective mortality as a contributor to the phenotypic difference between plants from a single origin grown in two locations (not all studies were able to conclusively do so), we assumed that this difference largely represents direct environmental effects on the phenotype. We therefore defined phenotypic plasticity for populations A and B as the vector between AinA and AinB, and BinB and BinA, respectively (Fig. 1B). Evolutionary divergence between populations A and B were defined as the vector between AinB and BinB, which is likely to reflect genetic divergence that accumulated during adaptation to environment B (Fig. 1B). Using these vectors, we derived a series of vector comparisons and used these as our effect size estimates. A comparison of the plasticity vector of population A (AinA to AinB) with the plasticity vector of population B (BinB to BinA) is a measure of the divergence in plastic responses between populations (denoted as ∠pp; Fig. 2A). A comparison of the plasticity vector of population A (AinA to AinB) with the phenotype difference vector (AinA to BinB) is a measure that describes the alignment between an ancestral population's plastic response and the total trait divergence between populations in their respective environments (denoted as ∠pt; Fig. 2B). To characterize the relationship between plasticity and evolutionary divergence, we quantified the angle between the plastic response vector for the ancestral population (AinA to AinB) and the evolutionary divergence vector between trait means (AinB to BinB) (denoted as ∠pe; Fig. 3A). Finally, we also tested whether evolutionary divergence follows the direction of maximum phenotypic (co)variance by comparing the first eigenvector of the phenotype distribution of the ancestral population in the derived environment (AinB) with the evolutionary trait divergence vector (AinB to BinB; denoted as ∠p_max_e in Fig. 3B). See Appendix S1 for more details on the equations used to derive all effect size metrics and how we accounted for effect size sampling variance.

### META‐ANALYSIS

We analyzed effect sizes using multi‐level meta‐analytic, and meta‐regression models (Nakagawa and Santos 2012) with the R package *metafor* (Viechtbauer 2010). In all models, effects were weighted by their sampling variance from the Monte Carlo simulations of P‐matrices and trait means based on each population's sample size (See Appendix S1 for more details). Given that there was a strong correspondence between effect sizes derived from a single study and the species in our dataset (i.e., study and species were confounded), we first explored whether a model that included either a study or species level random effect (accounting for phylogenetic relationships) best explained variation in effect sizes. To achieve this, we fitted multilevel meta‐analytic models that included either study or phylogeny (i.e., phylogenetic correlation matrix) as random effects and compared models using AICc. Phylogenetic correlation matrices were derived by generating dated phylogenies for the species in the data set using TimeTree.org (Hedges et al. 2015). Time Tree uses a global database of published time calibrated trees from over 2000 published phylogenies (Hedges et al. 2015). We then used the packages *phytools* (Revell 2012) and *ape* (Paradis and Schliep 2019) to visualize the tree, ensuring no polytomies existed, and generate the phylogenetic correlation matrix. For taxa not identified in the time tree database, we choose its mostly closely related taxa that was in the database. The final tree was pruned to include only the species in each data set. In most cases, the inclusion of a study‐level random effect was equally or better supported then models with the phylogeny (See Table S1.1 in Appendix S1). As such, we present meta‐analytic models that estimate only a between study variance because this is most often modeled in meta‐analyses (Nakagawa and Santos 2012; Nakagawa et al. 2017). The package *metafor* does not estimate a residual variance by default so we also included an observation‐level random effect in all our models. In some studies, the reciprocal transplants were conducted between more than two environments and we used the same environment to generate multiple effect sizes introducing “shared‐group” non‐independence among effect sizes (Noble et al. 2017). This is analogous to situations where effect sizes share control groups. As such, we included a modified sampling variance matrix fitting the off‐diagonal covariances (assuming a correlation, *r* = 0.5) in the sampling covariance matrix.

We also ran meta‐regression models to explore sources of variance among effect sizes. For each effect size, we included main effects for moderators we hypothesized would explain variation in effects when sample sizes allowed. These included (i) the number of traits measured, (ii) the proportion of those traits classified as morphology rather than phenology (our two “trait types”), and (iii) the extent of local adaptation (see Appendix S1 for more description on these moderator variables). Given the limited sample sizes we restricted models to estimating main effects of the above moderators only (i.e., no interactions).

## Results

We identified 34 reciprocal transplant studies that met our criteria, using 34 plant species from 32 genera and 14 orders. The number of comparisons per study varied greatly from 1 to 15, with most studies consisting of only one comparison (*N* = 23). The number of phenotypic traits measured across studies varied from two to nine traits (mean = 3.82, SD = 1.91, *N* = 34). Twenty‐two studies measured exclusively morphological traits, while three measured phenological traits only and nine studies measured both types.

### PLASTICITY AND PHENOTYPIC VARIATION IN LOCALLY ADAPTED POPULATIONS

Within experimental units, the phenotypic variation was higher for AinB than for AinA, although confidence intervals were wide (+11.03%, CI: −1.41% to 23.46%; §4.1 and 4.2 in Appendix S2). Nonetheless, this is consistent with the expectation that phenotypic variation should increase in novel or extreme environments. The distribution of the trait combinations (i.e., shape of the P‐matrices) remained similar when individuals were translocated to a different environment, as evident from the positive correlations between the ratios of the second and first eigenvectors of the P‐matrix for AinA and AinB (ρ= 0.61; 95% CI: 0.47 to 0.73), for BinB and BinA (ρ= 0.62; 95% CI: 0.49 to 0.73), and for AinA and BinB (ρ= 0.50, 95% CI 0.34 to 0.63; §4.3 in Appendix S2).

Plastic responses of populations A and B were generally similar (i.e., ∠pp; Fig. 2A), suggesting that local adaptation has limited effect on how populations respond to the relevant environmental factors. Specifically, the trait correlations observed in plastic responses were very similar, as reflected by the fact that the two plasticity vectors were close to anti‐parallel (mean angle of 152.37°; 95% CI: 145.17° to 158.30°; Fig. 2A; §4.4 in Appendix S2), and the vectors did not differ in their standardized length (the average difference in length of plasticity vector B and plasticity vector A was −0.0070; 95% CI: −0.1723 to 0.1583 on the log scale). The number of traits and the strength of local adaptation did not impact these estimates (§4.5 in Appendix S2).

Previous work has suggested that the plastic response in two or more traits tends to be aligned with the standing genetic variation (Noble et al. 2019). We therefore tested if the average angle between the plasticity vector (AinA to AinB) and the first eigenvector of the P‐matrices of AinA (P_max_) were aligned. This angle would be zero if the two are fully aligned and 45° if the two are oriented randomly with respect to each other. The average observed angle was 54.52° (95% CI: 44.91° to 63.31°), indicating that plasticity did not follow the axis of most phenotypic variation in this sample of studies. For studies with fewer traits, plasticity and the main axis of phenotypic variation aligned better than studies with more traits (one SD less resulted in a mean angle of 36.99°, while one SD more resulted in an angle of 59.12°; §4.6 in Appendix S2).

### ALIGNMENT BETWEEN PLASTICITY AND LOCALLY ADAPTED PHENOTYPES

The average angle between the plasticity vector and the phenotypic trait difference between populations grown under their locally adapted condition (i.e., ∠pt; Fig. 2B) was 25.32° (95% CI: 17.99 −34.99°; Fig. 2B; §5.1 in Appendix S2), and thus aligned better than at random (90°). On average, 73.95% (95% CI: 55.25–92.65) of the length of the phenotypic difference vector could be accounted for by phenotypic plasticity. This effect was stronger for morphological (on average 85.44%) than for phenological traits (on average 21.86%; §5.2 in Appendix S2). This reflects that phenology traits were more likely to respond in a direction that is more or less opposite to the phenotypic difference between local adapted populations (Appendix S1). In total, nine plasticity vectors pointed in the opposite direction (i.e., > 90°) to the total phenotypic difference and, out of the remaining 72 plasticity vectors, 25 “overshot” the total phenotypic difference (i.e., resulting in more than 100% of the phenotypic difference between populations being accounted for by phenotypic plasticity; see Fig. 1B, empirical examples in Appendix S3).

### ALIGNMENT BETWEEN PLASTICITY AND EVOLUTIONARY DIVERGENCE

The evolution of plasticity can shape the distribution of genetic covariance between traits (Draghi and Whitlock 2012), which in turn can influence the extent to which populations will respond to selection (Hansen and Houle 2008). To assess the relationship between plasticity and evolutionary divergence, we compared the angle between the plasticity and evolutionary divergence vectors (i.e., ∠pe; Fig. 3A). This angle would be zero if the two are fully aligned, 180° if they were opposite in direction, and 90° if they two are oriented randomly with respect to each other. The average angle was 105.97° (95% CI: 92.26 to 118.95°; Fig. 3A; §6.1 in Appendix S2), indicating that, on average, they were slightly less aligned than at random (90°). This result reflects the combined effect of the studies in which plastic responses are opposite in direction to the total divergence (9 out of 81 plasticity vectors; Fig. 7 in Appendix S2), and the studies in which the vector of plastic responses of population A are aligned with the vector of phenotypic difference between populations (i.e., < 90°), but “overshoot,” the phenotypes of population B (25 out of 81 estimates; Fig. 3A). Many estimates were close to that expected at random (i.e., 90°; see Fig. 3A).

We also tested the hypothesis that evolutionary divergence follows the direction of maximum phenotypic (co)variance (e.g., Schluter 1996). To test this, we estimated the angle between evolutionary divergence and the first eigenvector (P_max_) of the phenotypic covariance matrix (P‐matrix) in the environment in which selection would have occurred (i.e., AinB rather than AinA). This angle can only take values between 0° and 90° since eigenvectors are not directed. An angle of 45° is therefore considered random. Maximum phenotypic variance of AinB was not aligned with evolutionary divergence, with an angle of 47.90° (95% CI: 38.79° to 56.79°), but the angle was on average smaller (36.17°) for studies with only morphological traits than for studies with only phenology traits (73.18°; Fig. 3B; §6.3 in Appendix S2).

## Discussion

There has been much controversy over whether or not the phenotypes induced by novel or extreme environments are sufficiently fit and persistent to leave a signature in adaptive evolution (West‐Eberhard 2003; Laland et al. 2015; Ho and Zhang 2017; Van Gestel and Weissing 2018; Kovaka 2019; Uller et al. 2019; Parsons et al. 2020). Plant reciprocal transplant studies strongly suggest that they are. The phenotypes of locally adapted plants typically resemble the phenotypes induced by the local environment in a likely ancestor. This provides evidence that adaptation involves fine‐tuning of environmentally induced phenotypes (“genetic accommodation”; West‐Eberhard 2003), making plasticity appear to take the lead in adaptive evolution.

The overall close alignment between plasticity and the phenotypic difference between locally adapted populations suggests that responses to the environment are an important source of adaptive variation in plants. One explanation for this is that the environmental variables that differ between populations commonly also vary within populations (e.g., on a small spatial scale or between years), and therefore promotes the evolution of adaptive plasticity. For example, many plants have evolved mechanisms that enable them to adjust leaf morphology and physiology in response to sunlight, water availability, or mechanical stress (Chitwood and Sinha 2016; Fritz et al. 2018). If those plants encounter conditions that are sunnier, drier, or windier than what they are adapted to, this mechanism may not only enable individuals to persist, but will consistently provide natural selection with a particular subset of the many possible phenotypes that could be adaptive in those conditions. Through this logic, populations that have evolved adaptive plasticity should continue to adapt by modifying the mechanisms and traits that are environmentally responsive, rather than inventing new adaptations. As a result, evolution will tend to go where plasticity leads. The extent to which populations really do adapt to novel or extreme environments using the same mechanisms and traits that they employ in adaptive plasticity is poorly understood, however. Plants, and perhaps in particular the morphology and physiology of leaves or roots, should make for good models since much is known about the mechanisms of plasticity and adaptive genetic divergence between populations and species (e.g., Fritz et al. 2018).

A plastic response that puts individuals closer to a local fitness maximum will be followed by adaptive evolutionary change only insofar as there is heritable variation for the trait combinations that are fit. Foreign environments appeared to have a small positive effect on the total phenotypic variation, which may reflect the release of “cryptic genetic variation” that is sometimes considered important to plasticity‐led evolution (e.g., Levis and Pfennig 2016). There was no evidence that the phenotypic variation was structured in accordance with the direction of plasticity, despite that development is expected to cause plasticity and the main axis of genetic variation to be aligned (Draghi and Whitlock 2012; Lind et al. 2015; Noble et al. 2019). Furthermore, there was no evidence that evolutionary (genetic) divergence was biased either toward the main axis of standing phenotypic variation or the direction of plasticity. While a more appropriate estimate of the evolutionary potential of populations in foreign environments would have been the additive genetic covariance, genetic and phenotypic covariances are commonly very similar (e.g., Roff 1996; Noble et al. 2019). The lack of relationships between plasticity, main axis of standing phenotypic variation, and evolutionary divergence suggest ample capacity for fine‐tuning of phenotypes during local adaptation, and that responses to selection are largely unconstrained by how organisms respond to a novel or extreme environment. However, we acknowledge that the traits measured in studies of local adaptation may not necessarily be developmentally or functionally integrated with each other, which limits the ability to detect a relationship between plasticity and genetic evolvability. Furthermore, some studies may have included traits that are only weakly selected in the relevant environments. Such traits will diverge largely as a result of stochastic events, making comparisons between plasticity and evolutionary divergence relatively uninformative with respect to the plasticity‐first hypothesis (but they could still reflect an alignment between plasticity and standing genetic variation). To overcome the limits of purely comparative studies, it would be informative to contrast how natural or experimental populations with different degrees or forms of plasticity respond to selection for different trait combinations. Targeted empirical studies are also needed to establish the relationship between the direction and magnitude of plasticity and the rate and magnitude of genetic divergence between populations (Crispo 2008; Schmid and Guillaume 2017)

While plasticity generally was well aligned with the phenotypic difference between locally adapted populations, the latter was sometimes less extreme (i.e., plasticity “overshoots” the phenotypes of locally adapted populations; Fig. 1A). The present study is unable to identify any conditions that predict this situation, but it is perhaps equally likely that a plastic response to a foreign environment will over‐ as under‐shoot a local fitness peak. In contrast, plastic responses rarely fell in the opposite direction to the phenotypic difference between locally adapted populations. When this did happen, it involved phenology rather than morphology. Timing of germination or flowering tends to rely on inherently temperature‐dependent processes, and it is not unexpected that adaptation to climate can require substantial modification of responses to temperature and other cues (Conover et al. 2009). However, judging from the studies included in this meta‐analysis, it is not very commonly the case that plasticity is strongly maladaptive in this sense, and some instances may be an artefact of how phenology is coded (e.g., using “days” rather than “growing degree days”; see Ensing and Eckert 2019).

That plasticity and evolutionary change occur in opposite directions appears to be common in transcriptomic data, where the pattern has been interpreted as evidence against a facilitating role for plasticity in adaptation (Ho and Zhang 2018; see also Ghalambor et al. 2015). There are problems with drawing inferences about the relationship between plasticity and evolution from analyses of gene expression (e.g., issues arising from treating single genes as plastic vs. non‐plastic; see Mallard et al. 2018; Van Gestel and Weissing 2018; Ho and Zhang 2019), but it is evident that “reversals” can happen even when plasticity puts the population closer to an adaptive phenotype (see Fig. 1A). Thus, it is not possible to tell whether plasticity facilitates or hampers adaptation from the direction of plastic and evolutionary responses alone, and this limitation is exacerbated by univariate analyses. Furthermore, since the phenotype and fitness of an individual with a particular gene expression profile is typically unknown, it appears impossible to infer that a population of environmentally responsive individuals will adapt more readily than a population with less responsive individuals, or vice versa. Similar caveats apply to studies that rely on more traditional traits, such as the ones included in this meta‐analysis. Strong inference on the role of plasticity in evolution will therefore benefit from knowledge about the fitness of the phenotypes produced by different (e.g., plastic vs. non‐plastic) developmental systems (Kovaka 2019; Uller et al. 2019). Thus, while the results from reciprocal transplant studies suggest that plasticity is an important source of developmental bias during plant local adaptation, it remains to be shown that plasticity in general facilitates adaptation.

The magnitude and direction of plasticity were, on average, similar between populations adapted to different environments. Since we attempted to consistently classify populations according to whether they occupied ancestral or derived environments, we interpret this result to imply that marginal populations (e.g., those in an environment that is more extreme for the species) are neither more nor less plastic in general. An increase in plasticity in marginal or more recently colonized habitats is expected in the early stages of “plasticity‐led” evolution (e.g., Lande 2009), but population differences in plasticity could also be maintained by gene flow (Crispo 2008; Chevin and Lande 2011). Conversely, a reduction in plasticity in extreme environments may be expected if these environments impose relatively strong stabilizing selection. Our results suggest that none of these scenarios are dominant in plants, but detection of multiple, conflicting, patterns in a meta‐analysis can be difficult. In fact, inspection of individual studies suggests substantial heterogeneity that could reflect that all of these scenarios exist (see Supporting Information), but that they cancel each other out when aggregated. To make progress in the field, it will be important to conduct studies of systems where *a priori* expectations can be made (e.g., on the basis of known colonization history and gene flow).

## Conclusion

The evidence from reciprocal transplant experiments suggests that plasticity is an evolutionarily significant source of developmental bias that makes plasticity appear to take the lead in plant adaptation. As ecologists become increasingly interested in evolvability (e.g., Sultan 2015; Hendry 2017), there is an urgent need for studies that can provide answers to questions such as “does plasticity make populations better able to adapt?” and “how does plasticity influence what kinds of environmental change populations can adapt to?” Such questions cannot easily (if at all) be answered simply by comparing plastic and evolutionary responses; they require comparison of the adaptive potential of populations that differ in how they generate phenotypic variation. We therefore anticipate a growth in the number of studies that combine mechanistic studies of the developmental basis of phenotypic variation with measures of selection in ecologically relevant settings.

## AUTHOR CONTRIBUTIONS

R.R., D.W.A.N., and T.U. designed the study; R.R., D.W.A.N., and T.U. collected the data; R.R. and D.W.A.N. analyzed the data; R.R., D.W.A.N., and T.U. interpreted results; and R.R., D.W.A.N., and T.U. wrote the paper.

## DATA ARCHIVING

All raw data, code, and analyses have been deposited in the Open Science Framework repository at https://osf.io/se53c/.

Associate Editor: A. Charmantier

## Supporting information

Appendix S1: Extended Materials and Methods

Appendix S2: Code and Analyses

Appendix S3: Plots of Empirical Examples
