# Supplementary material for: Plasticity leaves a phenotypic signature during local adaptation
Source: Evol Lett. 2020 Jun 9;4(4):360–70. doi: 10.1002/evl3.185 (PMC7403707; doi:10.1002/evl3.185)
Supplement: Supplementary file 1 — Appendix S1: Extended Materials and Methods [file EVL3-4-360-s001.pdf]

# Appendix S1: Plasticity takes the lead in local adaptation

Reinder Radersma, Daniel W.A. Noble & Tobias Uller

Manuscript for *Evolution Letters*

## Extended Materials and Methods

### *Literature Searches and Data Collection*

We searched the Web of Science (search data: 25 November 2019) for studies containing ‘"local adaptation" AND reciprocal AND transplant*’ in the title, abstract or keywords to find reciprocal transplant experiments. In addition, we selected primary studies collated by previous meta-analyses investigating local adaptation: Leimu & Fischer (2008), Hereford (2009), Boshier *et al.* (2015), Palacio-López *et al.* (2015) and Halbritter *et al.* (2018). We also searched in Dryad Digital Repository (http://datadryad.org) for studies which deposited their data on this platform by using the search term “reciprocal transplant*” (search date: 11 March 2019).

To be included in our meta-analysis, the primary study had to: 1) conduct a reciprocal transplant experiment between two or more environments; 2) show that there was local adaptation in at least one of the environments (or refer to another study that did show this), and 3) measure two or more phenotypic traits, other than the trait(s) which were used to establish local adaptation. Based on title and abstract relevant studies were selected (for the search in Web of Science by RR and TU, for the other sources by RR). The studies were more closely screened and relevant data was extracted (by RR and DWAN). In instances where only part of the data was available we checked if raw data was deposited in repositories, or contacted authors directly for the relevant information. For details, see the full PRISMA diagram (**Fig. S1.1**). All raw data, code and analyses can be found at Open Science Framework repository at https://osf.io/se53c/.

### *Moderator (Predictor) Variables*

We collected a number of moderator variables which we *a priori* expected to explain variation in effect sizes. These were the number of phenotypic traits quantified, the proportion of those traits being phenology traits (a range from 0 for only morphological traits to 1 for only phenology traits) and a measure of the extent of local adaptation. To calculate a measure for local adaptation we first identified the order of phylogenetic age for all populations. This order was based on reports in the primary studies or other publications, or global patterns of colonization such as south to north gradients on the Northern Hemisphere, lower to higher altitudes, centre to edge of geographical distributions or benign versus more hostile environments, or historical records of invasion or colonisation. Next we calculated, for all fitness traits, Hedges’ g between the youngest population in the youngest environment (BinB) and the oldest population in the youngest environment (AinB). For fitness traits that were expressed as percentages we first calculated the odds ratios and transformed those to Hedges’ g. A positive Hedges’ g means the new population is locally adapted, while a negative value means that the old population is better adapted in the new environment than the young population.

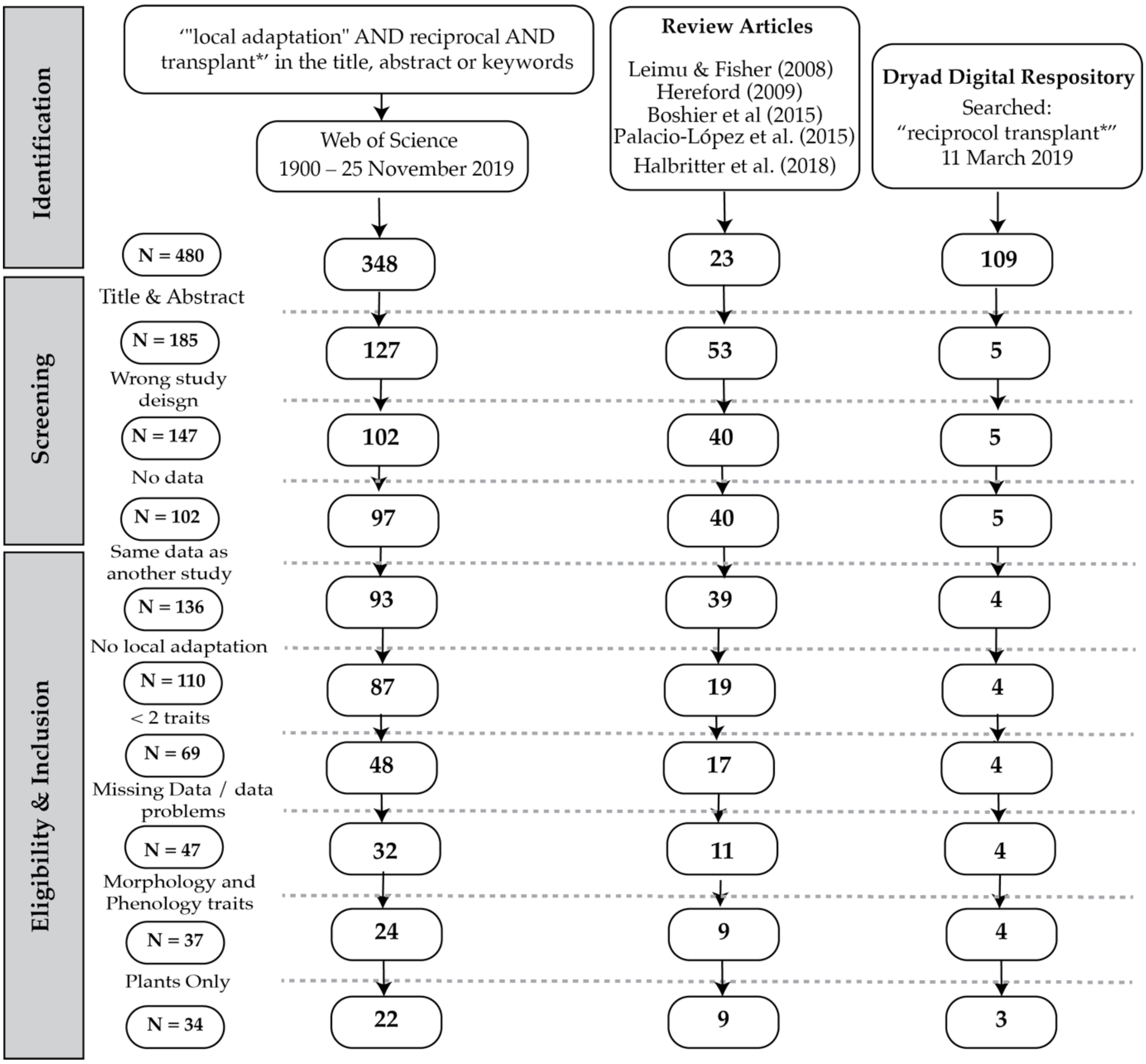


Figure S1.1: PRISMA diagram for the systematic search

*Effect Size Calculations*

To ensure that a few traits were not disproportionately impacting effect sizes, we centered and standardized, means, and P-matrices in such a way that the means and standard deviations for population A in environment A were zero and one, respectively. We centered and standardized means, as:

$$\overline{\boldsymbol{x}}_{\mu} \equiv \frac{\overline{\boldsymbol{x}} - \overline{\boldsymbol{x}}_{AinA}}{\boldsymbol{\sigma}_{AinA}}$$

in which $\overline{\boldsymbol{x}}_{AinA}$ are the means for the population A in environment A and $\boldsymbol{\sigma}_{AinA}$ their standard deviations. We standardized P-matrices, as:

$$\boldsymbol{P}_{\mu} \equiv \boldsymbol{P} \oslash (\boldsymbol{\sigma}_{AinA} \cdot \boldsymbol{\sigma}_{AinA}')$$

$\oslash$ denotes an element-wise division. Below we describe in detail how we calculated different effect sizes using the standardized means and covariance matrices, but to simplify notation, we drop the subscript $\mu$ in the remainder of the text.

We first constructed vectors between the simulated trait means. We calculated phenotypic plasticity of population A ($\boldsymbol{p}_A$) as the difference between the phenotypic trait means of population A in environment B and environment A:

$$\boldsymbol{p}_A = \overline{\boldsymbol{x}}_{AinB} - \overline{\boldsymbol{x}}_{AinA}$$

Similarly, phenotypic plasticity of population B ($\boldsymbol{p}_B$) is the difference between the phenotypic trait means of population B in environment A and environment B and defined as:

$$\boldsymbol{p}_B = \overline{\boldsymbol{x}}_{BinA} - \overline{\boldsymbol{x}}_{BinB}$$

The evolutionary divergence of population B ($\boldsymbol{e}_B$) is the difference between the phenotypic trait means of population A and population B in environment B and defined as:

$$\boldsymbol{e}_B = \overline{\boldsymbol{x}}_{AinB} - \overline{\boldsymbol{x}}_{BinB}$$

The total divergence of population B ($\boldsymbol{t}_B$) is the difference in between the phenotypic trait means of population B in environment B and population A in environment A and defined as:

$$\boldsymbol{t}_B = \overline{\boldsymbol{x}}_{BinB} - \overline{\boldsymbol{x}}_{AinA}$$

From these vectors we calculated effect sizes. To investigate whether phenotypic plasticity was reversible between populations A and B, we calculated the difference in length of plasticity vector B and A relative to the length of plasticity vector A ($|pp|$):

$$|pp| = \frac{|\boldsymbol{p}_B| - |\boldsymbol{p}_A|}{|\boldsymbol{p}_A|}$$

We also calculated the angle between plasticity of population A and population B ($\angle pp$) indicating their alignment and defined as:

$$\angle pp = acos\left(\frac{\boldsymbol{p}_A \cdot \boldsymbol{p}_B}{\sqrt{\boldsymbol{p}_A \cdot \boldsymbol{p}_A}\sqrt{\boldsymbol{p}_B \cdot \boldsymbol{p}_B}}\right)\frac{180}{\pi}$$

To test for the alignment between plasticity and the phenotypic difference between populations we calculated the angle between plasticity of population A and total divergence ($\angle pt$) and defined as:

$$\angle pt = acos\left(\frac{\boldsymbol{t}_B \cdot \boldsymbol{e}_B}{\sqrt{\boldsymbol{t}_B \cdot \boldsymbol{t}_B}\sqrt{\boldsymbol{e}_B \cdot \boldsymbol{e}_B}}\right)\frac{180}{\pi}$$

We calculated the projection of phenotypic plasticity on the total divergence relative to total divergence ($p{:}\,t$) as a measure of how much phenotypic plasticity contributed to local adaptation, which is defined as:

$$p{:}\,t = \frac{\boldsymbol{p}_A \cdot \boldsymbol{t}_B}{\boldsymbol{t}_B \cdot \boldsymbol{t}_B}$$

From this we classified studies as being undershot ($0 < p{:}\,t < 1$), overshot ($p{:}\,t \geq 1$) or in the opposing direction ($p{:}\,t \leq 0$). We used the angle between plasticity of population A and evolutionary divergence B ($\angle pe$) indicating their alignment and defined as:

$$\angle pe = acos\left(\frac{\boldsymbol{p}_A \cdot \boldsymbol{e}_B}{\sqrt{\boldsymbol{p}_A \cdot \boldsymbol{p}_A}\sqrt{\boldsymbol{e}_B \cdot \boldsymbol{e}_B}}\right)\frac{180}{\pi}$$

To investigate whether evolutionary divergence proceeds in the direction of most phenotypic variance we calculated the angle between the first eigenvector of the P matrix for AinA ($\boldsymbol{\lambda}_{max}$) and evolutionary divergence B ($\angle \lambda e$) indicating their alignment and defined as:

$$\angle \lambda e = acos\left(\frac{\boldsymbol{\lambda}_{max} \cdot \boldsymbol{e}_B}{\sqrt{\boldsymbol{\lambda}_{max} \cdot \boldsymbol{\lambda}_{max}}\sqrt{\boldsymbol{e}_B \cdot \boldsymbol{e}_B}}\right)\frac{180}{\pi}$$

Since $\boldsymbol{\lambda}_{max}$ is nondirectional, we subtracted $\angle \lambda e$ from 180 when it was larger than 90º. To test whether evolutionary divergence aligned better with most phenotypic variance rather than with phenotypic plasticity we calculated the difference between $\angle pe$ and $\angle \lambda e$ ($\Delta pe\lambda e$):

$$\Delta pe\lambda e = \begin{cases} \angle pe - \angle \lambda e, if\ \angle pe \leq 90° \\ 180 - \angle pe - \angle \lambda e, if\ \angle pe > 90° \end{cases}$$

To investigate whether plasticity proceeds in the direction of most phenotypic variance we calculated the angle between the first eigenvector of the P matrix of AinA and phenotypic plasticity A ($\angle \lambda p$):

$$\angle \lambda p = acos\left(\frac{\boldsymbol{\lambda}_{max} \cdot \boldsymbol{p}_A}{\sqrt{\boldsymbol{\lambda}_{max} \cdot \boldsymbol{\lambda}_{max}}\sqrt{\boldsymbol{p}_A \cdot \boldsymbol{p}_A}}\right)\frac{180}{\pi}$$

Since $\boldsymbol{\lambda}_{max}$ is nondirectional, we subtracted $\angle \lambda p$ from 180 when it was larger than 90º.

*Sampling Variance for Effect Sizes*

We took a flexible meta-analytic approach to effect size and sampling variance estimation that allowed us to make use of traditional multi-level meta-analytic models, which more effectively weight studies based on their precision. Given that no effect size captures all aspects, we used a series of alternative effect size measures and generated their corresponding sampling variances. For only part of the primary studies we had P-matrices available, therefore we built two sets of effect sizes; one for all effect sizes which were based on mean trait values and one for all effect sizes which were also based on P-matrices. The second dataset is based on a subset of all primary studies in the first dataset. For both sets we simulated observations to estimate the sampling variance. Prior to the simulations, any non-positive-definite P-matrices (i.e., non-positive eigenvalues) were 'bent' to make them positive definite (Wood & Brodie 2015). For both sets we excluded any phenotypic trait which did not describe a morphological or timing

related characteristic and where variances were zero or negative and which had a sample size smaller than 10.

To generate sampling variance we used Monte Carlo simulations to generate 5000 simulated datasets. For each trait in each experimental population we sampled individuals that matched the study sample size from a normal distribution with the observed mean and standard deviation of the population. For the subset of studies with P-matrices we repeated these simulations, but sampled for all traits simultaneously from a multivariate normal distribution with trait means as means and the P-matrices as the variance-covariance matrix. We drew as often as the largest sample size for each experimental population and randomly removed draws for individual traits when their sample sizes were smaller to simulate missing values. For all these simulated datasets we calculated effect sizes (see below), leading to distributions rather than point estimates. Effect sizes generated in this way have the benefit of propagating sampling variance from different study designs across different environments.

The ancestral history for some studies was ambiguous. We therefore tested whether the uncertainty in the ancestral order of these populations would affect the main findings. We selected studies for which we deemed the ancestry ambiguous; these were studies with an ancestral order (from ancestral to derived) based on south to north on the northern hemisphere, inland to coast, lower to higher altitude and few other reasons. We performed 1000 randomizations in which, we randomly picked for each comparison one of the populations as ancestral and the other as derived. We reran the analyses for the angle between plasticity and evolutionary divergence, plasticity and total divergence and the first eigenvector for the ancestral population in the derived environment (A in B) and evolutionary divergence. All findings were robust to the uncertainty of ancestral order since the point estimates produced by the randomized analyses fell within the confidence intervals of the main analyses (§8 and figure 11 in Appendix S2).

**References:**

Boshier, D., Broadhurst, L., Cornelius, J., Gallo, L., Koskela, J., Loo, J., *et al.* (2015). Is local best? Examining the evidence for local adaptation in trees and its scale. *Environ. Evid.*, 4, 1–10.

Halbritter, A.H., Fior, S., Keller, I., Billeter, R., Edwards, P.J., Holderegger, R., *et al.* (2018). Trait differentiation and adaptation of plants along elevation gradients. *J. Evol. Biol.*, 31, 784–800.

Hereford, J. (2009). A Quantitative Survey of Local Adaptation and Fitness Trade-Offs. *Am. Nat.*, 173, 579–588.

Leimu, R. & Fischer, M. (2008). A Meta-Analysis of Local Adaptation in Plants. *PLoS One*, 3.

Palacio-López, K., Beckage, B., Scheiner, S. & Molofsky, J. (2015). The ubiquity of phenotypic plasticity in plants: A synthesis. *Ecol. Evol.*, 5, 3389–3400.

**Table S1.1:** AICc values for the selection of random structure.

| | Error + study | Error + species | Error + study + species |
|---|---|---|---|
| Change in variation between AinA and AinB | **184.13** | **184.13** | 184.76 |
| Change in variation between AinA and BinB | **184.04** | 197.47 | 198.24 |
| Angle two plasticity A and plasticity B | **251.31** | **251.31** | 253.54 |
| Length difference in plasticity BtoA and AtoB | **151.75** | 151.93 | 153.97 |
| Angle plasticity of A and Pmax for AinA | **182.88** | 183.43 | 183.43 |
| Angle plasticity of A and total divergence of B | **256.66** | 263.33 | 258.89 |
| Proportion of total divergence due to plasticity | **129.41** | 143.07 | 131.64 |
| Angle between plasticity of A and evolutionary divergence of B | **271.13** | **271.13** | 273.36 |
| Angle evolutionary divergence of B and Pmax for AinB | **188.57** | **188.57** | 191.37 |
