# Supplementary material for: Plasticity leaves a phenotypic signature during local adaptation
Source: Evol Lett. 2020 Jun 9;4(4):360–70. doi: 10.1002/evl3.185 (PMC7403707; doi:10.1002/evl3.185)
Supplement: Supplementary file 2 — Appendix S2: Code and Analyses [file EVL3-4-360-s002.pdf]

# Appendix S2: Plasticity takes the lead in local adaptation

*Reinder Radersma, Daniel A. W. Noble & Tobias Uller*

*2020-05-06*

## Contents

**1 Introduction** **1**

**2 Preparation** **1**

**3 Discriptive statistics** **6**

**4 Plasticity and phenotypic variation in locally adapted populations** **8**
4.1 Change in variation between AinA and AinB . . . . . . . . . . 8
4.2 Change in variation between AinA and BinB . . . . . . . . . . 9
4.3 Change in the shape of P-matrix . . . . . . . . . . 10
4.4 Angle between plasticity A and plasticity B . . . . . . . . . . 10
4.5 Length difference in plasticity BtoA and AtoB . . . . . . . . . . 13
4.6 Angle plasticity of A and Pmax for AinA . . . . . . . . . . 15

**5 Alignment between plasticity and locally adapted phenotypes** **17**
5.1 Angle plasticity of A and total divergence of B . . . . . . . . . . 17
5.2 Proportion of total divergence due to plasticity . . . . . . . . . . 20

**6 Alignment between plasticity and evolutionary divergence** **22**
6.1 Angle plasticity of A and evolutionary divergence of B . . . . . . . . . . 22
6.2 Correlation between angle between evolutionary divergence and plasticity and the contribution of plasticity to total divergence. . . . . . . . . . . 24
6.3 Angle evolutionary divergence of B and Pmax for AinB . . . . . . . . . . 24

**7 Produce polar plots** **28**

**8 Ambiguous ancestry** **31**

**9 Check for the seriousness of non-positive definite variance-covariance matrices** **35**

## 1 Introduction

This document is an electronic supplement to:

> Reinder Radersma, Daniel A. W. Noble & Tobias Uller (2020) Plasticity takes the lead in local adaptation. Submitted to *Evolution Letters*.

It contains the code, additional graphs and more details of the analyses presented in the manuscript. This code can be used under the Creative Commons licence BY-NC-SA 4.0 https://creativecommons.org/licenses/by-nc-sa/4.0/.

## 2 Preparation

Set working directory and load libraries and functions.

```r
rm(list = ls(all = TRUE))
setwd("~/reciprocal-transplants")

# libraries
library(metafor)
```

```
## Loading required package: Matrix

## Loading 'metafor' package (version 2.0-0). For an overview
## and introduction to the package please type: help(metafor).
```

```r
library(devtools)
```

```
## Warning: package 'devtools' was built under R version 3.5.2

## Loading required package: usethis

## Warning: package 'usethis' was built under R version 3.5.2
```

```r
library(ape)
library(phytools)
```

```
## Warning: package 'phytools' was built under R version 3.5.2

## Loading required package: maps
```

```r
library(metaAidR)
library(plotrix)
```

```
## Warning: package 'plotrix' was built under R version 3.5.2
```

```r
# Needed for creating vcv matrix; check ?make_VCV_matrix for more details
devtools::install_github("daniel1noble/metaAidR")
```

```
## Skipping install of 'metaAidR' from a github remote, the SHA1 (cacb5797) has not changed since last [illegible]
##   Use `force = TRUE` to force installation
```

```r
# functions
source("ma-retr-analysis-functions.R")
```

Load datasets

```r
# load the data frames which were produced in the previous step
load(file="ma-retr-data_frames.Rdata")

# remove letter indicating substudies to treat them as one and the same study in further analyses.
# Keep substudy id's for the production of variance-covariance matrices.
ma1$substudy.id <- ma1$study.id
ma2$substudy.id <- ma2$study.id
ma1$study.id <- substr(ma1$study.id,1,5)
ma2$study.id <- substr(ma2$study.id,1,5)

names(ma1)
```

```
##  [1] "study.id"             "comp.id"              "n.traits"
##  [4] "morph.traits"         "env.anc"              "env.nov"
##  [7] "citation"             "species"              "taxon"
## [10] "length_PVa_mean"      "length_PVa_sd"        "length_PVb_mean"
## [13] "length_PVb_sd"        "length_delta_PV_mean" "length_delta_PV_sd"
## [16] "length_EDa_mean"      "length_EDa_sd"        "length_EDb_mean"
```

```
## [19] "length_EDb_sd"        "length_AtoB_mean"     "length_AtoB_sd"
## [22] "length_BtoA_mean"     "length_BtoA_sd"       "length_TDb_mean"
## [25] "length_TDb_sd"        "relmean_AinB_mean"    "relv_AinB_sd"
## [28] "relmean_BinA_mean"    "relmean_BinA_sd"      "relmean_BinB_mean"
## [31] "relmean_BinB_sd"      "relsd_AinB_mean"      "relsd_AinB_sd"
## [34] "relsd_BinA_mean"      "relsd_BinA_sd"        "relsd_BinB_mean"
## [37] "relsd_BinB_sd"        "angle_PVa_TDb_mean"   "angle_PVa_TDb_sd"
## [40] "angle_PVa_EDb_mean"   "angle_PVa_EDb_sd"     "angle90_PVa_EDb_mean"
## [43] "angle90_PVa_EDb_sd"   "angle_PVa_PVb_mean"   "angle_PVa_PVb_sd"
## [46] "PVa_on_TDb_mean"      "PVa_on_TDb_sd"        "angle_PVb_EDa_mean"
## [49] "angle_PVb_EDa_sd"     "angle_PVb_TDa_mean"   "angle_PVb_TDa_sd"
## [52] "fitness"              "timing.traits"        "substudy.id"
```

```
names(ma2)
```

```
##  [1] "study.id"                 "comp.id"
##  [3] "n.traits"                 "morph.traits"
##  [5] "env.anc"                  "env.nov"
##  [7] "citation"                 "species"
##  [9] "taxon"                    "angle_Paa_EDb_mean"
## [11] "angle_Paa_EDb_sd"         "angle_Pab_EDb_mean"
## [13] "angle_Pab_EDb_sd"         "angle_Paa_PVa_mean"
## [15] "angle_Paa_PVa_sd"         "angle_Pbb_EDb_mean"
## [17] "angle_Pbb_EDb_sd"         "angle_Paa_Pbb_mean"
## [19] "angle_Paa_Pbb_sd"         "angle_Paa_Pab_mean"
## [21] "angle_Paa_Pab_sd"         "angle_Pab_Pbb_mean"
## [23] "angle_Pab_Pbb_sd"         "angle_Pba_EDa_mean"
## [25] "angle_Pba_EDa_sd"         "delta_PVaEDb_PaaEDb_mean"
## [27] "delta_PVaEDb_PaaEDb_sd"   "delta_PVaEDb_PabEDb_mean"
## [29] "delta_PVaEDb_PabEDb_sd"   "shape_Paa_mean"
## [31] "shape_Paa_sd"             "shape_Pba_mean"
## [33] "shape_Pba_sd"             "shape_Pab_mean"
## [35] "shape_Pab_sd"             "shape_Pbb_mean"
## [37] "shape_Pbb_sd"             "det_AinB_mean"
## [39] "det_AinB_sd"              "det_BinB_mean"
## [41] "det_BinB_sd"              "fitness"
## [43] "timing.traits"            "substudy.id"
```

```
# discrete classes
# ma1$timing.traits <- round(ma1$timing.traits,0)
# ma2$timing.traits <- round(ma2$timing.traits,0)
```

Remove studies which are not on plants.

```
# the data for all taxa is stored in ma3 for descriptive statistics on the different taxa.
ma3 <- ma1
ma1 <- ma1[which(ma1$taxon == "plant"),]
ma2 <- ma2[which(ma2$taxon == "plant"),]
```

Update taxon names to make sure the names in the datasets match the names in the pylogenetic tree

```
# dataset 1
original_names_ma1 <- c("Diodia_teres", "Thlaspi_caerulescens", "Mimulus_guttatus")
new_names_ma1 <- c("Diodella_teres","Noccaea_caerulescens", "Erythranthe_guttata")
ma1$spp <- fix.taxon.names(ma1$species, original_names_ma1, new_names_ma1)

# dataset 2
```

```
original_names_ma2 <- c("Thlaspi_caerulescens","Diodia_teres")
new_names_ma2 <- c("Noccaea_caerulescens","Diodella_teres")
ma2$spp <- fix.taxon.names(ma2$species, original_names_ma2, new_names_ma2)
```

Check the overlap between studies and species

```
# Studies per species in dataset 1
apply(!is.na(tapply(ma1$study.id,list(ma1$study.id,ma1$spp),length)),1,sum)
```

```
## DD182 DD184 DD185 HA035 HE003 HE005 HE006 HE008 HE009 HE011 HE014 PL017
##     1     1     1     1     1     1     1     1     1     1     1     1
## SR048 SR058 SR062 SR068 SR069 SR076 SR082 SR084 SR085 SR088 SR096 SR097
##     1     3     1     1     1     1     1     1     1     1     1     1
## SR098 SR101 SR109 SR115 SR116 SR128 SR164 SR167 SR168 SR188
##     1     1     1     1     1     1     1     1     1     1
```

```
# Species per study in dataset 1
apply(!is.na(tapply(ma1$study.id,list(ma1$study.id,ma1$spp),length)),2,sum)
```

```
##      Abies_sachalinensis     Abutilon_theophrasti             Acer_negundo
##                        1                        1                        1
##  Ambrosia_artemisiifolia       Arabidopsis_lyrata     Arabidopsis_thaliana
##                        1                        3                        1
##    Arrhenatherum_elatius     Cardamine_cordifolia         Carex_aquatilis
##                        1                        1                        1
##          Centaurea_jacea Chamaecrista_fasciculata        Clarkia_xantiana
##                        1                        1                        1
##    Collinsia_sparsiflora            Crepis_sancta           Diodella_teres
##                        1                        1                        1
##      Erythranthe_guttata            Festuca_eskia          Gilia_capitata
##                        1                        1                        1
##     Heliosperma_pusillum  Hydrocotyle_bonariensis     Hypochaeris_radicata
##                        1                        1                        1
##       Impatiens_capensis     Noccaea_caerulescens        Pinus_densiflora
##                        1                        1                        1
##          Pinus_ponderosa           Plantago_major              Poa_alpina
##                        1                        1                        1
##            Poa_pratensis        Prunella_vulgaris        Rhinanthus_minor
##                        1                        1                        1
##     Saussurea_nigrescens    Senecio_pinnatifolius            Viola_biflora
##                        1                        1                        1
##      Xanthium_strumarium
##                        1
```

```
# Studies per species in dataset 2
apply(!is.na(tapply(ma2$study.id,list(ma2$study.id,ma2$spp),length)),1,sum)
```

```
## DD182 DD184 DD185 HA035 HE006 SR048 SR058 SR062 SR068 SR069 SR085 SR088
##     1     1     1     1     1     1     3     1     1     1     1     1
## SR096 SR097 SR098 SR101 SR109 SR115 SR116 SR128 SR164 SR188
##     1     1     1     1     1     1     1     1     1     1
```

```
# Species per study in dataset 2
apply(!is.na(tapply(ma2$study.id,list(ma2$study.id,ma2$spp),length)),2,sum)
```

```
##      Abies_sachalinensis  Ambrosia_artemisiifolia       Arabidopsis_lyrata
##                        1                        1                        3
```

```
##     Arabidopsis_thaliana    Arrhenatherum_elatius     Cardamine_cordifolia
##                        1                        1                        1
##          Centaurea_jacea Chamaecrista_fasciculata         Clarkia_xantiana
##                        1                        1                        1
##            Crepis_sancta           Diodella_teres     Heliosperma_pusillum
##                        1                        1                        1
##     Hypochaeris_radicata     Noccaea_caerulescens        Pinus_densiflora
##                        1                        1                        1
##           Plantago_major               Poa_alpina            Poa_pratensis
##                        1                        1                        1
##         Rhinanthus_minor     Saussurea_nigrescens    Senecio_pinnatifolius
##                        1                        1                        1
##            Viola_biflora
##                        1
```

Generally there is one unique species per study, but there is one species occuring in 3 studies and one study investigating 3 species.

Load the phylogenetic trees and transform them to variance-covariance matrices.

```
phylos <- readRDS("phylo_tree_list")
# dataset 1
A_ma1 <- vcv(phylos$phylo_3_ma1, corr = TRUE)
# dataset 2
A_ma2 <- vcv(phylos$phylo_3_ma2, corr = TRUE)
```

Add observation identities, which will later be used in the meta-analytic models

```
ma1$err <- 1:dim(ma1)[1]
ma2$err <- 1:dim(ma2)[1]
```

Add the sources on which the ancestry order is based.

```
# add sources of fitness info
ma1$anc.cat <- NA
ma2$anc.cat <- NA

# most south on northern hemisphere
fitinfo <- c("HE003","HE006","SR058","SR088","SR116","SR121","SR128","SR155")
ma1$anc.cat[ma1$study.id %in% fitinfo] <- "SN"
ma2$anc.cat[ma2$study.id %in% fitinfo] <- "SN"

# higest fitness in own environment / most benign environment
fitinfo <- c("HE005","HE009","SR068","SR096","SR157","SR167","SR168")
ma1$anc.cat[ma1$study.id %in% fitinfo] <- "HF"
ma2$anc.cat[ma2$study.id %in% fitinfo] <- "HF"

# geographic distribution/ more central in geographic range
fitinfo <- c("HE008","HA035","SR084","SR115","DD184")
ma1$anc.cat[ma1$study.id %in% fitinfo] <- "GD"
ma2$anc.cat[ma2$study.id %in% fitinfo] <- "GD"

# other reasons
fitinfo <- c("HE011","SR100","DD185")
ma1$anc.cat[ma1$study.id %in% fitinfo] <- "OT"
ma2$anc.cat[ma2$study.id %in% fitinfo] <- "OT"
```

```r
# most natural environment
fitinfo <- c("HE014","SR069","SR085","SR097","SR109")
ma1$anc.cat[ma1$study.id %in% fitinfo] <- "MN"
ma2$anc.cat[ma2$study.id %in% fitinfo] <- "MN"

# indicated by the authors, mostly reffering to other studies
fitinfo <- c("PL017","SR048","SR060","SR164")
ma1$anc.cat[ma1$study.id %in% fitinfo] <- "AU"
ma2$anc.cat[ma2$study.id %in% fitinfo] <- "AU"

# historical reconds of invasion
fitinfo <- c("PL023","SR076","SR082","SR117","DD183")
ma1$anc.cat[ma1$study.id %in% fitinfo] <- "HR"
ma2$anc.cat[ma2$study.id %in% fitinfo] <- "HR"

# most inland populations (as opposed to coastal)
fitinfo <- c("SR062","SR095","SR101","SR107")
ma1$anc.cat[ma1$study.id %in% fitinfo] <- "IC"
ma2$anc.cat[ma2$study.id %in% fitinfo] <- "IC"

# lowest altitude
fitinfo <- c("SR098","DD182")
ma1$anc.cat[ma1$study.id %in% fitinfo] <- "AL"
ma2$anc.cat[ma2$study.id %in% fitinfo] <- "AL"
```

# 3 Discriptive statistics

```r
# total number of studies
length(unique(ma1$study.id))
```

```
## [1] 34
```

```r
# number of species in dataset 1
length(unique(ma1$species))
```

```
## [1] 34
```

```r
# number of species in dataset 2
length(unique(ma2$species))
```

```
## [1] 22
```

```r
# number of studies per taxon
tapply(ma3$taxon[!duplicated(ma3$study.id)],ma3$taxon[!duplicated(ma3$study.id)],length)
```

```
##     frog mollusc   plant
##        1       2      34
```

```r
# minimum number of traits
min(ma1$n.traits[which(!duplicated(ma1$study.id))])
```

```
## [1] 2
```

```r
# maximum number of traits
max(ma1$n.traits[which(!duplicated(ma1$study.id))])
```

```
## [1] 9
```

```r
# mean number of traits
mean(ma1$n.traits[which(!duplicated(ma1$study.id))])
```

```
## [1] 3.823529
```

```r
# SD number of traits
sd(ma1$n.traits[which(!duplicated(ma1$study.id))])
```

```
## [1] 1.914389
```

```r
# number of observations for number of traits
length(ma1$n.traits[which(!duplicated(ma1$study.id))])
```

```
## [1] 34
```

```r
# number of studies with morphological traits only
sum(ma1$morph.traits[which(!duplicated(ma1$study.id))]==1)
```

```
## [1] 22
```

```r
# number of studies with phenology traits only
sum(ma1$morph.traits[which(!duplicated(ma1$study.id))]==0)
```

```
## [1] 3
```

```r
# number of studies with mix of morphological and phenology traits
sum(ma1$morph.traits[which(!duplicated(ma1$study.id))]<1 &
      ma1$morph.traits[which(!duplicated(ma1$study.id))]>0)
```

```
## [1] 9
```

```r
# number of comparisons per study
range(tapply(ma1$study.id,ma1$study.id,length))
```

```
## [1]  1 15
```

```r
#number of studies with only one comparison
sum(tapply(ma1$study.id,ma1$study.id,length)==1)
```

```
## [1] 23
```

```r
# plot local adaptation estimates to check for outliers

par(mfrow=c(1,2))
hist(ma1$fitness,xlim=c(-3,3),col="#5D9CEC",main="Dataset 1",xlab="Local adaptation")
hist(ma2$fitness,xlim=c(-3,3),col="#5D9CEC",main="Dataset 2",xlab="Local adaptation")
```

All meta-analytic analyses will have the following structure: first variance-covariances matrices are produced, which account for the fact that different comparisons within one study are partly based on the same data (if they share one environment rho = 0.5, otherwise 0). the AICc of models with 3 different random structures are compared to find the best fitting random stucture (study.id, species or study.id and species). Best model is used for meta-analytic estimates and when appropreate a model with covariates is also tested. First we tested relative changes in variation between experimental units.

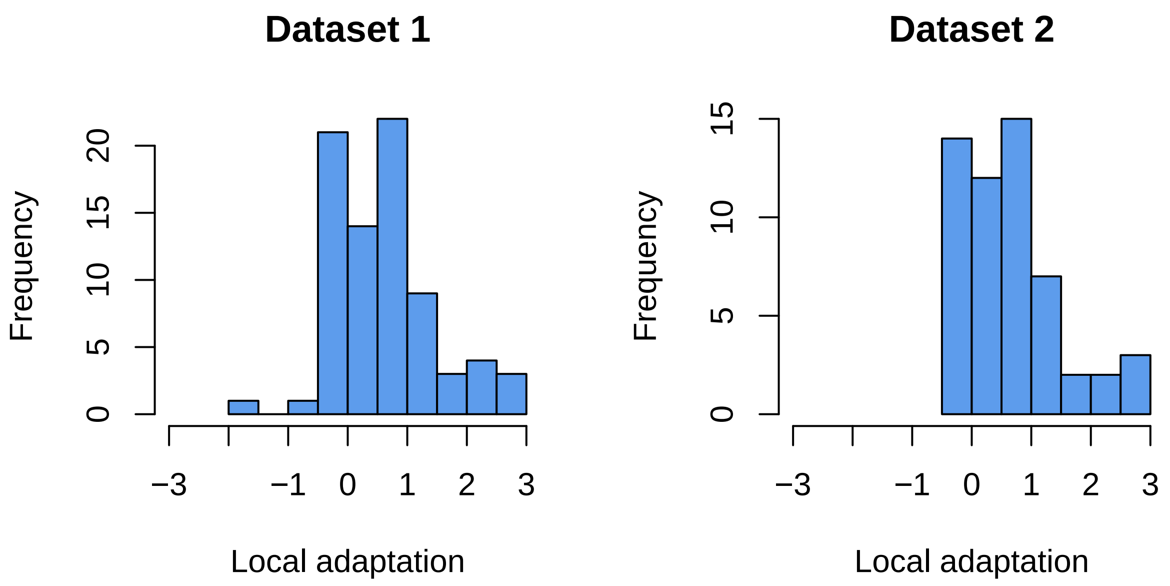


Figure 1: Histograms of local adaptation estimates

# 4 Plasticity and phenotypic variation in locally adapted populations

## 4.1 Change in variation between AinA and AinB

```
# Create VCV sampling variance matrix
V1 <- create.VCV.matrix(ma1$relsd_AinB_sd,ma1$env.anc,ma1$env.nov,ma1$substudy.id)

# intercept models
fit1.0.1 <- rma.mv(relsd_AinB_mean ~ 1, V = V1, data=ma1,
                   random = list(~1|study.id, ~1|err))
```

```
## Warning in rma.mv(relsd_AinB_mean ~ 1, V = V1, data = ma1, random = list(~1
## | : 'V' appears to be not positive definite.
```

```
fit1.0.2 <- rma.mv(relsd_AinB_mean ~ 1, V = V1, data=ma1,
                   random = list(~1|spp, ~1|err), R = list(spp = A_ma1))
```

```
## Warning in rma.mv(relsd_AinB_mean ~ 1, V = V1, data = ma1, random = list(~1
## | : 'V' appears to be not positive definite.
```

```
fit1.0.3 <- rma.mv(relsd_AinB_mean ~ 1, V = V1, data=ma1,
                   random = list(~1|study.id,~1|spp, ~1|err), R = list(spp = A_ma1))
```

```
## Warning in rma.mv(relsd_AinB_mean ~ 1, V = V1, data = ma1, random = list(~1
## | : 'V' appears to be not positive definite.
```

```
# compare AICc
round(fitstats(fit1.0.1,fit1.0.2,fit1.0.3),2)
```

```
##            fit1.0.1 fit1.0.2 fit1.0.3
## logLik:      -88.90   -88.90   -88.10
## deviance:    177.81   177.81   176.21
## AIC:         183.81   183.81   184.21
## BIC:         190.84   190.84   193.58
```

```
## AICc:       184.13   184.13   184.76
```

```r
# print best model
print(fit1.0.1)
```

```
##
## Multivariate Meta-Analysis Model (k = 78; method: REML)
##
## Variance Components:
##
##               estim    sqrt  nlvls  fixed    factor
## sigma^2.1  0.0000  0.0007     34     no  study.id
## sigma^2.2  0.2733  0.5228     78     no       err
##
## Model Results:
##
## estimate      se    zval    pval    ci.lb   ci.ub
##   0.1103  0.0635  1.7381  0.0822  -0.0141  0.2346  .
##
## ---
## Signif. codes:  0 '***' 0.001 '**' 0.01 '*' 0.05 '.' 0.1 ' ' 1
```

### 4.2 Change in variation between AinA and BinB

```r
# Create VCV sampling variance matrix
V2 <- create.VCV.matrix(ma1$relsd_BinB_sd,ma1$env.anc,ma1$env.nov,ma1$substudy.id)

# intercept models
fit2.0.1 <- rma.mv(relsd_BinB_mean ~ 1, V = V2, data=ma1,
                   random = list(~1|study.id, ~1|err))
```

```
## Warning in rma.mv(relsd_BinB_mean ~ 1, V = V2, data = ma1, random = list(~1
## | : 'V' appears to be not positive definite.
```

```r
fit2.0.2 <- rma.mv(relsd_BinB_mean ~ 1, V = V2, data=ma1,
                   random = list(~1|spp, ~1|err), R = list(spp = A_ma1))
```

```
## Warning in rma.mv(relsd_BinB_mean ~ 1, V = V2, data = ma1, random = list(~1
## | : 'V' appears to be not positive definite.
```

```r
fit2.0.3 <- rma.mv(relsd_BinB_mean ~ 1, V = V2, data=ma1,
                   random = list(~1|study.id,~1|spp, ~1|err), R = list(spp = A_ma1))
```

```
## Warning in rma.mv(relsd_BinB_mean ~ 1, V = V2, data = ma1, random = list(~1
## | : 'V' appears to be not positive definite.
```

```r
# compare AICc
round(fitstats(fit2.0.1,fit2.0.2,fit2.0.3),2)
```

```
##           fit2.0.1 fit2.0.2 fit2.0.3
## logLik:     -88.85   -95.57   -94.84
## deviance:   177.71   191.14   189.68
## AIC:        183.71   197.14   197.68
## BIC:        190.74   204.17   207.06
## AICc:       184.04   197.47   198.24
```

```r
# print best model
print(fit2.0.1)
```

```
##
## Multivariate Meta-Analysis Model (k = 78; method: REML)
##
## Variance Components:
##
##              estim    sqrt  nlvls  fixed    factor
## sigma^2.1   0.6485  0.8053     34     no  study.id
## sigma^2.2   0.2143  0.4630     78     no       err
##
## Model Results:
##
## estimate      se    zval    pval    ci.lb   ci.ub
##   0.3038  0.1560  1.9476  0.0515  -0.0019  0.6095  .
##
## ---
## Signif. codes:  0 '***' 0.001 '**' 0.01 '*' 0.05 '.' 0.1 ' ' 1
```

## 4.3 Change in the shape of P-matrix

The shape of the P-matrix is the length of the second eigenvector relative to the length of the first eigenvector. Estimates for the correlations between the shapes of the P-matrix in different experimental units are produced and stored in the simulation proccess and used to estimate means and confidence intervals

```r
# Mean change
colMeans(Pmax_corrs)
```

```
##   Paa_Pab   Pbb_Pba   Paa_Pbb
## 0.6133380 0.6196104 0.4989381
```

```r
# Condfidence intervals
apply(Pmax_corrs,2,quantile,probs=c(0.025,0.975))
```

```
##         Paa_Pab   Pbb_Pba   Paa_Pbb
## 2.5%  0.4745629 0.4877274 0.3432317
## 97.5% 0.7284142 0.7346164 0.6273933
```

```r
# plot
par(mfrow=c(1,3))
hist(Pmax_corrs[,1],col="#5D9CEC",xlab="P_AinA - P_AinB",main="")
hist(Pmax_corrs[,2],col="#5D9CEC",xlab="P_BinA - P_BinA",main="")
hist(Pmax_corrs[,3],col="#5D9CEC",xlab="P_AinA - P_BinB",main="")
```

## 4.4 Angle between plasticity A and plasticity B

```r
# test for uniform distribution
ks.test(logit(ma1$angle_PVa_PVb_mean,inv=TRUE),"punif")
```

```
##
##  One-sample Kolmogorov-Smirnov test
##
## data:  logit(ma1$angle_PVa_PVb_mean, inv = TRUE)
## D = 0.54976, p-value = 3.331e-16
## alternative hypothesis: two-sided
```

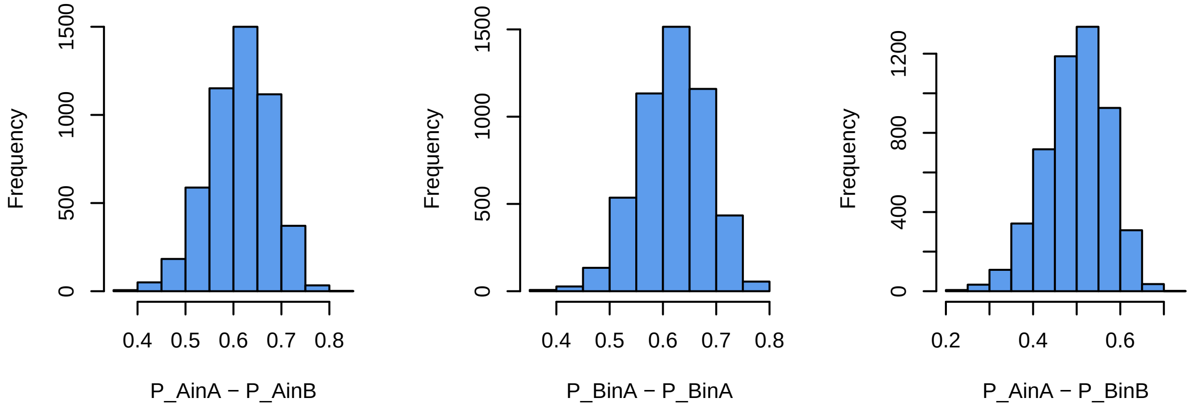


Figure 2: Correlations between shape of P-matrices

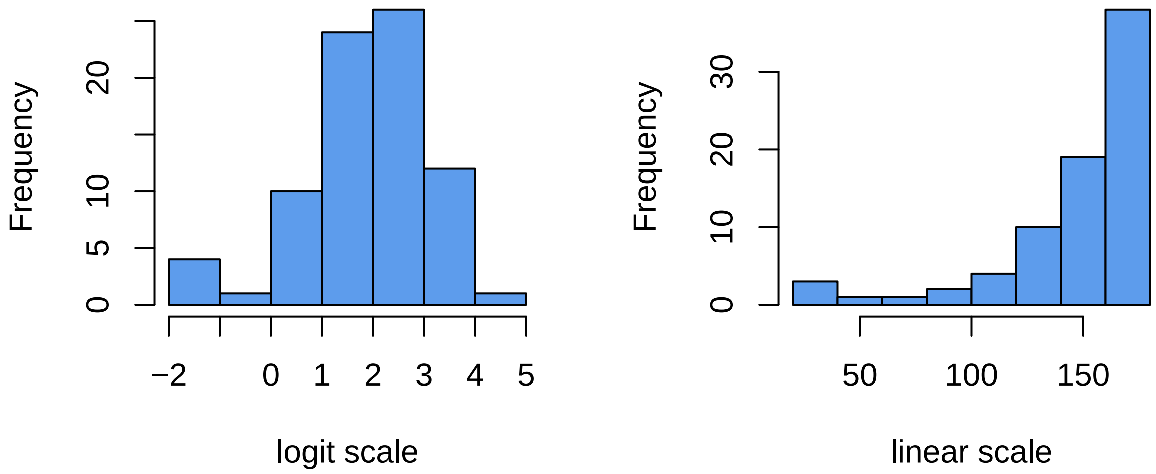


Figure 3: Angle between plasticity A and plasticity B

```
par(mfrow=c(1,2))
hist(ma1$angle_PVa_PVb_mean,col="#5D9CEC",xlab="logit scale",main="")
hist(logit(ma1$angle_PVa_PVb_mean,inv=TRUE)*180,col="#5D9CEC", xlab="linear scale",main="")
```

```
# Create VCV sampling variance matrix
V9 <- create.VCV.matrix(ma1$angle_PVa_PVb_sd,ma1$env.anc,ma1$env.nov,ma1$substudy.id)

# fit model
fit9.0.1 <- rma.mv(angle_PVa_PVb_mean ~ 1, V = V9, data=ma1,
                   random = list(~1|study.id, ~1|err))
```

```
## Warning in rma.mv(angle_PVa_PVb_mean ~ 1, V = V9, data = ma1, random =
## list(~1 | : 'V' appears to be not positive definite.
```

```
fit9.0.2 <- rma.mv(angle_PVa_PVb_mean ~ 1, V = V9, data=ma1,
                   random = list(~1|spp, ~1|err), R = list(spp = A_ma1))
```

```
## Warning in rma.mv(angle_PVa_PVb_mean ~ 1, V = V9, data = ma1, random =
## list(~1 | : 'V' appears to be not positive definite.
```

```r
fit9.0.3 <- rma.mv(angle_PVa_PVb_mean ~ 1, V = V9, data=ma1,
                   random = list(~1|study.id, ~1|spp, ~1|err), R = list(spp = A_ma1))
```

```
## Warning in rma.mv(angle_PVa_PVb_mean ~ 1, V = V9, data = ma1, random =
## list(~1 | : 'V' appears to be not positive definite.
```

```r
round(fitstats(fit9.0.1,fit9.0.2,fit9.0.3),2)
```

```
##           fit9.0.1 fit9.0.2 fit9.0.3
## logLik:    -122.49  -122.49  -122.49
## deviance:   244.99   244.99   244.99
## AIC:        250.99   250.99   252.99
## BIC:        258.02   258.02   262.36
## AICc:       251.31   251.31   253.54
```

```r
print(fit9.0.1)
```

```
##
## Multivariate Meta-Analysis Model (k = 78; method: REML)
##
## Variance Components:
##
##             estim    sqrt  nlvls  fixed    factor
## sigma^2.1  0.0000  0.0001     34     no  study.id
## sigma^2.2  1.0402  1.0199     78     no       err
##
## Model Results:
##
## estimate      se     zval    pval   ci.lb   ci.ub
##   1.7073  0.1428  11.9526  <.0001  1.4273  1.9873  ***
##
## ---
## Signif. codes:  0 '***' 0.001 '**' 0.01 '*' 0.05 '.' 0.1 ' ' 1
```

```r
print.to.angle(fit9.0.1, max.angle = 180)
```

```
## [1] "Angle: 152.37 - CI: 145.17 to 158.3"
## [1] "Intercept minus one unit:"
## numeric(0)
## [1] "Intercept plus one unit:"
## numeric(0)
```

```r
# add covariates
fit9.1.1 <- rma.mv(angle_PVa_PVb_mean ~ scale(n.traits) + fitness + timing.traits,
                   V = V9, data=ma1, random = list(~1|study.id, ~1|err))
```

```
## Warning in rma.mv(angle_PVa_PVb_mean ~ scale(n.traits) + fitness +
## timing.traits, : 'V' appears to be not positive definite.
```

```r
print(fit9.1.1)
```

```
##
## Multivariate Meta-Analysis Model (k = 78; method: REML)
##
## Variance Components:
##
##             estim    sqrt  nlvls  fixed    factor
```

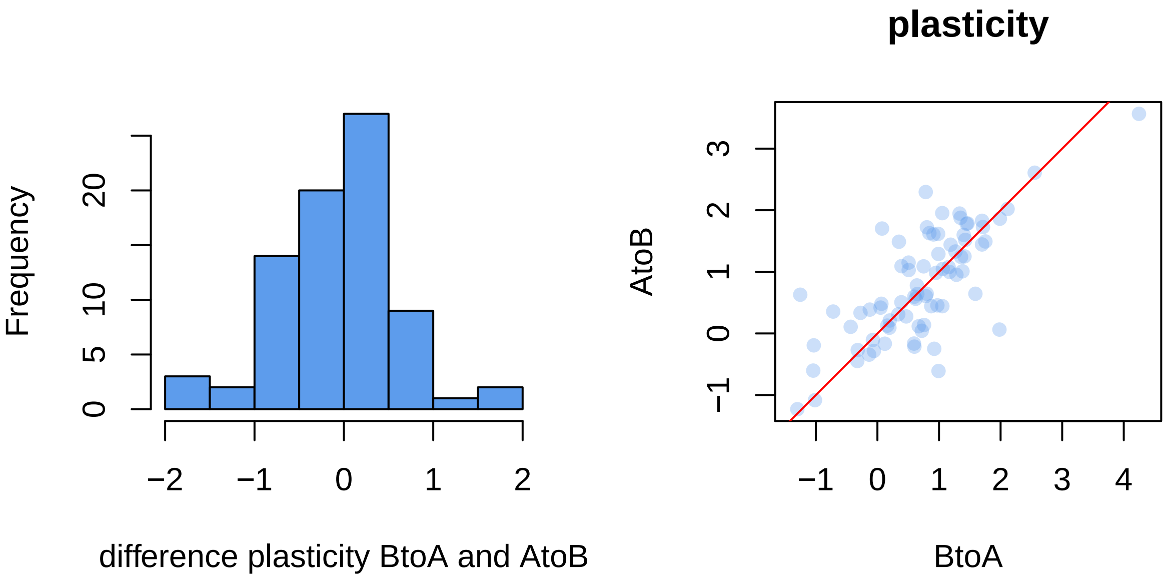


Figure 4: Length difference in plasticity BtoA and AtoB

```
## sigma^2.1  0.0000  0.0002     34     no  study.id
## sigma^2.2  1.0608  1.0299     78     no       err
##
## Test of Moderators (coefficient(s) 2:4):
## QM(df = 3) = 3.2929, p-val = 0.3486
##
## Model Results:
##
##                    estimate      se    zval    pval    ci.lb   ci.ub
## intrcpt              1.5302  0.2165  7.0664  <.0001   1.1058  1.9546  ***
## scale(n.traits)      0.0089  0.1558  0.0571  0.9544  -0.2965  0.3143
## fitness              0.0195  0.1602  0.1215  0.9033  -0.2945  0.3335
## timing.traits        0.7668  0.4502  1.7034  0.0885  -0.1155  1.6492    .
##
## ---
## Signif. codes:  0 '***' 0.001 '**' 0.01 '*' 0.05 '.' 0.1 ' ' 1
```

```
print.to.angle(fit9.1.1, max.angle = 180)
```

```
## [1] "Angle: 147.97 - CI: 135.24 to 157.67"
## [1] "Intercept minus one unit:"
## scale(n.traits)         fitness   timing.traits
##          147.73          147.45          122.77
## [1] "Intercept plus one unit:"
## scale(n.traits)         fitness   timing.traits
##          148.20          148.48          163.55
```

### 4.5 Length difference in plasticity BtoA and AtoB

```
par(mfrow=c(1,2))
hist(ma1$length_delta_PV_mean,col="#5D9CEC",xlab="difference plasticity BtoA and AtoB",main="")
plot(ma1$length_PVb_mean,ma1$length_PVa_mean,xlab="BtoA",ylab="AtoB",main="plasticity",pch=16,col="#5D9
abline(a=0,b=1,col="red")
```

```r
# Create VCV sampling variance matrix
V10 <- create.VCV.matrix(ma1$length_delta_PV_sd,ma1$env.anc,ma1$env.nov,ma1$substudy.id)

# fit model
fit10.0.1 <- rma.mv(length_delta_PV_mean ~ 1, V = V10, data=ma1,
                    random = list(~1|study.id, ~1|err))
```

```
## Warning in rma.mv(length_delta_PV_mean ~ 1, V = V10, data = ma1, random =
## list(~1 | : 'V' appears to be not positive definite.
```

```r
fit10.0.2 <- rma.mv(length_delta_PV_mean ~ 1, V = V10, data=ma1,
                    random = list(~1|spp, ~1|err), R = list(spp = A_ma1))
```

```
## Warning in rma.mv(length_delta_PV_mean ~ 1, V = V10, data = ma1, random =
## list(~1 | : 'V' appears to be not positive definite.
```

```r
fit10.0.3 <- rma.mv(length_delta_PV_mean ~ 1, V = V10, data=ma1,
                    random = list(~1|study.id, ~1|spp, ~1|err), R = list(spp = A_ma1))
```

```
## Warning in rma.mv(length_delta_PV_mean ~ 1, V = V10, data = ma1, random =
## list(~1 | : 'V' appears to be not positive definite.
```

```r
round(fitstats(fit10.0.1,fit10.0.2,fit10.0.3),2)
```

```
##           fit10.0.1 fit10.0.2 fit10.0.3
## logLik:      -72.71    -72.80    -72.71
## deviance:    145.42    145.60    145.42
## AIC:         151.42    151.60    153.42
## BIC:         158.45    158.63    162.79
## AICc:        151.75    151.93    153.97
```

```r
print(fit10.0.1)
```

```
##
## Multivariate Meta-Analysis Model (k = 78; method: REML)
##
## Variance Components:
##
##              estim    sqrt  nlvls  fixed    factor
## sigma^2.1   0.0482  0.2195     34     no  study.id
## sigma^2.2   0.2915  0.5399     78     no       err
##
## Model Results:
##
## estimate      se     zval    pval    ci.lb   ci.ub
##  -0.0070  0.0844  -0.0830  0.9339  -0.1723  0.1583
##
## ---
## Signif. codes:  0 '***' 0.001 '**' 0.01 '*' 0.05 '.' 0.1 ' ' 1
```

```r
# add covariates
fit10.1.1 <- rma.mv(length_delta_PV_mean ~ scale(n.traits) + fitness + timing.traits,
                    V = V10, data=ma1, random = list(~1|study.id, ~1|err))
```

```
## Warning in rma.mv(length_delta_PV_mean ~ scale(n.traits) + fitness +
## timing.traits, : 'V' appears to be not positive definite.
```

```r
print(fit10.1.1)
```

```
##
## Multivariate Meta-Analysis Model (k = 78; method: REML)
##
## Variance Components:
##
##             estim    sqrt  nlvls  fixed    factor
## sigma^2.1  0.0772  0.2778     34     no  study.id
## sigma^2.2  0.2818  0.5308     78     no       err
##
## Test of Moderators (coefficient(s) 2:4):
## QM(df = 3) = 1.7127, p-val = 0.6341
##
## Model Results:
##
##                   estimate      se     zval    pval    ci.lb   ci.ub
## intrcpt            -0.1062  0.1247  -0.8518  0.3943  -0.3506  0.1382
## scale(n.traits)     0.0507  0.1046   0.4847  0.6279  -0.1544  0.2558
## fitness             0.0980  0.0878   1.1156  0.2646  -0.0742  0.2701
## timing.traits       0.1958  0.2566   0.7628  0.4456  -0.3072  0.6987
##
## ---
## Signif. codes:  0 '***' 0.001 '**' 0.01 '*' 0.05 '.' 0.1 ' ' 1
```

### 4.6 Angle plasticity of A and Pmax for AinA

```r
# test for uniform distribution
ks.test(logit(ma2$angle_Paa_PVa_mean,inv=TRUE),"punif")
```

```
##
##  One-sample Kolmogorov-Smirnov test
##
## data:  logit(ma2$angle_Paa_PVa_mean, inv = TRUE)
## D = 0.36535, p-value = 4.346e-07
## alternative hypothesis: two-sided
```

```r
par(mfrow=c(1,2))
hist(ma2$angle_Paa_PVa_mean,col="#5D9CEC",xlab="logit scale",main="")
hist(logit(ma2$angle_Paa_PVa_mean,inv=TRUE)*90,col="#5D9CEC", xlab="linear scale",main="")
```

```r
# Create VCV sampling variance matrix
V8 <- create.VCV.matrix(ma2$angle_Paa_PVa_sd,ma2$env.anc,ma2$env.nov,ma2$substudy.id)

# intercept only
fit8.0.1 <- rma.mv(angle_Paa_PVa_mean ~ 1, V =V8, data=ma2,
                   random = list(~1|study.id, ~1|err))
```

```
## Warning in rma.mv(angle_Paa_PVa_mean ~ 1, V = V8, data = ma2, random =
## list(~1 | : 'V' appears to be not positive definite.
```

```r
fit8.0.2 <- rma.mv(angle_Paa_PVa_mean ~ 1, V =V8, data=ma2,
                   random = list(~1|spp, ~1|err), R = list(spp = A_ma2))
```

```
## Warning in rma.mv(angle_Paa_PVa_mean ~ 1, V = V8, data = ma2, random =
```

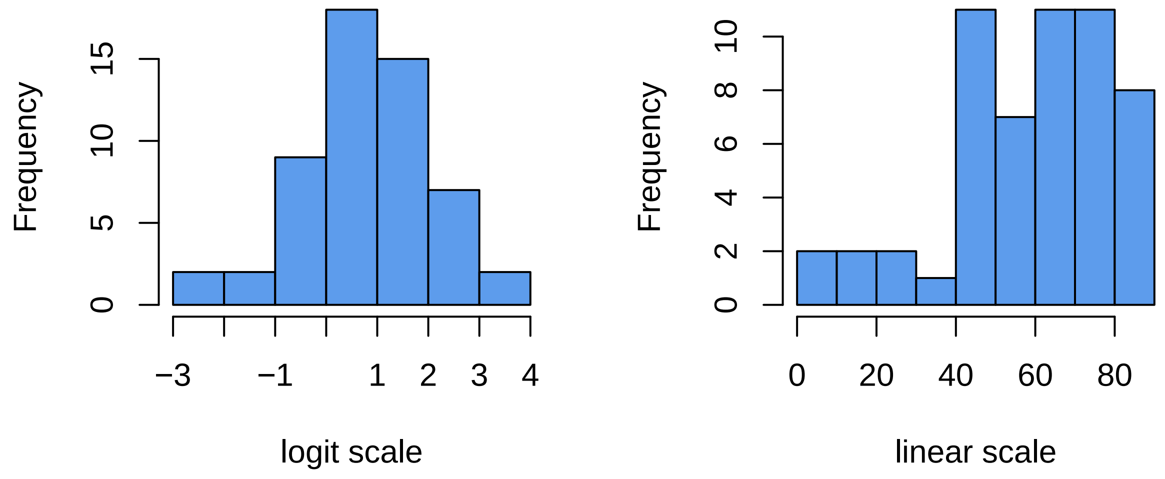


Figure 5: Angle plasticity of A and Pmax for AinA

```
## list(~1 | : 'V' appears to be not positive definite.
```

```r
fit8.0.3 <- rma.mv(angle_Paa_PVa_mean ~ 1, V =V8, data=ma2,
                   random = list(~1|study.id, ~1|spp, ~1|err), R = list(spp = A_ma2))
```

```
## Warning in rma.mv(angle_Paa_PVa_mean ~ 1, V = V8, data = ma2, random =
## list(~1 | : 'V' appears to be not positive definite.
```

```r
round(fitstats(fit8.0.1,fit8.0.2,fit8.0.3),2)
```

```
##           fit8.0.1 fit8.0.2 fit8.0.3
## logLik:     -88.20   -88.48   -88.20
## deviance:   176.40   176.95   176.40
## AIC:        182.40   182.95   184.40
## BIC:        188.36   188.92   192.35
## AICc:       182.88   183.43   185.21
```

```r
print(fit8.0.1)
```

```
##
## Multivariate Meta-Analysis Model (k = 55; method: REML)
##
## Variance Components:
##
##             estim    sqrt  nlvls  fixed    factor
## sigma^2.1  0.2177  0.4666     22     no  study.id
## sigma^2.2  0.8192  0.9051     55     no       err
##
## Model Results:
##
## estimate      se    zval    pval    ci.lb   ci.ub
##   0.4298  0.2213  1.9417  0.0522  -0.0040  0.8636  .
##
## ---
## Signif. codes:  0 '***' 0.001 '**' 0.01 '*' 0.05 '.' 0.1 ' ' 1
```

```r
print.to.angle(fit8.0.1)
```

```
## [1] "Angle: 54.52 - CI: 44.91 to 63.31"
```

```
## [1] "Intercept minus one unit:"
## numeric(0)
## [1] "Intercept plus one unit:"
## numeric(0)
```

```
# add covariates
fit8.1.1 <- rma.mv(angle_Paa_PVa_mean ~ scale(n.traits) + fitness + timing.traits,
                   V = V8, data=ma2, random = list(~1|study.id, ~1|err))
```

```
## Warning in rma.mv(angle_Paa_PVa_mean ~ scale(n.traits) + fitness +
## timing.traits, : 'V' appears to be not positive definite.
```

```
print(fit8.1.1)
```

```
##
## Multivariate Meta-Analysis Model (k = 55; method: REML)
##
## Variance Components:
##
##             estim    sqrt  nlvls  fixed    factor
## sigma^2.1  0.0000  0.0001     22     no  study.id
## sigma^2.2  0.8750  0.9354     55     no       err
##
## Test of Moderators (coefficient(s) 2:4):
## QM(df = 3) = 7.6492, p-val = 0.0538
##
## Model Results:
##
##                  estimate      se    zval    pval    ci.lb   ci.ub
## intrcpt            0.1448  0.3234  0.4477  0.6544  -0.4891  0.7787
## scale(n.traits)    0.5048  0.2246  2.2475  0.0246   0.0646  0.9450  *
## fitness            0.3720  0.2250  1.6535  0.0982  -0.0690  0.8129  .
## timing.traits      0.3510  0.5556  0.6318  0.5275  -0.7379  1.4398
##
## ---
## Signif. codes:  0 '***' 0.001 '**' 0.01 '*' 0.05 '.' 0.1 ' ' 1
```

```
print.to.angle(fit8.1.1)
```

```
## [1] "Angle: 48.25 - CI: 34.21 to 61.69"
## [1] "Intercept minus one unit:"
## scale(n.traits)         fitness   timing.traits
##           36.99           39.91           40.38
## [1] "Intercept plus one unit:"
## scale(n.traits)         fitness   timing.traits
##           59.12           56.38           55.93
```

# 5 Alignment between plasticity and locally adapted phenotypes

## 5.1 Angle plasticity of A and total divergence of B

```
# test for uniform distribution
ks.test(logit(ma1$angle_PVa_TDb_mean,inv=TRUE),"punif")
```

```
##
##  One-sample Kolmogorov-Smirnov test
```

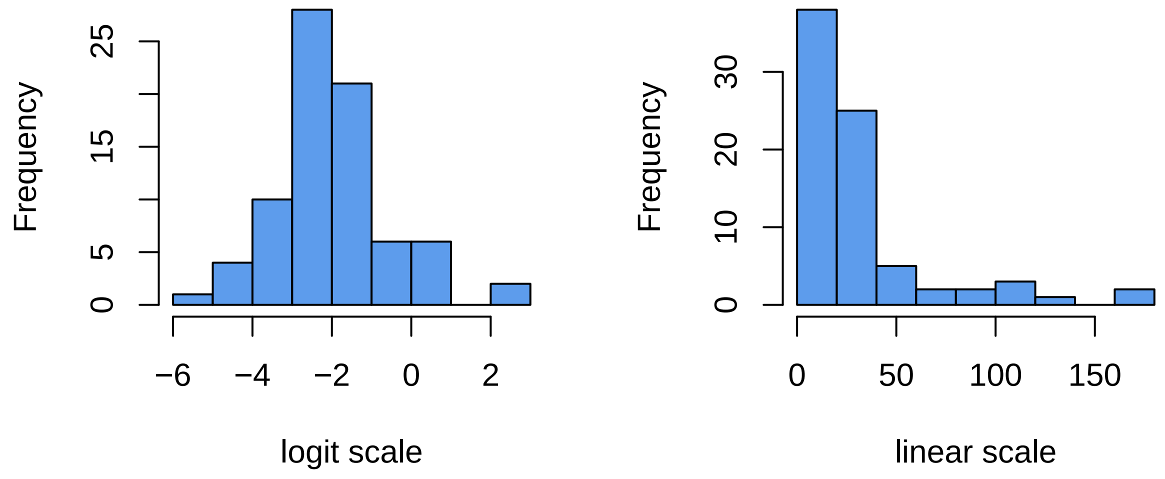


Figure 6: Angle plasticity of A and total divergence of B

```
##
## data:  logit(ma1$angle_PVa_TDb_mean, inv = TRUE)
## D = 0.59038, p-value = 3.331e-16
## alternative hypothesis: two-sided
```

```
par(mfrow=c(1,2))
hist(ma1$angle_PVa_TDb_mean,col="#5D9CEC",xlab="logit scale",main="")
hist(logit(ma1$angle_PVa_TDb_mean,inv=TRUE)*180,col="#5D9CEC", xlab="linear scale",main="")
```

```
# Create VCV sampling variance matrix
V3 <- create.VCV.matrix(ma1$angle_PVa_TDb_sd,ma1$env.anc,ma1$env.nov,ma1$substudy.id)

# intercept model
fit3.0.1 <- rma.mv(angle_PVa_TDb_mean ~ 1, V = V3, data=ma1,
                   random = list(~1|study.id, ~1|err))
```

```
## Warning in rma.mv(angle_PVa_TDb_mean ~ 1, V = V3, data = ma1, random =
## list(~1 | : 'V' appears to be not positive definite.
```

```
fit3.0.2 <- rma.mv(angle_PVa_TDb_mean ~ 1, V = V3, data=ma1,
                   random = list(~1|spp,~1|err), R = list(spp = A_ma1))
```

```
## Warning in rma.mv(angle_PVa_TDb_mean ~ 1, V = V3, data = ma1, random =
## list(~1 | : 'V' appears to be not positive definite.
```

```
fit3.0.3 <- rma.mv(angle_PVa_TDb_mean ~ 1, V = V3, data=ma1,
                   random = list(~1|study.id,~1|spp,~1|err), R = list(spp = A_ma1))
```

```
## Warning in rma.mv(angle_PVa_TDb_mean ~ 1, V = V3, data = ma1, random =
## list(~1 | : 'V' appears to be not positive definite.
```

```
round(fitstats(fit3.0.1,fit3.0.2,fit3.0.3),2)
```

```
##            fit3.0.1 fit3.0.2 fit3.0.3
## logLik:     -125.17  -128.50  -125.17
## deviance:    250.33   257.00   250.33
## AIC:         256.33   263.00   258.33
## BIC:         263.36   270.03   267.71
## AICc:        256.66   263.33   258.89
```

```r
print(fit3.0.1)
```

```
##
## Multivariate Meta-Analysis Model (k = 78; method: REML)
##
## Variance Components:
##
##              estim    sqrt  nlvls  fixed    factor
## sigma^2.1  0.6702  0.8187     34     no  study.id
## sigma^2.2  0.7351  0.8574     78     no       err
##
## Model Results:
##
## estimate      se     zval    pval    ci.lb    ci.ub
##  -1.8097  0.1979  -9.1454  <.0001  -2.1975  -1.4219  ***
##
## ---
## Signif. codes:  0 '***' 0.001 '**' 0.01 '*' 0.05 '.' 0.1 ' ' 1
```

```r
print.to.angle(fit3.0.1, max.angle = 180)
```

```
## [1] "Angle: 25.32 - CI: 17.99 to 34.99"
## [1] "Intercept minus one unit:"
## numeric(0)
## [1] "Intercept plus one unit:"
## numeric(0)
```

```r
# add covariates
fit3.1.1 <- rma.mv(angle_PVa_TDb_mean ~ scale(n.traits) + fitness + timing.traits,
                   V = V3, data=ma1, random = list(~1|study.id, ~1|err))
```

```
## Warning in rma.mv(angle_PVa_TDb_mean ~ scale(n.traits) + fitness +
## timing.traits, : 'V' appears to be not positive definite.
```

```r
print(fit3.1.1)
```

```
##
## Multivariate Meta-Analysis Model (k = 78; method: REML)
##
## Variance Components:
##
##              estim    sqrt  nlvls  fixed    factor
## sigma^2.1  0.0000  0.0000     34     no  study.id
## sigma^2.2  1.1794  1.0860     78     no       err
##
## Test of Moderators (coefficient(s) 2:4):
## QM(df = 3) = 4.0997, p-val = 0.2509
##
## Model Results:
##
##                  estimate      se     zval    pval    ci.lb    ci.ub
## intrcpt           -2.2253  0.2228  -9.9878  <.0001  -2.6620  -1.7886  ***
## scale(n.traits)    0.1982  0.1570   1.2621  0.2069  -0.1096   0.5059
## fitness            0.2729  0.1650   1.6540  0.0981  -0.0505   0.5963    .
## timing.traits      0.4516  0.4433   1.0187  0.3083  -0.4172   1.3204
##
```

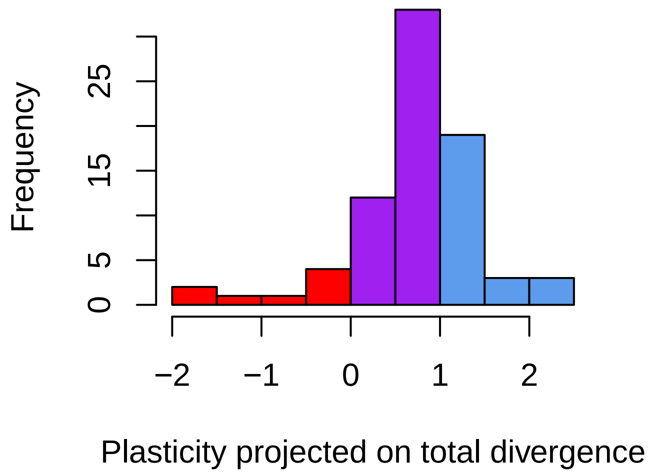


Figure 7: Proportion of total divergence due to plasticity. Studies with plasticity in the opposing direction are marked in red, studies with plasticity undershooting total divergence are in purple and studies with plasticity overshooting in blue.

```
## ---
## Signif. codes:  0 '***' 0.001 '**' 0.01 '*' 0.05 '.' 0.1 ' ' 1
```

## 5.2 Proportion of total divergence due to plasticity

```
hist(ma1$PVa_on_TDb_mean,col=c(rep("red",4),rep("purple",2),rep("#5D9CEC",3)),xlab="Plasticity projecte
```

```
# Create VCV sampling variance matrix
V5 <- create.VCV.matrix(ma1$PVa_on_TDb_sd,ma1$env.anc,ma1$env.nov,ma1$substudy.id)

# fit intercept model
fit5.0.1 <- rma.mv(PVa_on_TDb_mean ~ 1, V = V5, data=ma1,
                   random = list(~1|study.id, ~1|err))
```

```
## Warning in rma.mv(PVa_on_TDb_mean ~ 1, V = V5, data = ma1, random = list(~1
## | : 'V' appears to be not positive definite.
```

```
fit5.0.2 <- rma.mv(PVa_on_TDb_mean ~ 1, V = V5, data=ma1,
                   random = list(~1|spp, ~1|err), R = list(spp = A_ma1))
```

```
## Warning in rma.mv(PVa_on_TDb_mean ~ 1, V = V5, data = ma1, random = list(~1
## | : 'V' appears to be not positive definite.
```

```
fit5.0.3 <- rma.mv(PVa_on_TDb_mean ~ 1, V = V5, data=ma1,
                   random = list(~1|study.id, ~1|spp, ~1|err), R = list(spp = A_ma1))
```

```
## Warning in rma.mv(PVa_on_TDb_mean ~ 1, V = V5, data = ma1, random = list(~1
## | : 'V' appears to be not positive definite.
```

```
round(fitstats(fit5.0.1,fit5.0.2,fit5.0.3),2)
```

```
##           fit5.0.1 fit5.0.2 fit5.0.3
## logLik:     -61.54   -68.37   -61.54
## deviance:   123.08   136.74   123.08
## AIC:        129.08   142.74   131.08
## BIC:        136.11   149.78   140.46
```

```
## AICc:       129.41   143.07   131.64
```

```r
print(fit5.0.1)
```

```
##
## Multivariate Meta-Analysis Model (k = 78; method: REML)
##
## Variance Components:
##
##               estim    sqrt  nlvls  fixed    factor
## sigma^2.1   0.2013  0.4487     34     no  study.id
## sigma^2.2   0.1226  0.3502     78     no       err
##
## Model Results:
##
## estimate      se    zval    pval   ci.lb   ci.ub
##   0.7395  0.0954  7.7505  <.0001  0.5525  0.9265  ***
##
## ---
## Signif. codes:  0 '***' 0.001 '**' 0.01 '*' 0.05 '.' 0.1 ' ' 1
```

```r
# Add covariates
fit5.1.1 <- rma.mv(PVa_on_TDb_mean ~ scale(n.traits) + fitness + timing.traits,
                   V = V5, data=ma1, random = list(~1|study.id, ~1|err))
```

```
## Warning in rma.mv(PVa_on_TDb_mean ~ scale(n.traits) + fitness +
## timing.traits, : 'V' appears to be not positive definite.
```

```r
print(fit5.1.1)
```

```
##
## Multivariate Meta-Analysis Model (k = 78; method: REML)
##
## Variance Components:
##
##               estim    sqrt  nlvls  fixed    factor
## sigma^2.1   0.2045  0.4522     34     no  study.id
## sigma^2.2   0.1177  0.3431     78     no       err
##
## Test of Moderators (coefficient(s) 2:4):
## QM(df = 3) = 5.1588, p-val = 0.1605
##
## Model Results:
##
##                  estimate      se     zval    pval    ci.lb   ci.ub
## intrcpt            0.8745  0.1198   7.2969  <.0001   0.6396  1.1094  ***
## scale(n.traits)   -0.1482  0.1023  -1.4483  0.1475  -0.3487  0.0524
## fitness           -0.0962  0.0665  -1.4461  0.1482  -0.2266  0.0342
## timing.traits     -0.4321  0.2776  -1.5565  0.1196  -0.9761  0.1120
##
## ---
## Signif. codes:  0 '***' 0.001 '**' 0.01 '*' 0.05 '.' 0.1 ' ' 1
```

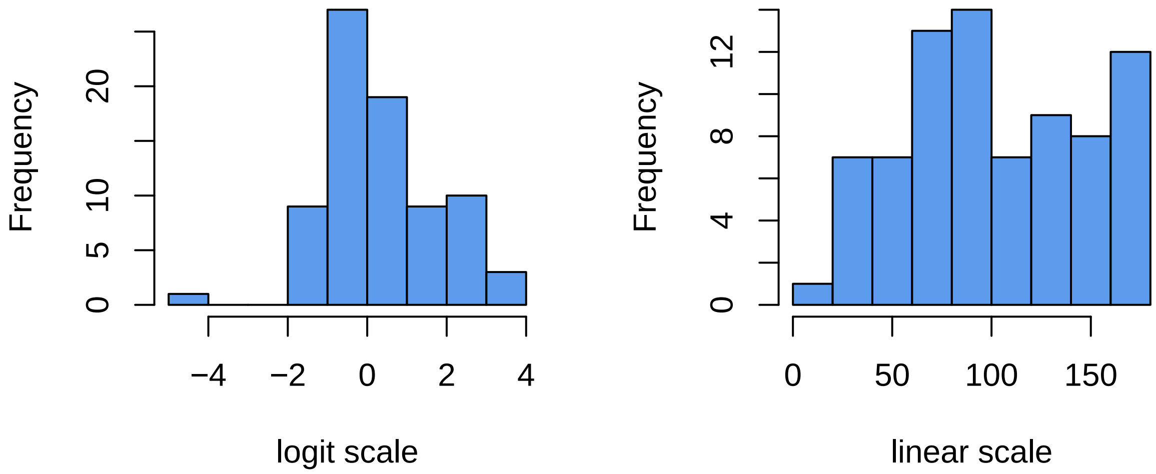


Figure 8: Angle plasticity of A and evolutionary divergence of B

# 6 Alignment between plasticity and evolutionary divergence

## 6.1 Angle plasticity of A and evolutionary divergence of B

```
# test for uniform distribution
ks.test(logit(ma1$angle_PVa_EDb_mean,inv=TRUE),"punif")
```

```
##
##  One-sample Kolmogorov-Smirnov test
##
## data:  logit(ma1$angle_PVa_EDb_mean, inv = TRUE)
## D = 0.15077, p-value = 0.05172
## alternative hypothesis: two-sided
```

```
par(mfrow=c(1,2))
hist(ma1$angle_PVa_EDb_mean,col="#5D9CEC",xlab="logit scale",main="")
hist(logit(ma1$angle_PVa_EDb_mean,inv=TRUE)*180,col="#5D9CEC", xlab="linear scale",main="")
```

```
# Create VCV sampling variance matrix
V4 <- create.VCV.matrix(ma1$angle_PVa_EDb_sd,ma1$env.anc,ma1$env.nov,ma1$substudy.id)

# intercept model
fit4.0.1 <- rma.mv(angle_PVa_EDb_mean ~ 1, V = V4, data=ma1,
                   random = list(~1|study.id, ~1|err))
```

```
## Warning in rma.mv(angle_PVa_EDb_mean ~ 1, V = V4, data = ma1, random =
## list(~1 | : 'V' appears to be not positive definite.
```

```
fit4.0.2 <- rma.mv(angle_PVa_EDb_mean ~ 1, V = V4, data=ma1,
                   random = list(~1|spp,~1|err), R = list(spp = A_ma1))
```

```
## Warning in rma.mv(angle_PVa_EDb_mean ~ 1, V = V4, data = ma1, random =
## list(~1 | : 'V' appears to be not positive definite.
```

```
fit4.0.3 <- rma.mv(angle_PVa_EDb_mean ~ 1, V = V4, data=ma1,
                   random = list(~1|study.id,~1|spp,~1|err), R = list(spp = A_ma1))
```

```
## Warning in rma.mv(angle_PVa_EDb_mean ~ 1, V = V4, data = ma1, random =
```

```
## list(~1 | : 'V' appears to be not positive definite.
```

```r
round(fitstats(fit4.0.1,fit4.0.2,fit4.0.3),2)
```

```
##           fit4.0.1 fit4.0.2 fit4.0.3
## logLik:    -132.40  -132.40  -132.40
## deviance:   264.80   264.80   264.80
## AIC:        270.80   270.80   272.80
## BIC:        277.84   277.84   282.18
## AICc:       271.13   271.13   273.36
```

```r
print(fit4.0.1)
```

```
##
## Multivariate Meta-Analysis Model (k = 78; method: REML)
##
## Variance Components:
##
##              estim    sqrt  nlvls  fixed    factor
## sigma^2.1  0.0000  0.0000     34     no  study.id
## sigma^2.2  1.3845  1.1767     78     no       err
##
## Model Results:
##
## estimate      se    zval    pval   ci.lb   ci.ub
##   0.3586  0.1573  2.2794  0.0226  0.0503  0.6669  *
##
## ---
## Signif. codes:  0 '***' 0.001 '**' 0.01 '*' 0.05 '.' 0.1 ' ' 1
```

```r
print.to.angle(fit4.0.1, max.angle = 180)
```

```
## [1] "Angle: 105.97 - CI: 92.26 to 118.95"
## [1] "Intercept minus one unit:"
## numeric(0)
## [1] "Intercept plus one unit:"
## numeric(0)
```

```r
# add covariates
fit4.1.1 <- rma.mv(angle_PVa_EDb_mean ~ scale(n.traits) + fitness + timing.traits,
                   V = V4, data=ma1, random = list(~1|study.id, ~1|err))
```

```
## Warning in rma.mv(angle_PVa_EDb_mean ~ scale(n.traits) + fitness +
## timing.traits, : 'V' appears to be not positive definite.
```

```r
print(fit4.1.1)
```

```
##
## Multivariate Meta-Analysis Model (k = 78; method: REML)
##
## Variance Components:
##
##              estim    sqrt  nlvls  fixed    factor
## sigma^2.1  0.0000  0.0029     34     no  study.id
## sigma^2.2  1.4085  1.1868     78     no       err
##
## Test of Moderators (coefficient(s) 2:4):
## QM(df = 3) = 2.1093, p-val = 0.5500
```

```
##
## Model Results:
##
##                   estimate      se     zval    pval    ci.lb   ci.ub
## intrcpt             0.2152  0.2441   0.8816  0.3780  -0.2632  0.6935
## scale(n.traits)    -0.1265  0.1708  -0.7408  0.4588  -0.4612  0.2082
## fitness             0.1529  0.1798   0.8506  0.3950  -0.1994  0.5053
## timing.traits       0.2420  0.4872   0.4968  0.6193  -0.7128  1.1968
##
## ---
## Signif. codes:  0 '***' 0.001 '**' 0.01 '*' 0.05 '.' 0.1 ' ' 1
```

### 6.2 Correlation between angle between evolutionary divergence and plasticity and the contribution of plasticity to total divergence.

```
# Mean change
colMeans(PV_corrs)
```

```
##             all no-overshooters
##      0.06345093     -0.64479582
```

```
# Condfidence intervals
apply(PV_corrs,2,quantile,probs=c(0.025,0.975))
```

```
##              all no-overshooters
## 2.5%  -0.1855899      -0.7422293
## 97.5%  0.3302196      -0.5296443
```

```
# plot
par(mfrow=c(2,2))
hist(PV_corrs[,1],col="#5D9CEC",xlab="PV-ED - PV/TD",main="all")
abline(v=0,col="red")
hist(PV_corrs[,2],col="#5D9CEC",xlab="PV-ED - PV/TD",main="no overshooters")
abline(v=0,col="red")
cols <- ifelse(ma1$PVa_on_TDb_mean>1,"#5D9CEC",ifelse(ma1$PVa_on_TDb_mean<0,"#CD2626","purple"))
plot(logit(ma1$angle_PVa_EDb_mean,inv=TRUE)*180,ma1$PVa_on_TDb_mean,xlab="Angle PV-ED",
     ylab="Length PV/TD",pch=16,col=cols)
abline(h=0,lty=2)
plot(logit(ma1$angle_PVa_TDb_mean,inv=TRUE)*180,logit(ma1$angle_PVa_EDb_mean,inv=TRUE)*180,
     xlab="Angle PV-TD",ylab="Angle PV-ED",pch=16,col=cols)
abline(v=90,lty=2)
```

### 6.3 Angle evolutionary divergence of B and Pmax for AinB

```
# test for uniform distribution
ks.test(logit(ma2$angle_Pab_EDb_mean,inv=TRUE),"punif")
```

```
##
##  One-sample Kolmogorov-Smirnov test
##
## data:  logit(ma2$angle_Pab_EDb_mean, inv = TRUE)
## D = 0.19109, p-value = 0.03116
## alternative hypothesis: two-sided
```

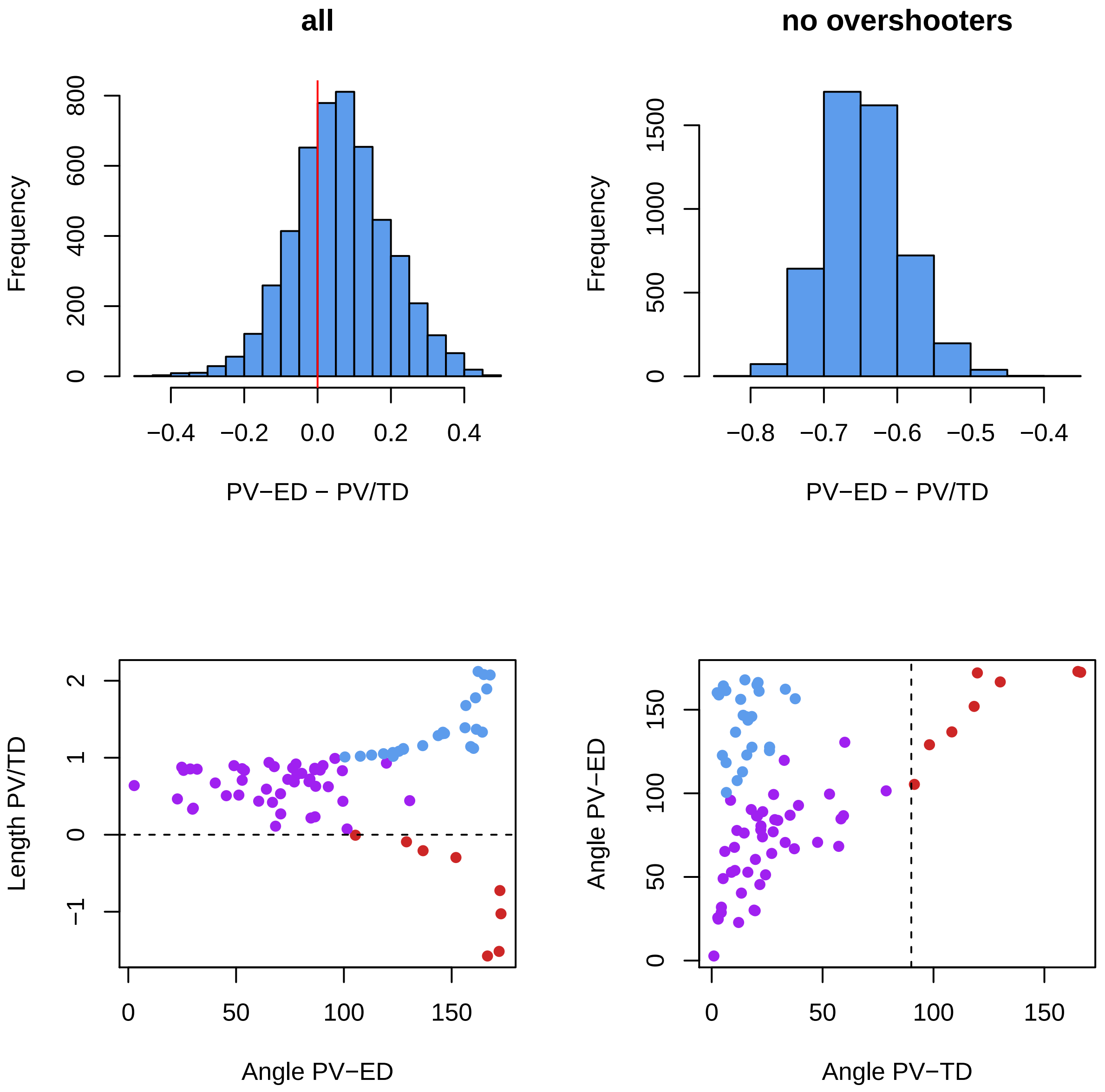


Figure 9: (a) Correlation between the angle between evolutionary divergence and plasticity and the contribution of plasticity to total divergence. (b) Mean angle between evolutionary divergence and plasticity versus the mean contribution of plasticity to total divergence for each study. (c) Mean angle between total divergence and plasticity versus the mean angle between evolutionary divergence and plasticity versus. Dots in red indicate poulations with maladaptive plastic responses, in which the plastic response does not contribute to the tota divergence, dots in blue indicate populations in which plastic responses overshoot the selective optimum and dots in purple are are populations in which the plastic response contributed to total divergence.

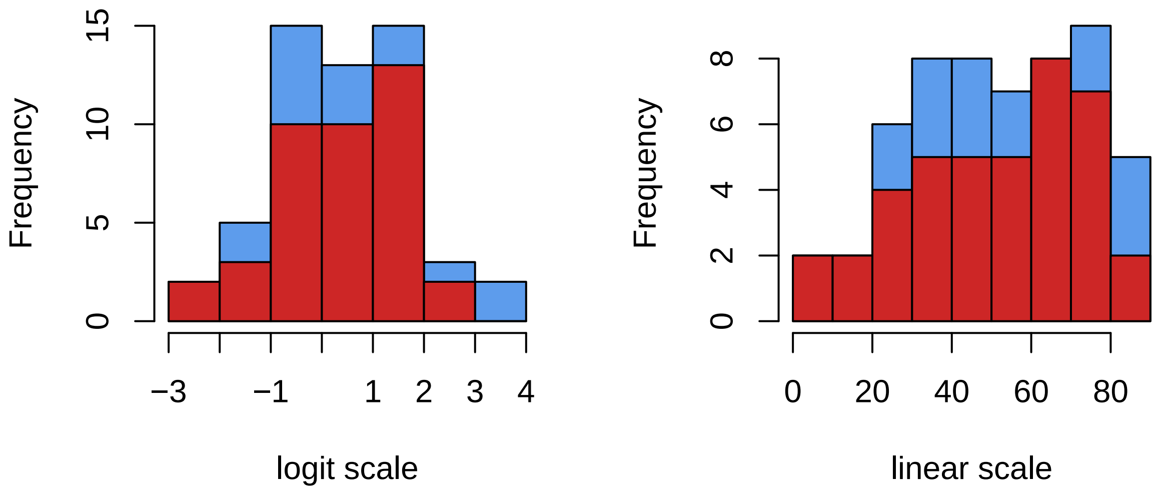


Figure 10: Angle evolutionary divergence of B and Pmax for BinA. In red morphological traits and in blue timing traits (stacked).

```
par(mfrow=c(1,2))
xx <- hist(ma2$angle_Pab_EDb_mean,col="#5D9CEC",xlab="logit scale",main="")
hist(ma2$angle_Pab_EDb_mean[ma2$timing.traits<0.5],
     col="#CD2626",breaks=xx$breaks,add=TRUE)
xx <- hist(logit(ma2$angle_Pab_EDb_mean,inv=TRUE)*90,col="#5D9CEC", xlab="linear scale",main="")
hist(logit(ma2$angle_Pab_EDb_mean[ma2$timing.traits<0.5],inv=TRUE)*90,
     col="#CD2626",breaks=xx$breaks,add=TRUE)
```

```
# Create VCV sampling variance matrix
V6 <- create.VCV.matrix(ma2$angle_Pab_EDb_sd,ma2$env.anc,ma2$env.nov,ma2$substudy.id)

# intercept only
fit6.0.1 <- rma.mv(angle_Pab_EDb_mean ~ 1, V =V6, data=ma2,
                   random = list(~1|study.id, ~1|err))
```

```
## Warning in rma.mv(angle_Pab_EDb_mean ~ 1, V = V6, data = ma2, random =
## list(~1 | : 'V' appears to be not positive definite.
```

```
fit6.0.2 <- rma.mv(angle_Pab_EDb_mean ~ 1, V =V6, data=ma2,
                   random = list(~1|spp, ~1|err), R = list(spp = A_ma2))
```

```
## Warning in rma.mv(angle_Pab_EDb_mean ~ 1, V = V6, data = ma2, random =
## list(~1 | : 'V' appears to be not positive definite.
```

```
fit6.0.3 <- rma.mv(angle_Pab_EDb_mean ~ 1, V =V6, data=ma2,
                   random = list(~1|study.id, ~1|spp, ~1|err), R = list(spp = A_ma2))
```

```
## Warning in rma.mv(angle_Pab_EDb_mean ~ 1, V = V6, data = ma2, random =
## list(~1 | : 'V' appears to be not positive definite.
```

```
round(fitstats(fit6.0.1,fit6.0.2,fit6.0.3),2)
```

```
##           fit6.0.1 fit6.0.2 fit6.0.3
## logLik:     -88.30   -88.30   -87.71
## deviance:   176.60   176.60   175.41
## AIC:        182.60   182.60   183.41
## BIC:        188.57   188.57   191.37
```

```
## AICc:       183.08   183.08   184.23
```

```r
print(fit6.0.1)
```

```
##
## Multivariate Meta-Analysis Model (k = 55; method: REML)
##
## Variance Components:
##
##              estim    sqrt  nlvls  fixed    factor
## sigma^2.1  0.0000  0.0000     22     no  study.id
## sigma^2.2  0.5893  0.7676     55     no       err
##
## Model Results:
##
## estimate      se    zval    pval    ci.lb   ci.ub
##   0.1292  0.2078  0.6220  0.5339  -0.2780  0.5364
##
## ---
## Signif. codes:  0 '***' 0.001 '**' 0.01 '*' 0.05 '.' 0.1 ' ' 1
```

```r
print.to.angle(fit6.0.1)
```

```
## [1] "Angle: 47.9 - CI: 38.79 to 56.79"
## [1] "Intercept minus one unit:"
## numeric(0)
## [1] "Intercept plus one unit:"
## numeric(0)
```

```r
# add covariates
fit6.1.1 <- rma.mv(angle_Pab_EDb_mean ~ scale(n.traits) + fitness + timing.traits,
                   V = V6, data=ma2, random = list(~1|study.id, ~1|err))
```

```
## Warning in rma.mv(angle_Pab_EDb_mean ~ scale(n.traits) + fitness +
## timing.traits, : 'V' appears to be not positive definite.
```

```r
print(fit6.1.1)
```

```
##
## Multivariate Meta-Analysis Model (k = 55; method: REML)
##
## Variance Components:
##
##              estim    sqrt  nlvls  fixed    factor
## sigma^2.1  0.0000  0.0001     22     no  study.id
## sigma^2.2  0.4811  0.6936     55     no       err
##
## Test of Moderators (coefficient(s) 2:4):
## QM(df = 3) = 11.1539, p-val = 0.0109
##
## Model Results:
##
##                  estimate      se     zval    pval    ci.lb   ci.ub
## intrcpt           -0.3977  0.3198  -1.2438  0.2136  -1.0245  0.2290
## scale(n.traits)    0.6219  0.2282   2.7247  0.0064   0.1746  1.0693  **
## fitness            0.1742  0.2059   0.8458  0.3976  -0.2294  0.5778
## timing.traits      1.8678  0.6469   2.8873  0.0039   0.5999  3.1356  **
```

```
##
## ---
## Signif. codes:  0 '***' 0.001 '**' 0.01 '*' 0.05 '.' 0.1 ' ' 1
```

```
print.to.angle(fit6.1.1)
```

```
## [1] "Angle: 36.17 - CI: 23.77 to 50.13"
## [1] "Intercept minus one unit:"
## scale(n.traits)         fitness   timing.traits
##           23.86           32.47            8.46
## [1] "Intercept plus one unit:"
## scale(n.traits)         fitness   timing.traits
##           50.02           39.99           73.18
```

# 7 Produce polar plots

```
load(file="ma-retr-save-simulations-for-graphs.Rdata")

start.angle <- 180
clock.wise <- TRUE
legend.loc <- "bottomright"
cols <- ifelse(colMeans(PVa_on_TDb)<=0,"red",
               ifelse(colMeans(PVa_on_TDb)>1,"blue","purple"))
pdf(file="ma-retr-angle-plots.pdf",height = 12,width = 10, useDingbats = FALSE)
par(mfrow=c(2,2))
# Plasticity vs Evolutionary Divergence
rad.max <- 0.5
polar.plot(sqrt(density(angle_PVa_EDb[,1],from=0,to=180)$y),
           density(angle_PVa_EDb[,1],from=0,to=180)$x,start=start.angle,
           clockwise = clock.wise,line.col="#00000040",poly.col="#00000040",
           rp.type="p",radial.lim=c(0,rad.max),radial.labels=NA,
           main="Plasticity vs Evolutionary Divergence",
           label.pos=c(0,30,60,90,120,150,180),labels=c(0,30,60,90,120,150,180))
for(i in 2:dim(angle_PVa_EDb)[2]){
  polar.plot(sqrt(density(angle_PVa_EDb[,i],from=0,to=180)$y),
             density(angle_PVa_EDb[,i],from=0,to=180)$x,start=start.angle,
             clockwise = clock.wise,line.col="#00000040",poly.col="#00000040",
             rp.type="p",radial.lim=c(0,rad.max),add=TRUE)
}
polar.plot(rep(rad.max,dim(angle_PVa_EDb)[2]),colMeans(angle_PVa_EDb),
           start=start.angle,clockwise = clock.wise,rp.type="s",
           radial.lim=c(0,rad.max),point.col=cols,point.symbols=16,add=TRUE)
polar.plot(rad.max,logit(fit4.0.1$beta[1,1],inv=TRUE)*180,start=start.angle,
           clockwise = clock.wise,rp.type="r",radial.lim=c(0,rad.max),lwd=3,add=TRUE)
polar.plot(rep(rad.max,2),c(logit(fit4.0.1$ci.lb[1],inv=TRUE)*180,
                            logit(fit4.0.1$ci.ub[1],inv=TRUE)*180),start=start.angle,
           clockwise = clock.wise,rp.type="r",radial.lim=c(0,rad.max),add=TRUE)
legend(legend.loc,c("overshoot","undershoot","opposing direction"),
       col=c("blue","purple","red"),pch=16,
       title="Projection of plasticity on total divergence",cex=0.6,bg="white")

rad.max <- 0.7
#Plasticity vs Total Divergence
polar.plot(sqrt(density(angle_PVa_TDb[,1],from=0,to=180)$y),
```

```
           density(angle_PVa_TDb[,1],from=0,to=180)$x,start=start.angle,
           clockwise = clock.wise,line.col="#00000040",poly.col="#00000040",
           rp.type="p",radial.lim=c(0,rad.max),radial.labels=NA,
           main="Plasticity vs Total Divergence",
           label.pos=c(0,30,60,90,120,150,180),labels=c(0,30,60,90,120,150,180))
for(i in 2:dim(angle_PVa_TDb)[2]){
  polar.plot(sqrt(density(angle_PVa_TDb[,i],from=0,to=180)$y),
             density(angle_PVa_TDb[,i],from=0,to=180)$x,start=start.angle,
             clockwise = clock.wise,line.col="#00000040",poly.col="#00000040",
             rp.type="p",radial.lim=c(0,rad.max),add=TRUE)
}
polar.plot(rep(rad.max,dim(angle_PVa_TDb)[2]),colMeans(angle_PVa_TDb),
           start=start.angle,clockwise = clock.wise,rp.type="s",
           radial.lim=c(0,rad.max),point.col=cols,point.symbols=16,add=TRUE)
polar.plot(rad.max,logit(fit3.0.1$beta[1,1],inv=TRUE)*180,start=start.angle,
           clockwise = clock.wise,rp.type="r",radial.lim=c(0,rad.max),lwd=3,add=TRUE)
polar.plot(rep(rad.max,2),c(logit(fit3.0.1$ci.lb[1],inv=TRUE)*180,
                            logit(fit3.0.1$ci.ub[1],inv=TRUE)*180),start=start.angle,
           clockwise = clock.wise,rp.type="r",radial.lim=c(0,rad.max),add=TRUE)
legend(legend.loc,c("overshoot","undershoot","opposing direction"),col=c("blue","purple","red"),pch=16,

rad.max <- 0.6
#Plasticity A vs Plasticity B
polar.plot(sqrt(density(angle_PVa_PVb[,1],from=0,to=180)$y),
           density(angle_PVa_PVb[,1],from=0,to=180)$x,start=start.angle,
           clockwise = clock.wise,line.col="#00000040",poly.col="#00000040",
           rp.type="p",radial.lim=c(0,rad.max),radial.labels=NA,
           main="Plasticity A vs Plasticity B",
           label.pos=c(0,30,60,90,120,150,180),labels=c(0,30,60,90,120,150,180))
for(i in 2:dim(angle_PVa_PVb)[2]){
  polar.plot(sqrt(density(angle_PVa_PVb[,i],from=0,to=180)$y),
             density(angle_PVa_PVb[,i],from=0,to=180)$x,start=start.angle,
             clockwise = clock.wise,line.col="#00000040",poly.col="#00000040",
             rp.type="p",radial.lim=c(0,rad.max),add=TRUE)
}
polar.plot(rad.max,logit(fit9.0.1$beta[1,1],inv=TRUE)*180,start=start.angle,
           clockwise = clock.wise,rp.type="r",radial.lim=c(0,rad.max),lwd=3,add=TRUE)
polar.plot(rep(rad.max,2),c(logit(fit9.0.1$ci.lb[1],inv=TRUE)*180,
                            logit(fit9.0.1$ci.ub[1],inv=TRUE)*180),start=start.angle,
           clockwise = clock.wise,rp.type="r",radial.lim=c(0,rad.max),add=TRUE)
polar.plot(rep(rad.max,dim(angle_PVa_PVb)[2]),colMeans(angle_PVa_PVb),start=start.angle,
           clockwise = clock.wise,rp.type="s",radial.lim=c(0,rad.max),point.col=cols,
           point.symbols=16,add=TRUE)
legend(legend.loc,c("overshoot","undershoot","opposing direction"),col=c("blue","purple","red"),pch=16,

cols <- ifelse(ma2$morph.traits==0,"red","blue")
rad.max <- 0.5
#P_max_AB vs Evolutionary Divergence
polar.plot(sqrt(density(angle_Pab_EDb[,1],from=0,to=90)$y),
           density(angle_Pab_EDb[,1],from=0,to=90)$x,start=start.angle,
           clockwise = clock.wise,line.col="#00000040",poly.col="#00000040",
           rp.type="p",radial.lim=c(0,rad.max),radial.labels=NA,
           main="P_max_AB vs Evolutionary Divergence",
```

```r
           label.pos=c(0,30,60,90),labels=c(0,30,60,90))
for(i in 2:dim(angle_Pab_EDb)[2]){
  polar.plot(sqrt(density(angle_Pab_EDb[,i],from=0,to=90)$y),
             density(angle_Pab_EDb[,i],from=0,to=90)$x,start=start.angle,
             clockwise = clock.wise,line.col="#00000040",poly.col="#00000040",
             rp.type="p",radial.lim=c(0,rad.max),add=TRUE)
}
polar.plot(rad.max,logit(fit6.0.1$beta[1,1],inv=TRUE)*90,start=start.angle,
           clockwise = clock.wise,rp.type="r",radial.lim=c(0,rad.max),lwd=3,add=TRUE)
polar.plot(rep(rad.max,2),c(logit(fit6.0.1$ci.lb[1],inv=TRUE)*90,
                            logit(fit6.0.1$ci.ub[1],inv=TRUE)*90),start=start.angle,
           clockwise = clock.wise,rp.type="r",radial.lim=c(0,rad.max),add=TRUE)
polar.plot(rep(rad.max,dim(angle_Pab_EDb)[2]),colMeans(angle_Pab_EDb),start=start.angle,
           clockwise = clock.wise,rp.type="s",radial.lim=c(0,rad.max),point.col=cols,
           point.symbols=16,add=TRUE)
legend(legend.loc,c(">50% morphology","timing only"),col=c("blue","red"),pch=16,title="Type of traits",
dev.off()
```

```
## pdf
##   2
```

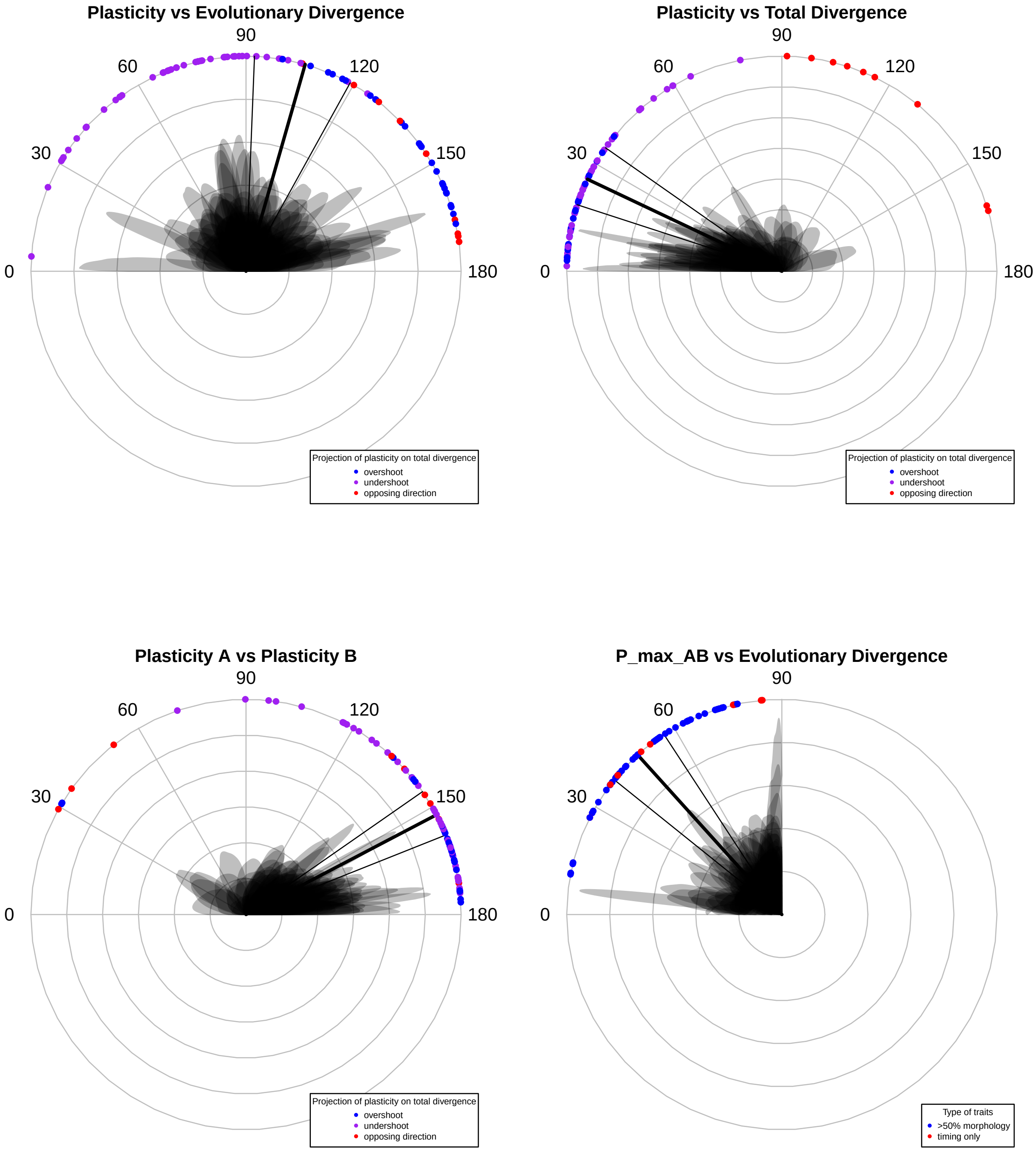


# 8 Ambiguous ancestry

For some of the studies the ancestry of the populations is ambiguous. Here we test whether the findings are affected by this. We select study with an ambiguous ancestry and randomize the ancestry. The following table shows how we categorized the studies, based on the information where the ancestry was based on.

| Non-ambiguous ancestries | Ambiguous ancestries |
| --- | --- |
| highest fitness in own environment | most south on northern hemisphere |
| most benign environment | most inland populations (as opposed to coastal) |
| more central in geographic range | lowest altitude |
| most natural environment | other reasons |
| indicated by the authors, mostly referring to other studies | |
| historical records of invasion | |

In each randomizations we randomly allocate ancestry for all comprisons with ambiguous ancestries. We rerun the most important analyses and stored the model output for plotting.

```
# number of randomizations
no.rand <- 1000

# data frame for storage of outcomes
output.rand <- data.frame(angle_PVa_EDb_est=rep(NA,no.rand),angle_PVa_EDb_lw=NA,
                          angle_PVa_EDb_up=NA,angle_PVa_TDb_est=NA,angle_PVa_TDb_lw=NA,
                          angle_PVa_TDb_up=NA,angle_Pab_EDb_est=NA,angle_Pab_EDb_lw=NA,
                          angle_Pab_EDb_up=NA,angle_Pab_EDb_trait_est=NA,
                          angle_Pab_EDb_trait_lw=NA,angle_Pab_EDb_trait_up=NA)

# run randomizations
for(t in 1:no.rand){
  ## dataset ma1
  #################################################
  # which rows to randomize
  rows.to.rand <- which(ma1$anc.cat %in% c("SN","IC","AL","OT"))
  # which rows to swap ancestry
  rows.to.swap <- rows.to.rand[which(rbinom(length(rows.to.rand),1,0.5)==1)]

  # swap effect sizes in ma1
  ma1.rand <- ma1
  ma1.rand$angle_PVa_EDb_mean[rows.to.swap] <- ma1.rand$angle_PVb_EDa_mean[rows.to.swap]
  ma1.rand$angle_PVa_EDb_sd[rows.to.swap] <- ma1.rand$angle_PVb_EDa_sd[rows.to.swap]
  ma1.rand$angle_PVa_TDb_mean[rows.to.swap] <- ma1.rand$angle_PVb_TDa_mean[rows.to.swap]
  ma1.rand$angle_PVa_TDb_sd[rows.to.swap] <- ma1.rand$angle_PVb_TDa_sd[rows.to.swap]

  ## dataset ma2
  #################################################
  # which rows to randomize
  rows.to.rand <- which(ma2$anc.cat %in% c("SN","IC","AL","OT"))
  # which rows to swap ancestry
  rows.to.swap <- rows.to.rand[which(rbinom(length(rows.to.rand),1,0.5)==1)]

  # swap effect sizes in ma1
  ma2.rand <- ma2
  ma2.rand$angle_Pab_EDb_mean[rows.to.swap] <- ma2.rand$angle_Pba_EDa_mean[rows.to.swap]
  ma2.rand$angle_Pab_EDb_sd[rows.to.swap] <- ma2.rand$angle_Pba_EDa_sd[rows.to.swap]

  ## rerun analysis for angle_PVa_EDb
  #################################################
  # Create VCV sampling variance matrix
  V4.rand <- create.VCV.matrix(ma1.rand$angle_PVa_EDb_sd,ma1.rand$env.anc,
```

```
                             ma1.rand$env.nov,ma1.rand$substudy.id)
  # run model
  fit4.rand <- rma.mv(angle_PVa_EDb_mean ~ 1, V = V4.rand,
                      data=ma1.rand, random = list(~1|study.id, ~1|err))

  # store output
  output.rand$angle_PVa_EDb_est[t] <- fit4.rand$beta[1,1]
  output.rand$angle_PVa_EDb_lw[t] <- fit4.rand$ci.lb[1]
  output.rand$angle_PVa_EDb_up[t] <- fit4.rand$ci.ub[1]

  ## rerun analysis for angle_PVa_TDb
  #################################################
  # Create VCV sampling variance matrix
  V3.rand <- create.VCV.matrix(ma1.rand$angle_PVa_TDb_sd,ma1.rand$env.anc,
                             ma1.rand$env.nov,ma1.rand$substudy.id)

  # run model
  fit3.rand <- rma.mv(angle_PVa_TDb_mean ~ 1,
                   V = V3.rand, data=ma1.rand, random = list(~1|study.id, ~1|err))

  # store output
  output.rand$angle_PVa_TDb_est[t] <- fit3.rand$beta[1,1]
  output.rand$angle_PVa_TDb_lw[t] <- fit3.rand$ci.lb[1]
  output.rand$angle_PVa_TDb_up[t] <- fit3.rand$ci.ub[1]

  ## rerun analysis for angle_Pab_EDb
  #################################################
  # Create VCV sampling variance matrix
  V6.rand <- create.VCV.matrix(ma2.rand$angle_Pab_EDb_sd,ma2.rand$env.anc,
                             ma2.rand$env.nov,ma2.rand$substudy.id)

  # run model
  fit6.rand <- rma.mv(angle_Pab_EDb_mean ~ 1,
                   V = V6.rand, data=ma2.rand, random = list(~1|study.id, ~1|err))

  # store output
  output.rand$angle_Pab_EDb_est[t] <- fit6.rand$beta[1,1]
  output.rand$angle_Pab_EDb_lw[t] <- fit6.rand$ci.lb[1]
  output.rand$angle_Pab_EDb_up[t] <- fit6.rand$ci.ub[1]

  ## rerun analysis for angle_Pab_EDb with covariates
  #################################################
  # run model
  fit6.rand2 <-rma.mv(angle_Pab_EDb_mean~ scale(n.traits)+fitness+timing.traits,
                      V = V6.rand, data=ma2.rand, random =list(~1|study.id,~1|err))

  # store output
  output.rand$angle_Pab_EDb_trait_est[t] <- fit6.rand2$beta[4,1]
  output.rand$angle_Pab_EDb_trait_lw[t] <- fit6.rand2$ci.lb[4]
  output.rand$angle_Pab_EDb_trait_up[t] <- fit6.rand2$ci.ub[4]
}
```

Now plot the output of the randomizations.

```r
# set plooting parameters
par(mfrow=c(1,4),mar=c(4,2.5,2,0.5),cex.main=0.85)

## plot (a) angle_PVa_EDb
#################################################
# order randomizations by mean
plot.order <- order(output.rand$angle_PVa_EDb_est)
# find the range of the estimates
plot.range <- c(logit(min(output.rand$angle_PVa_EDb_lw),inv=TRUE)*180,
         logit(max(output.rand$angle_PVa_EDb_up),inv=TRUE)*180)
# plot means
plot(logit(output.rand$angle_PVa_EDb_est[plot.order],inv=TRUE)*180,1:1000,pch=16,
     xlim=plot.range, col="#5D9CEC44",yaxt="n",ylab="ordered randomizations",
     xlab="intercept", main="(a) Plasticity-Evol. Div.")
# add y-axis
axis(2,at=c(0,0.2,0.4,0.6,0.8,1)*no.rand,labels=c(0,0.2,0.4,0.6,0.8,1),las=1)
# plot confidence intervals
segments(logit(output.rand$angle_PVa_EDb_lw[plot.order],inv=TRUE)*180,1:1000,
         logit(output.rand$angle_PVa_EDb_up[plot.order],inv=TRUE)*180,1:1000,
         col="#5D9CEC44")
# add mean and confidence intervals for the unrandomized analysis
abline(v=logit(fit4.0.1$beta[1,1],inv=TRUE)*180,col="red",lwd=2)
abline(v=logit(fit4.0.1$ci.lb[1],inv=TRUE)*180,col="red",lwd=1)
abline(v=logit(fit4.0.1$ci.ub[1],inv=TRUE)*180,col="red",lwd=1)
# add null expectation
abline(v=90,lty=2)

## plot (b) angle_PVa_TDb
#################################################
# order randomizations by mean
plot.order <- order(output.rand$angle_PVa_TDb_est)
# find the range of the estimates
plot.range <- c(logit(min(output.rand$angle_PVa_TDb_lw),inv=TRUE)*180,
         logit(max(output.rand$angle_PVa_TDb_up),inv=TRUE)*180)
# plot means
plot(logit(output.rand$angle_PVa_TDb_est[plot.order],inv=TRUE)*180,1:1000,pch=16,
     xlim=plot.range,col="#5D9CEC44",yaxt="n",ylab="ordered randomizations",
     xlab="intercept",main="(b) Plasticity-Tot. Div.")
# add y-axis
axis(2,at=c(0,0.2,0.4,0.6,0.8,1)*no.rand,labels=c(0,0.2,0.4,0.6,0.8,1),las=1)
# plot confidence intervals
segments(logit(output.rand$angle_PVa_TDb_lw[plot.order],inv=TRUE)*180,1:1000,
         logit(output.rand$angle_PVa_TDb_up[plot.order],inv=TRUE)*180,1:1000,
         col="#5D9CEC44")
# add mean and confidence intervals for the unrandomized analysis
abline(v=logit(fit3.0.1$beta[1,1],inv=TRUE)*180,col="red",lwd=2)
abline(v=logit(fit3.0.1$ci.lb[1],inv=TRUE)*180,col="red",lwd=1)
abline(v=logit(fit3.0.1$ci.ub[1],inv=TRUE)*180,col="red",lwd=1)

## plot (c) angle_Pab_EDb (intercept)
#################################################
# order randomizations by mean
plot.order <- order(output.rand$angle_Pab_EDb_est)
```

```
# find the range of the estimates
plot.range <- c(logit(min(output.rand$angle_Pab_EDb_lw),inv=TRUE)*90,
         logit(max(output.rand$angle_Pab_EDb_up),inv=TRUE)*90)
# plot means
plot(logit(output.rand$angle_Pab_EDb_est[plot.order],inv=TRUE)*90,1:1000,pch=16,
     xlim=plot.range,col="#5D9CEC44",yaxt="n",ylab="ordered randomizations",
     xlab="intercept",main="(c) P_max_AB - Evol. Div.")
# add y-axis
axis(2,at=c(0,0.2,0.4,0.6,0.8,1)*no.rand,labels=c(0,0.2,0.4,0.6,0.8,1),las=1)
axis(2,at=c(0,0.2,0.4,0.6,0.8,1)*no.rand,labels=c(0,0.2,0.4,0.6,0.8,1),las=1)
# plot confidence intervals
segments(logit(output.rand$angle_Pab_EDb_lw[plot.order],inv=TRUE)*90,1:1000,
         logit(output.rand$angle_Pab_EDb_up[plot.order],inv=TRUE)*90,1:1000,
         col="#5D9CEC44")
# add mean and confidence intervals for the unrandomized analysis
abline(v=logit(fit6.0.1$beta[1,1],inv=TRUE)*90,col="red",lwd=2)
abline(v=logit(fit6.0.1$ci.lb[1],inv=TRUE)*90,col="red",lwd=1)
abline(v=logit(fit6.0.1$ci.ub[1],inv=TRUE)*90,col="red",lwd=1)
# add null expectation
abline(v=45,lty=2)

## plot (d) angle_Pab_EDb (trait type)
#################################################
# order randomizations by mean
plot.order <- order(output.rand$angle_Pab_EDb_trait_est)
# find the range of the estimates
plot.range <- c(min(output.rand$angle_Pab_EDb_trait_lw),max(output.rand$angle_Pab_EDb_trait_up))
# plot means
plot(output.rand$angle_Pab_EDb_trait_est[plot.order],1:1000,pch=16,
     xlim=plot.range, col="#5D9CEC44",yaxt="n",ylab="ordered randomizations",
          xlab="trait type",main="(d) P_max_AB - Evol. Div.")
# add y-axis
axis(2,at=c(0,0.2,0.4,0.6,0.8,1)*no.rand,labels=c(0,0.2,0.4,0.6,0.8,1),las=1)
# plot confidence intervals
segments(output.rand$angle_Pab_EDb_trait_lw[plot.order],1:1000,
         output.rand$angle_Pab_EDb_trait_up[plot.order],1:1000,
         col="#5D9CEC44")
# add mean and confidence intervals for the unrandomized analysis
abline(v=fit6.1.1$beta[4,1],col="red",lwd=2)
abline(v=fit6.1.1$ci.lb[4],col="red",lwd=1)
abline(v=fit6.1.1$ci.ub[4],col="red",lwd=1)
# add null expectation
abline(v=0,lty=2)
```

# 9 Check for the seriousness of non-positive definite variance-covariance matrices

The variance-covariance matrices in the meta-analyses are not positive definite. By plotting the eigenvalues we can check whether the negative eigenvalues are very close to zero (so the VCV is close to positive definite) or not.

```
eigV1 <- eigen(V1)$values
eigV2 <- eigen(V2)$values
```

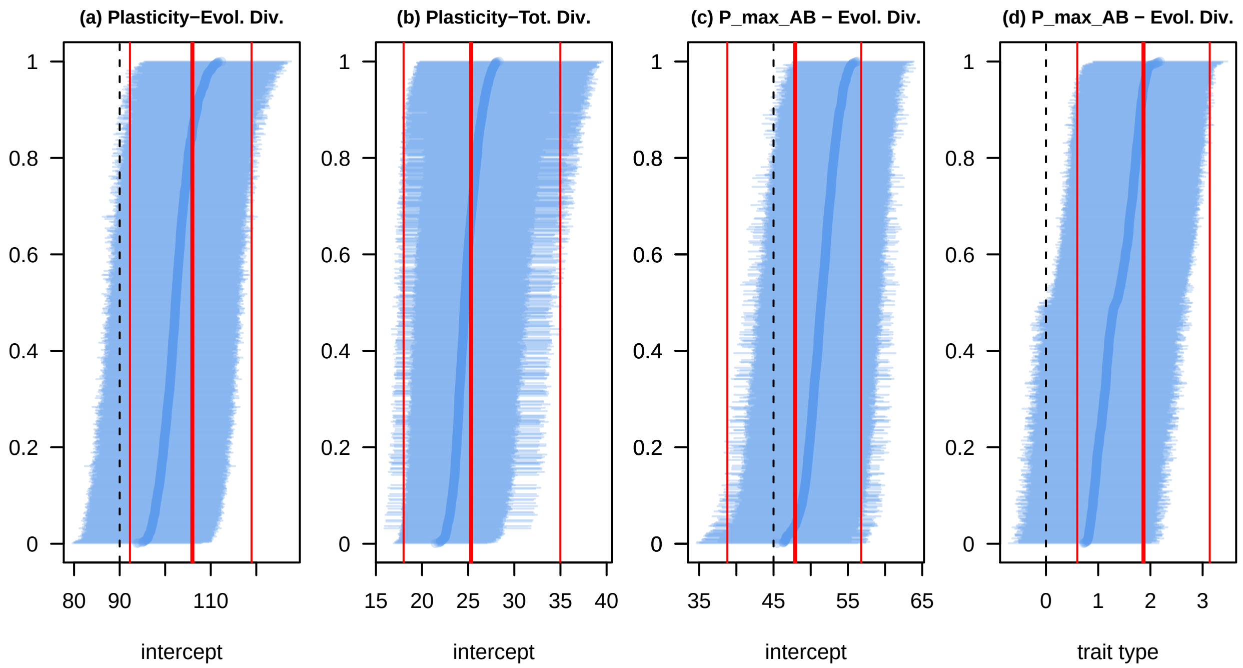


Figure 11: Estimated main effects when ambiguous ancestries are randomized for the angles between (a) plasticity and evolutionary divergence (intercept), (b) plasticity and total divergence (intercept) and (c,d) P_max_AB and evolutionary divergence (intercept and trait type). In blue are the means (dots) and credible intervals (bars) for 1000 randomizations, ordered by the mean. In red the means (bold) and credible intervals (thin) of the presented (unrandomized) analalyses. The vertical black dashed line indicates the mean null prediction.

```r
eigV3 <- eigen(V3)$values
eigV4 <- eigen(V4)$values
eigV5 <- eigen(V5)$values
eigV6 <- eigen(V6)$values
eigV8 <- eigen(V8)$values
eigV9 <- eigen(V9)$values

par(mfrow=c(3,3),mar=c(1,2,4,1))
barplot(eigV1,col=ifelse(eigV1>0,"#5D9CEC","red"),main="matrix V1")
barplot(eigV2,col=ifelse(eigV1>0,"#5D9CEC","red"),main="matrix V2")
barplot(eigV3,col=ifelse(eigV1>0,"#5D9CEC","red"),main="matrix V3")
barplot(eigV4,col=ifelse(eigV1>0,"#5D9CEC","red"),main="matrix V4")
barplot(eigV5,col=ifelse(eigV1>0,"#5D9CEC","red"),main="matrix V5")
barplot(eigV6,col=ifelse(eigV1>0,"#5D9CEC","red"),main="matrix V6")
barplot(eigV8,col=ifelse(eigV1>0,"#5D9CEC","red"),main="matrix V8")
barplot(eigV9,col=ifelse(eigV1>0,"#5D9CEC","red"),main="matrix V9")
```

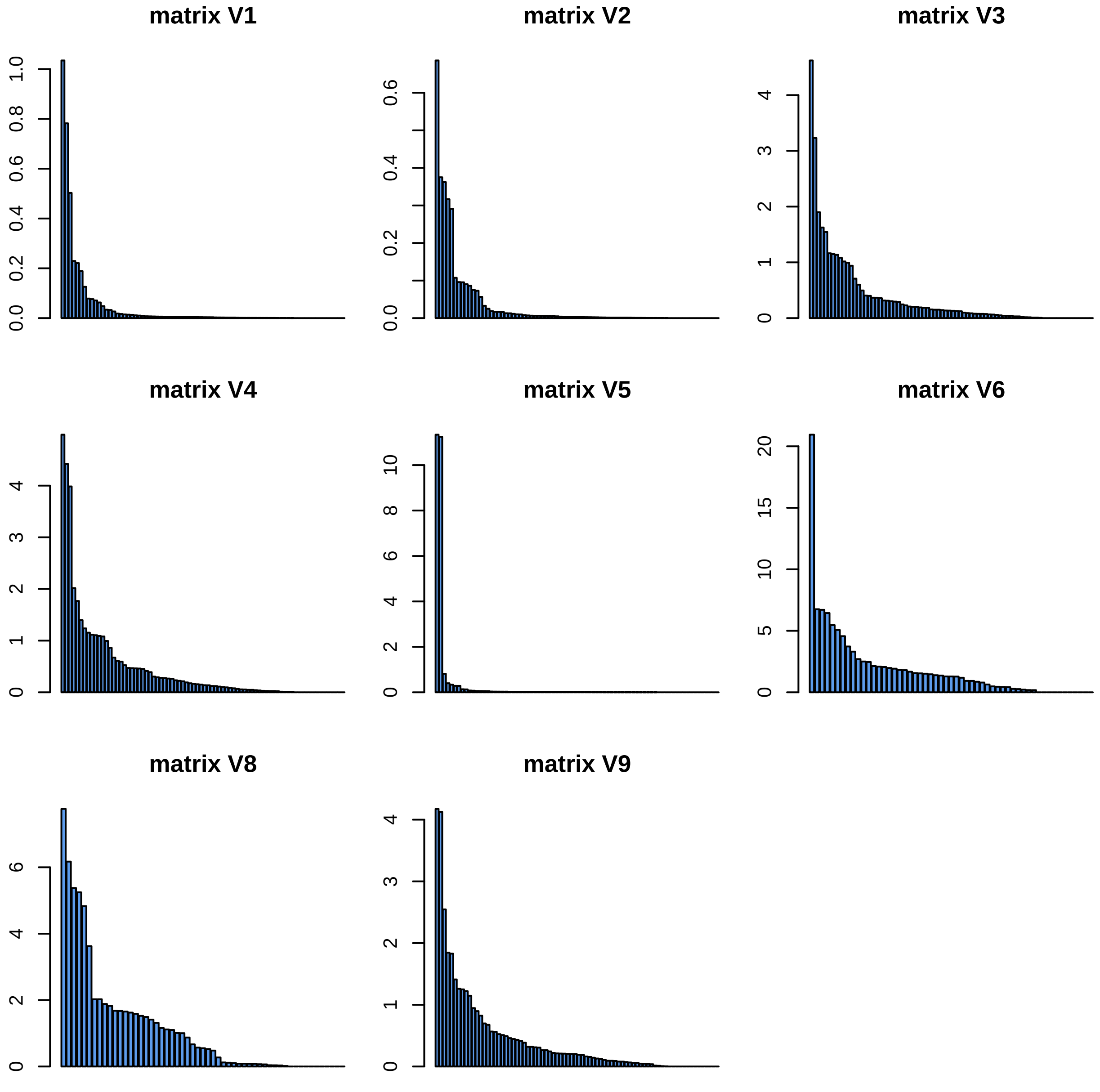


Figure 12: Eigenvalues of all VCV matrices
