# Supplementary material for: Plasticity leaves a phenotypic signature during local adaptation
Source: Evol Lett. 2020 Jun 9;4(4):360–70. doi: 10.1002/evl3.185 (PMC7403707; doi:10.1002/evl3.185)

Appendix S3: Plasticity takes the lead in local adaptation

Reinder Radersma, Daniel A. W. Noble & Tobias Uller

2020-03-06

1 Introduction

This document is an electronic supplement to:

> Reinder Radersma, Daniel A. W. Noble & Tobias Uller (2020) Plasticity takes the lead in local adaptation. Submitted to *Evolution Letters*.

It contains plots of the bivariate phenotypic trait means for all comparisons with 2 traits. These plots are real data representations of the vector analyses discribed in the main text and depicted in figure 1. The title of each plot is the citation of the study and which two environments are compared. The arrow from red to purple is the plasticity vector (AinA to AinB), while the arrow from purple to blue is the evolutionary divergence (AinB to BinB). The dashed line is the total phenotypic difference between the populations (AinA to BinB).

2 Chapin_Chapin_1981 - comparing Muskeg with Arctic

The study species is *Carex aquatilis*. The angle between pasticity vector and evolutionary divergence is 24.79°. The angle between pasticity vector and total phenotypic difference is 2.95°.

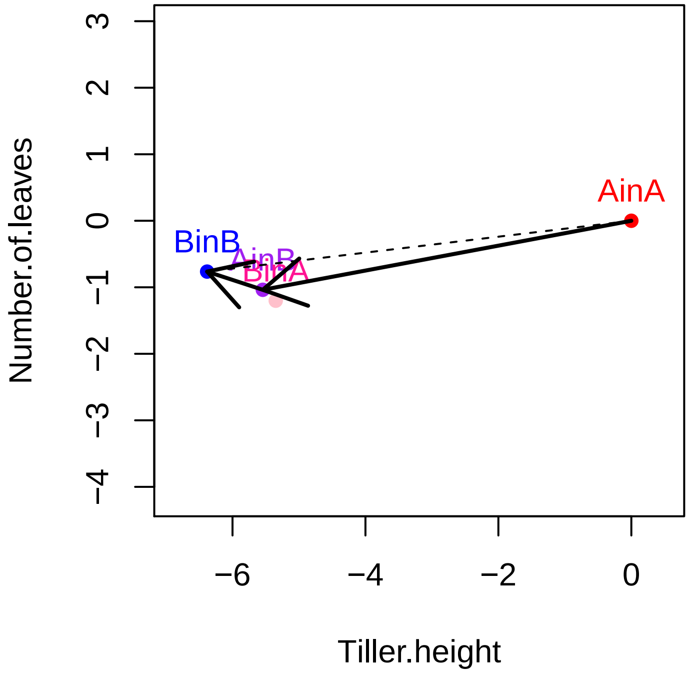

3 Chapin_Chapin_1981 - comparing Hot spring with Arctic

The study species is *Carex aquatilis*. The angle between pasticity vector and evolutionary divergence is 28.74°. The angle between pasticity vector and total phenotypic difference is 4.31°.

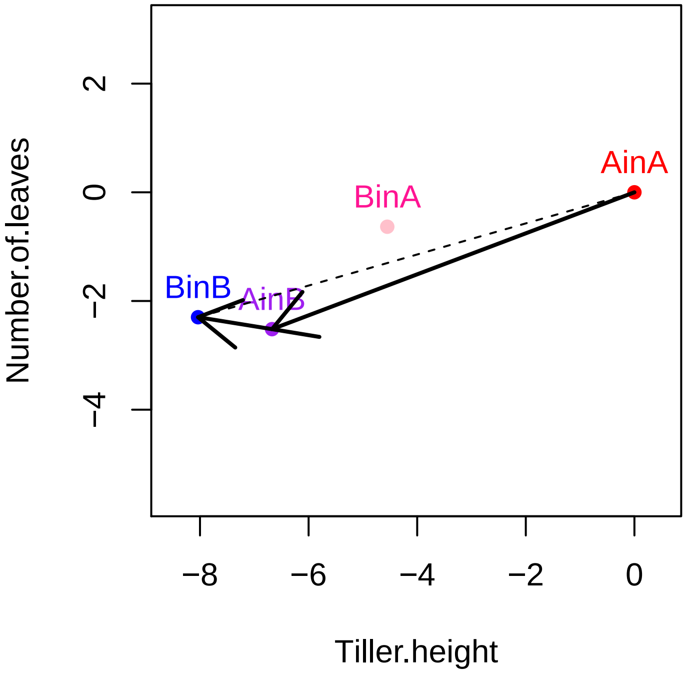

4 Chapin_Chapin_1981 - comparing Muskeg with Hot spring

The study species is *Carex aquatilis*. The angle between pasticity vector and evolutionary divergence is 161.44°. The angle between pasticity vector and total phenotypic difference is 6.39°.

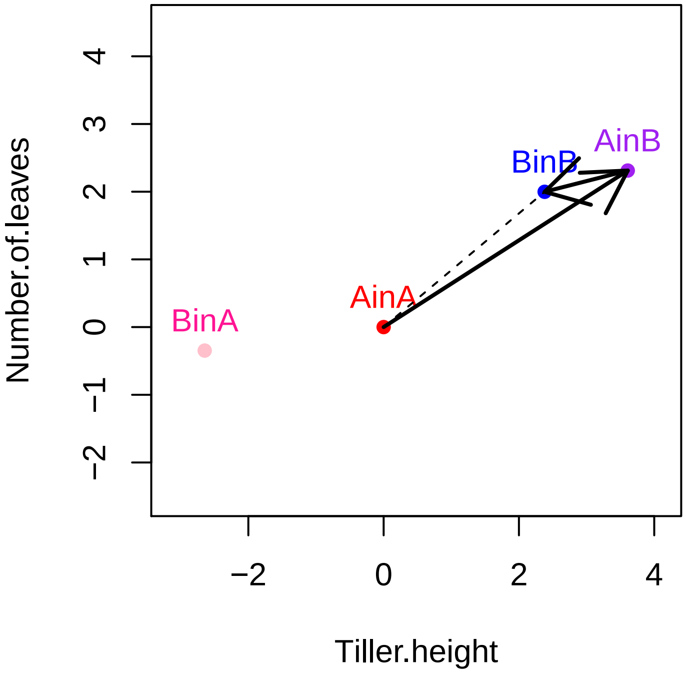

5 Chapin_Chapin_1981 - comparing Alpine with Muskeg

The study species is *Carex aquatilis*. The angle between pasticity vector and evolutionary divergence is 45.46°. The angle between pasticity vector and total phenotypic difference is 21.71°.

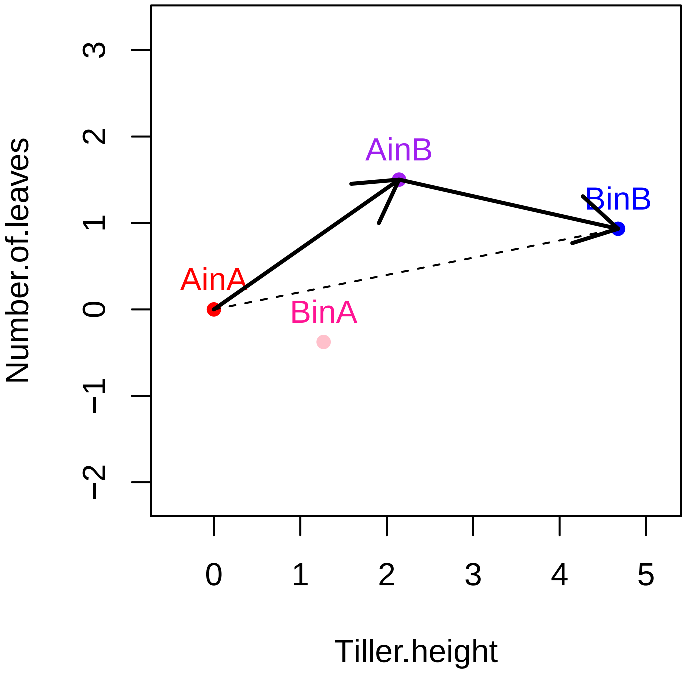

6 Chapin_Chapin_1981 - comparing Subalpine with Muskeg

The study species is *Carex aquatilis*. The angle between pasticity vector and evolutionary divergence is 151.97°. The angle between pasticity vector and total phenotypic difference is 118.33°.

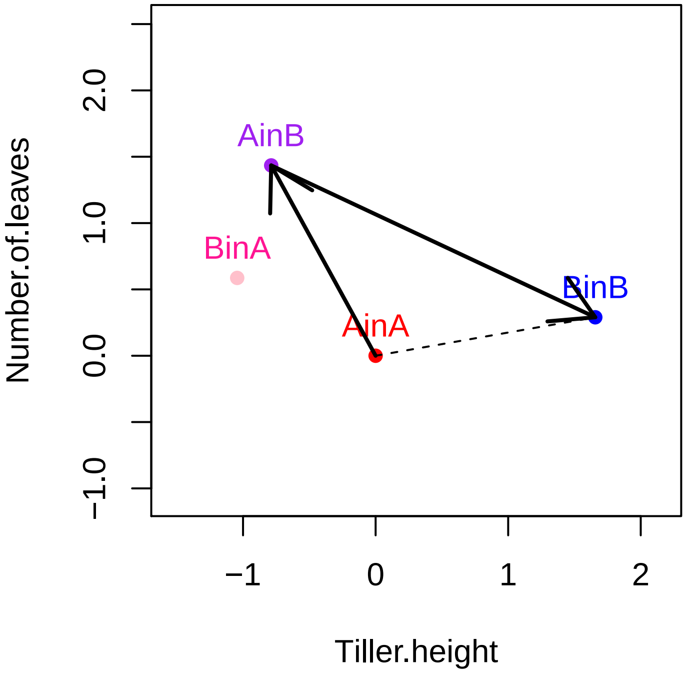

7 Chapin_Chapin_1981 - comparing Alpine with Hot spring

The study species is *Carex aquatilis*. The angle between pasticity vector and evolutionary divergence is 31.92°. The angle between pasticity vector and total phenotypic difference is 4.37°.

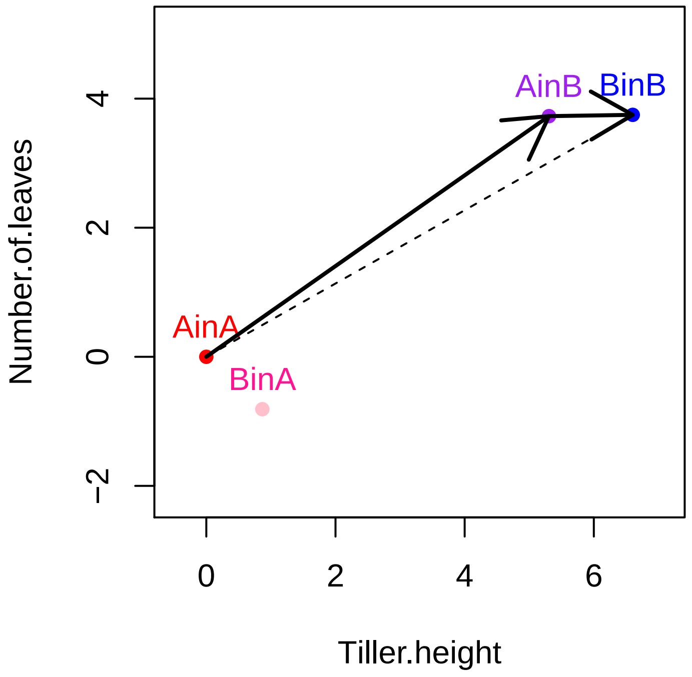

8 Chapin_Chapin_1981 - comparing Subalpine with Hot spring

The study species is *Carex aquatilis*. The angle between pasticity vector and evolutionary divergence is 122.68°. The angle between pasticity vector and total phenotypic difference is 4.81°.

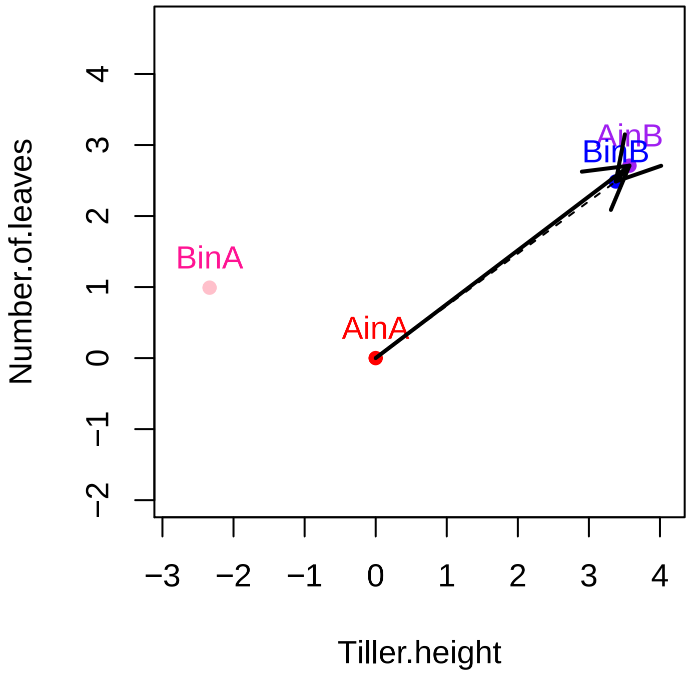

9 Etterson_2004 - comparing Kansas (KS) with Minnesota (MN)

The study species is *Chamaecrista fasciculata*. The angle between pasticity vector and evolutionary divergence is 164.92°. The angle between pasticity vector and total phenotypic difference is 20.41°.

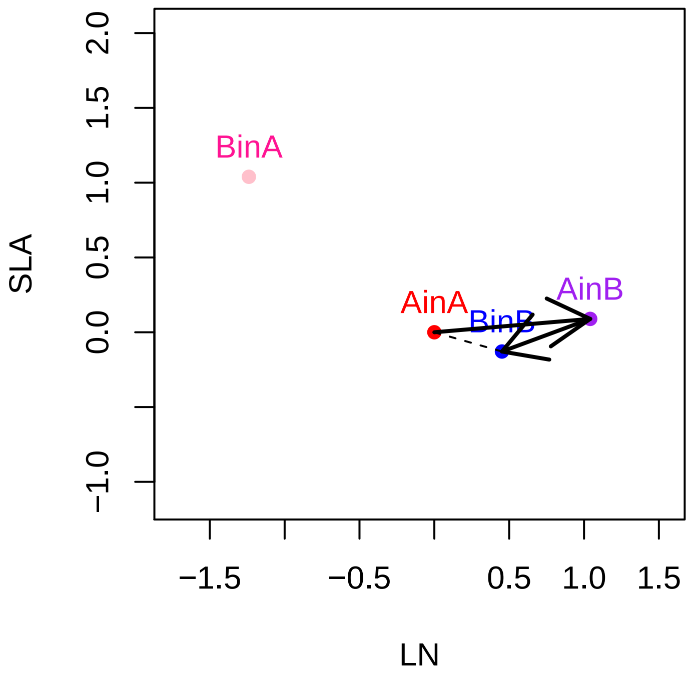

10 Etterson_2004 - comparing Oklahoma (OK) with Minnesota (MN)

The study species is *Chamaecrista fasciculata*. The angle between pasticity vector and evolutionary divergence is 77.77°. The angle between pasticity vector and total phenotypic difference is 11.35°.

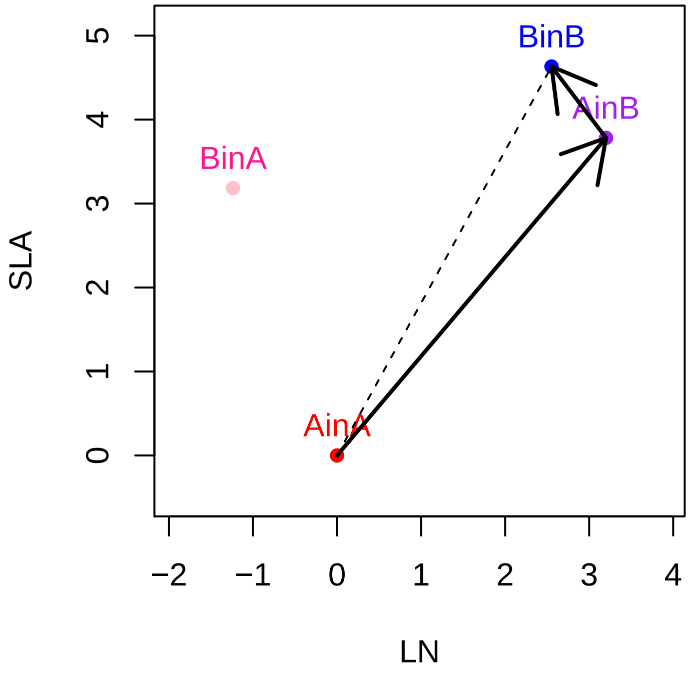

11 Etterson_2004 - comparing Oklahoma (OK) with Kansas (KS)

The study species is *Chamaecrista fasciculata*. The angle between pasticity vector and evolutionary divergence is 2.73°. The angle between pasticity vector and total phenotypic difference is 0.98°.

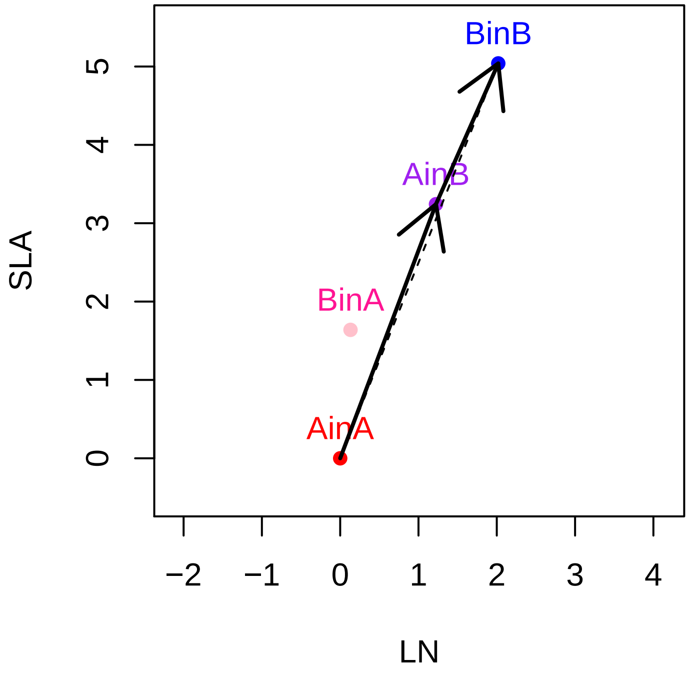

12 Griffith_Watson_2005 - comparing Southern with Intermediate

The study species is *Xanthium strumarium*. The angle between pasticity vector and evolutionary divergence is 172.37°. The angle between pasticity vector and total phenotypic difference is 166.35°.

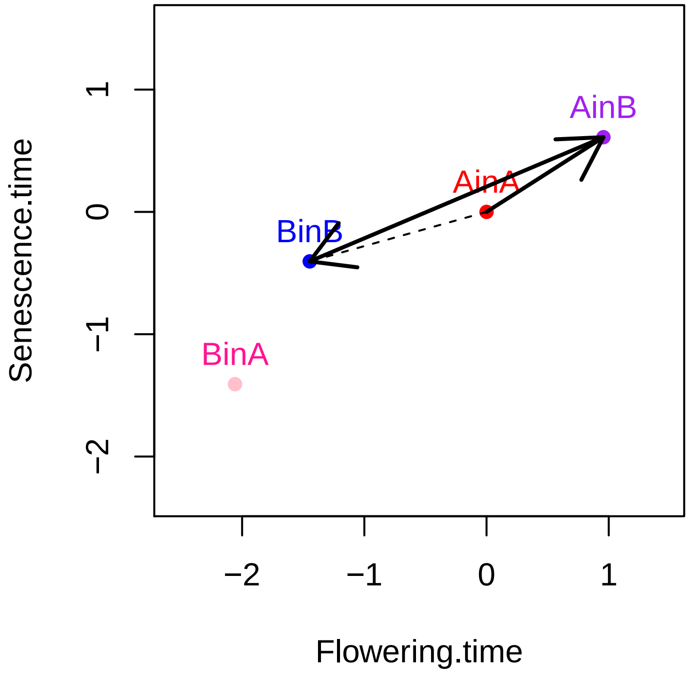

13 Griffith_Watson_2005 - comparing Southern with Range edge

The study species is *Xanthium strumarium*. The angle between pasticity vector and evolutionary divergence is 172.86°. The angle between pasticity vector and total phenotypic difference is 165.11°.

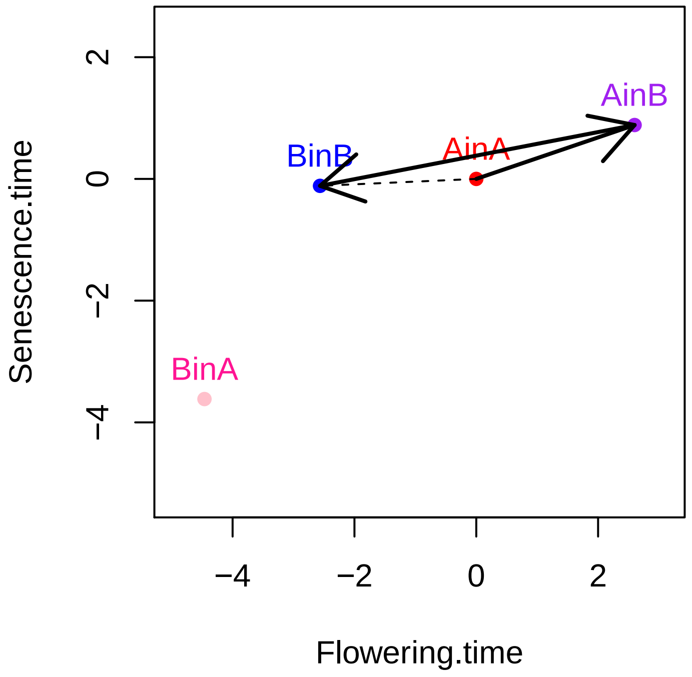

14 Griffith_Watson_2005 - comparing Intermediate with Range edge

The study species is *Xanthium strumarium*. The angle between pasticity vector and evolutionary divergence is 166.61°. The angle between pasticity vector and total phenotypic difference is 130.09°.

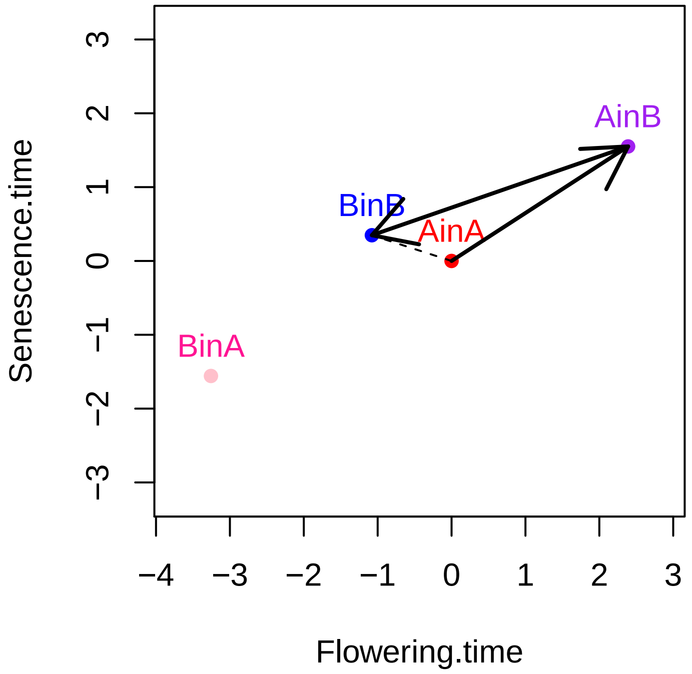

15 Nagy_Rice_1997 - comparing Inland with Coastal

The study species is *Gilia capitata*. The angle between pasticity vector and evolutionary divergence is 122.89°. The angle between pasticity vector and total phenotypic difference is 15.82°.

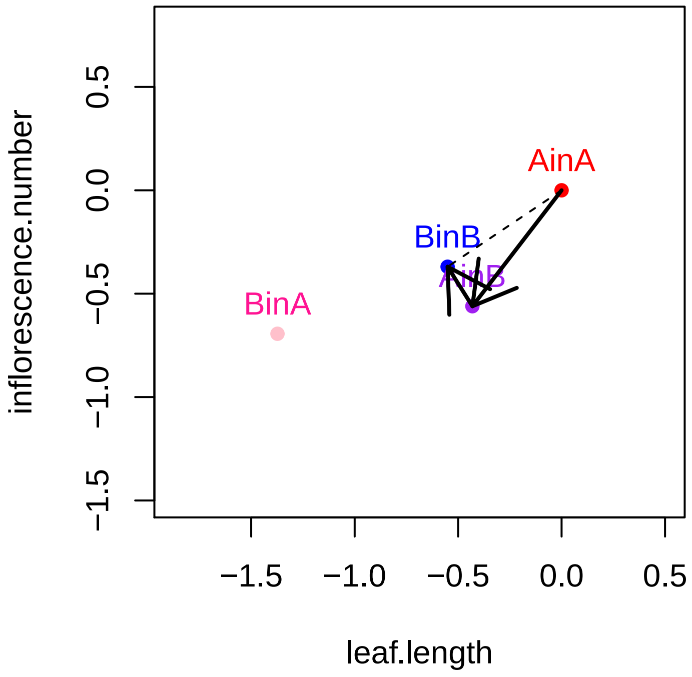

16 Bucharova_ea_2017b - comparing R7-Tuebingen with R3-Halle

The study species is *Arrhenatherum elatius*. The angle between pasticity vector and evolutionary divergence is 146.67°. The angle between pasticity vector and total phenotypic difference is 14.18°.

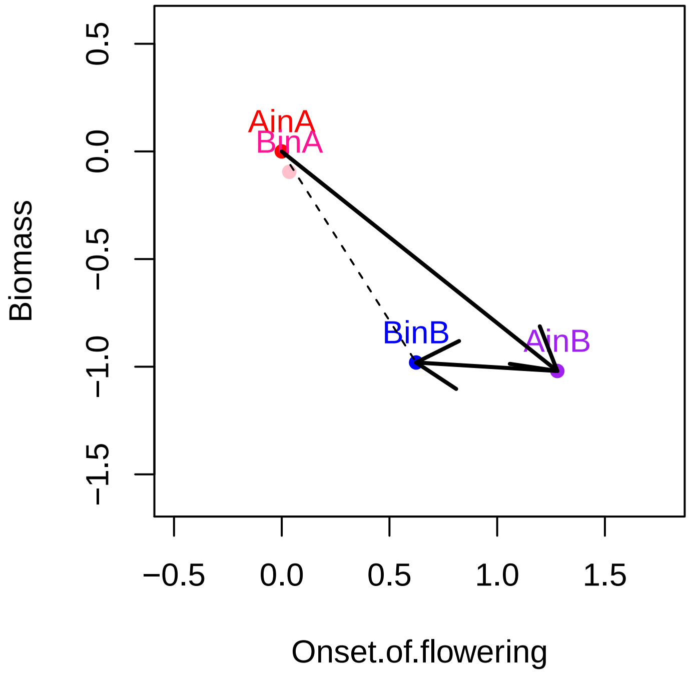

17 Bucharova_ea_2017b - comparing R8-Freiburg with R7-Tuebingen

The study species is *Centaurea jacea*. The angle between pasticity vector and evolutionary divergence is 166.27°. The angle between pasticity vector and total phenotypic difference is 20.9°.

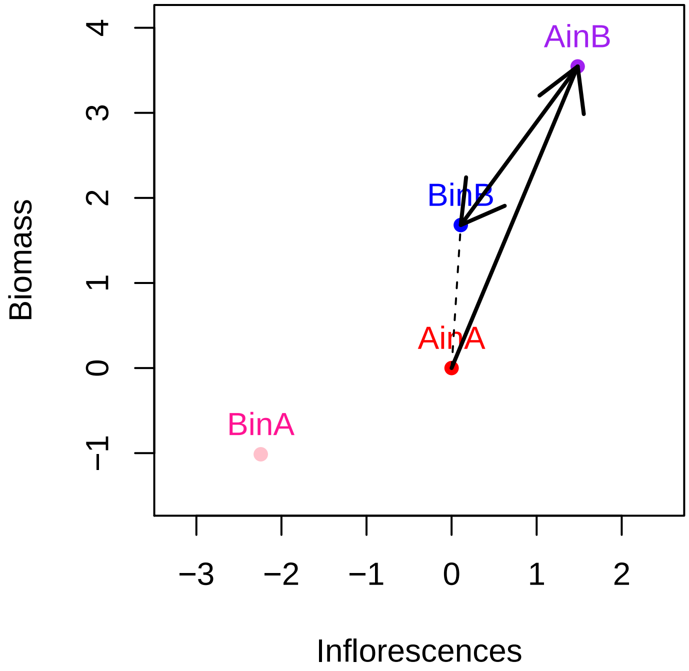

18 Bucharova_ea_2017b - comparing R3-Halle with R1-Muenster

The study species is *Hypochaeris radicata*. The angle between pasticity vector and evolutionary divergence is 86.42°. The angle between pasticity vector and total phenotypic difference is 20.63°.

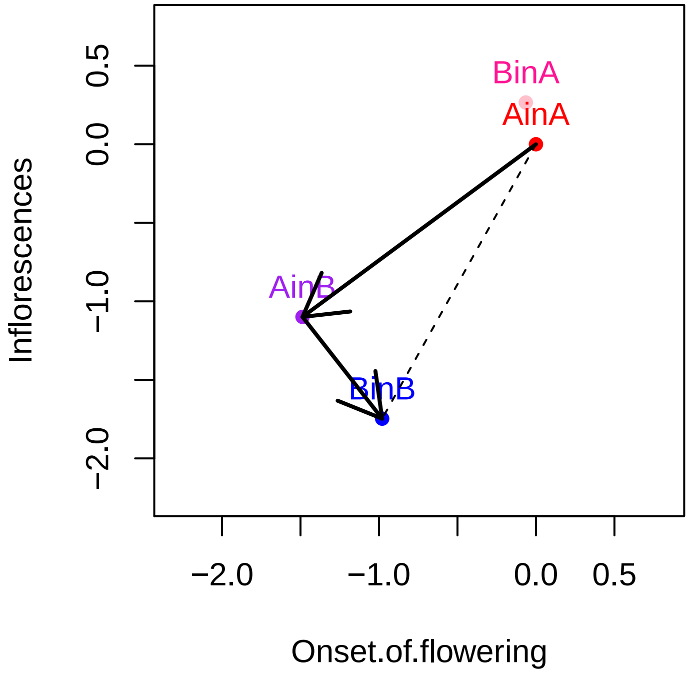

19 Bucharova_ea_2017b - comparing R8-Freiburg with R1-Muenster

The study species is *Hypochaeris radicata*. The angle between pasticity vector and evolutionary divergence is 156.19°. The angle between pasticity vector and total phenotypic difference is 13.07°.

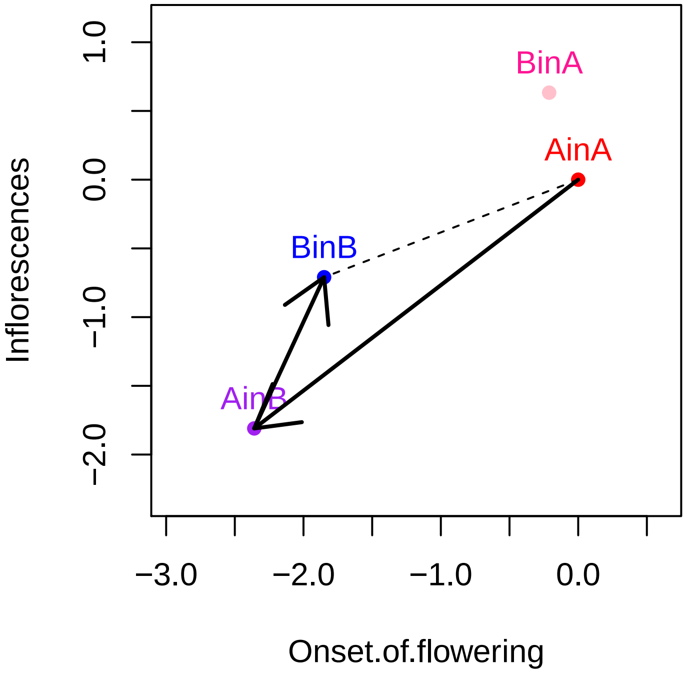

20 Bucharova_ea_2017b - comparing R8-Freiburg with R3-Halle

The study species is *Hypochaeris radicata*. The angle between pasticity vector and evolutionary divergence is 60.48°. The angle between pasticity vector and total phenotypic difference is 19.71°.

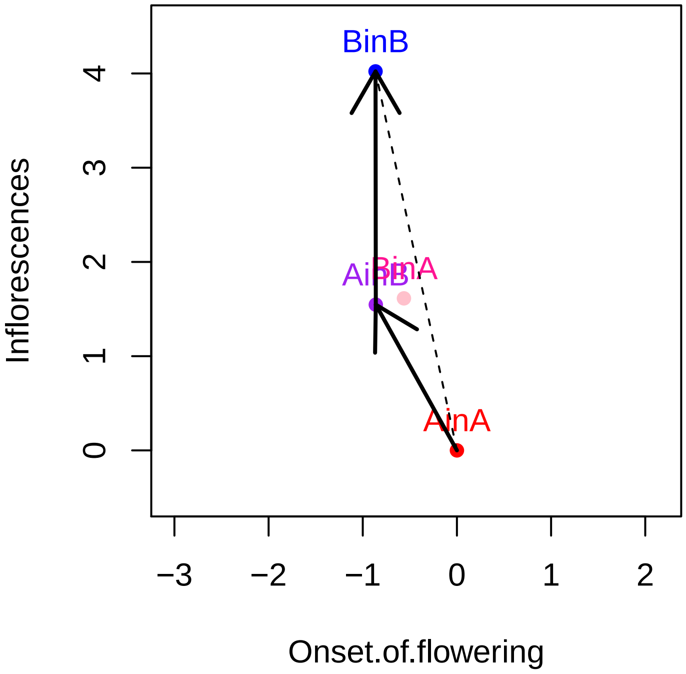

21 Dechamps_ea_2008 - comparing Nonmetallicolous with Metallicolous

The study species is *Thlaspi caerulescens*. The angle between pasticity vector and evolutionary divergence is 167.83°. The angle between pasticity vector and total phenotypic difference is 14.98°.

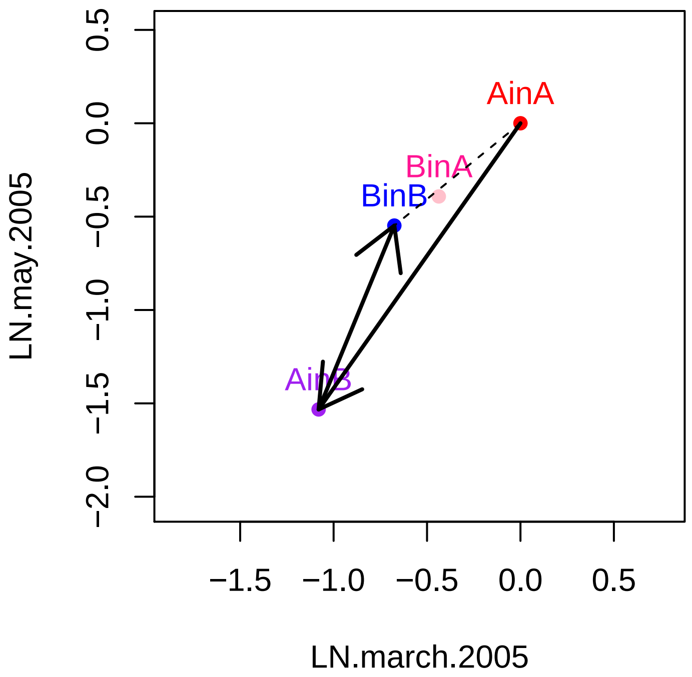

22 Nagamitsu_ea_2015 - comparing south with north

The study species is *Pinus densiflora*. The angle between pasticity vector and evolutionary divergence is 125.53°. The angle between pasticity vector and total phenotypic difference is 26.05°.

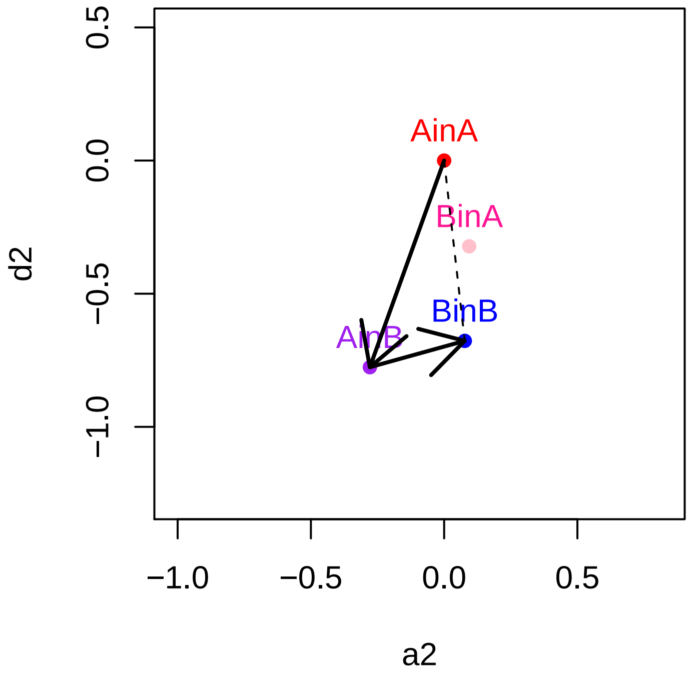

23 Urban_ea_2017 - comparing low risk with high risk

The study species is *Rana sylvatica*. The angle between pasticity vector and evolutionary divergence is 72.92°. The angle between pasticity vector and total phenotypic difference is 38.41°.

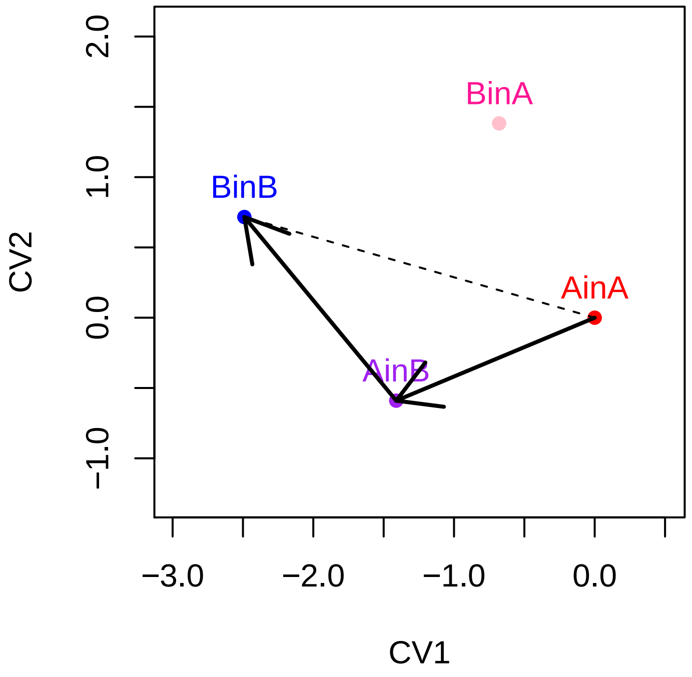

24 Walter_ea_2018 - comparing Table with Dune

The study species is *Senecio pinnatifolius*. The angle between pasticity vector and evolutionary divergence is 29.8°. The angle between pasticity vector and total phenotypic difference is 19.59°.

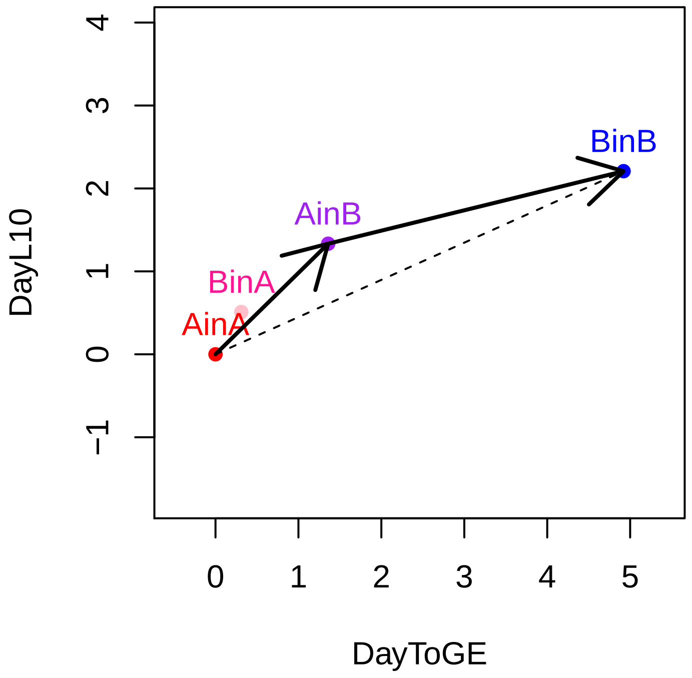

25 Walter_ea_2018 - comparing Wood with Dune

The study species is *Senecio pinnatifolius*. The angle between pasticity vector and evolutionary divergence is 30.15°. The angle between pasticity vector and total phenotypic difference is 19.11°.

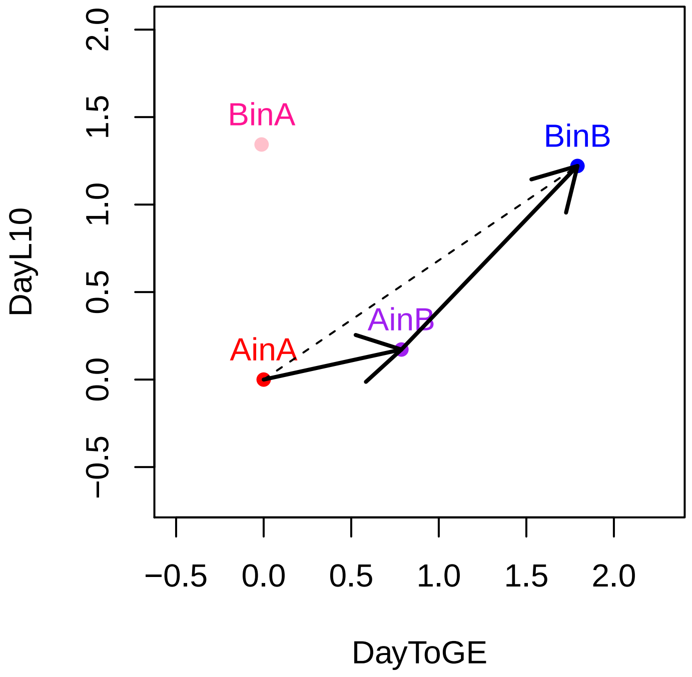

26 Walter_ea_2018 - comparing Wood with Head

The study species is *Senecio pinnatifolius*. The angle between pasticity vector and evolutionary divergence is 161.06°. The angle between pasticity vector and total phenotypic difference is 21.35°.

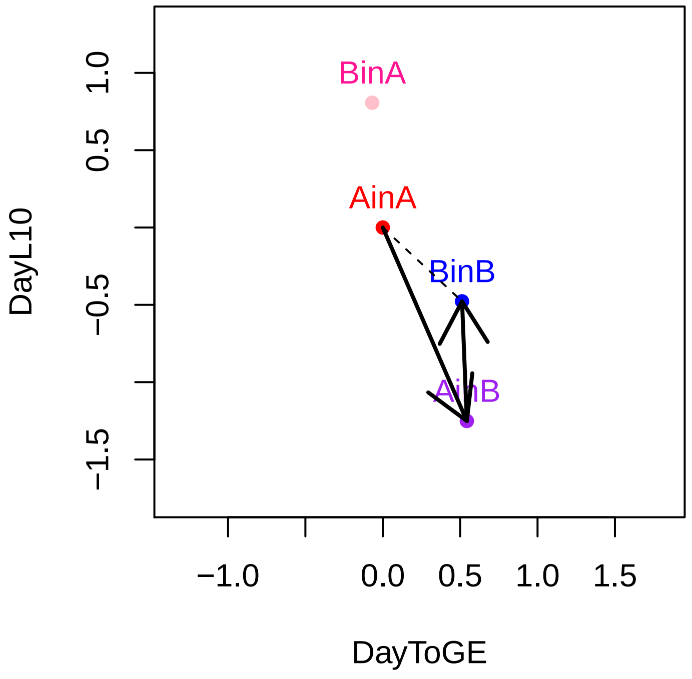

27 Walter_ea_2018 - comparing Wood with Table

The study species is *Senecio pinnatifolius*. The angle between pasticity vector and evolutionary divergence is 164.23°. The angle between pasticity vector and total phenotypic difference is 5.26°.

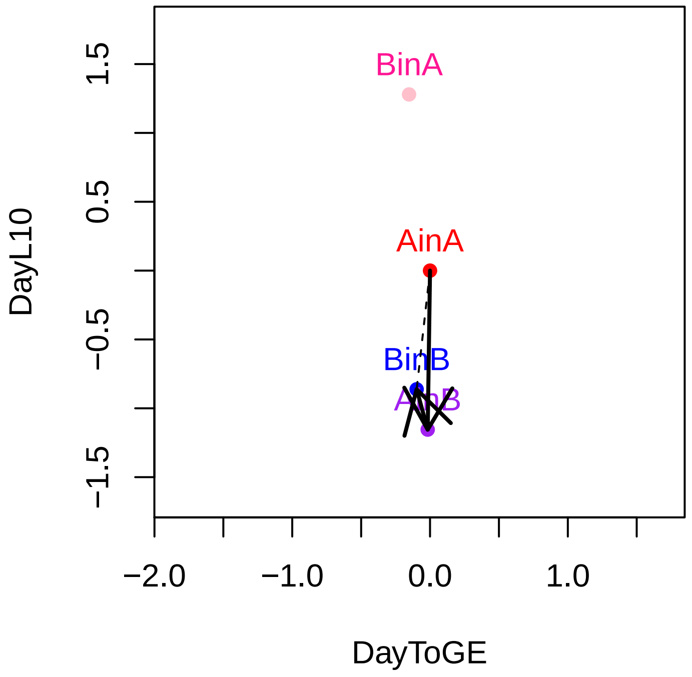

28 Wright_ea_2006 - comparing Non-Serpentine with Serpentine

The study species is *Collinsia sparsiflora*. The angle between pasticity vector and evolutionary divergence is 156.57°. The angle between pasticity vector and total phenotypic difference is 37.66°.

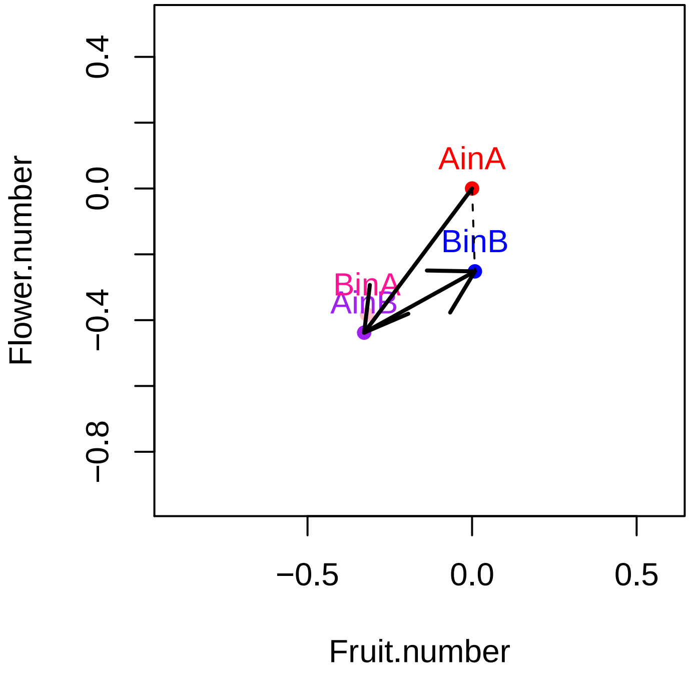

29 Bartheld__ea__2015 - comparing N - La Serena with S - Valdivia

The study species is *Cornu aspersum*. The angle between pasticity vector and evolutionary divergence is 125.17°. The angle between pasticity vector and total phenotypic difference is 107.59°.

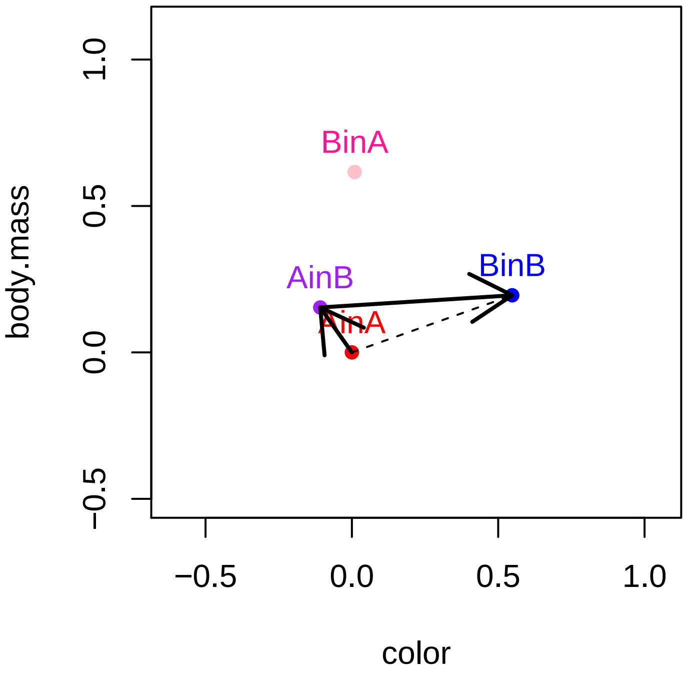

Supplement: Supplementary file 3 — Appendix S3: Plots of Empirical Examples [file EVL3-4-360-s003.pdf]
